# A Sensitive and Effective Proteomic Approach to Identify She-Donkey’s and Goat’s Milk Adulterations by MALDI-TOF MS Fingerprinting

**DOI:** 10.3390/ijms150813697

**Published:** 2014-08-08

**Authors:** Francesco Di Girolamo, Andrea Masotti, Guglielmo Salvatori, Margherita Scapaticci, Maurizio Muraca, Lorenza Putignani

**Affiliations:** 1 Department of Laboratory Medicine, Bambino Gesù Children’s Hospital, IRCCS, Piazza Sant’Onofrio 4, Rome 00165, Italy; E-Mail: maurizio.muraca@opbg.net; 2 Gene Expression-Microarrays Laboratory, Bambino Gesù Children’s Hospital, IRCCS, Piazza Sant’Onofrio 4, Rome 00165, Italy; E-Mail: andrea.masotti@opbg.net; 3 Neonatal Intensive Care Unit, Department of Medical and Surgical Neonatology, Bambino Gesù Children’s Hospital, IRCCS, Piazza Sant’Onofrio 4, Rome 00165, Italy; E-Mail: guglielmo.salvatori@opbg.net; 4 Department of Laboratory Medicine, San Camillo Hospital, Viale Vittorio Veneto 18, Treviso 31100, Italy; E-Mail: scapaticci.m@gmail.com; 5 Parasitology Unit, Bambino Gesù Children’s Hospital, IRCCS, Piazza Sant’Onofrio 4, Rome 00165, Italy; E-Mail: lorenza.putignani@opbg.net; 6 Metagenomics Unit, Bambino Gesù Children’s Hospital, IRCCS, Piazza Sant’Onofrio 4, Rome 00165, Italy

**Keywords:** milk, adulteration, MALDI-TOF MS

## Abstract

She-donkey’s milk (DM) and goat’s milk (GM) are commonly used in newborn and infant feeding because they are less allergenic than other milk types. It is, therefore, mandatory to avoid adulteration and contamination by other milk allergens, developing fast and efficient analytical methods to assess the authenticity of these precious nutrients. In this experimental work, a sensitive and robust matrix-assisted laser desorption/ionization time-of-flight mass spectrometry (MALDI-TOF MS) profiling was designed to assess the genuineness of DM and GM milks. This workflow allows the identification of DM and GM adulteration at levels of 0.5%, thus, representing a sensitive tool for milk adulteration analysis, if compared with other laborious and time-consuming analytical procedures.

## 1. Introduction

Milk is the most important nutrient for young mammals and is traditionally considered an ideal source of proteins and microelements for adult people. Confirming the popular tradition, recent clinical studies have clearly demonstrated that the she-donkey’s milk (DM) represents the best mother’s milk substitute for newborns, which are allergic to cow’s milk (CM) proteins, because of its low allergenic properties together with its high nutritional value [1].

Goat’s milk (GM) has also become popular in infant feeding because it is less allergenic than CM [2]; indeed, for infants suffering of CM-induced gastrointestinal allergy and chronic enteropathy, GM feeding has been reported [3].

Unfortunately, the intolerance based on the immunological response to CM proteins is the most common form of food allergy in the pediatric population (from 0.3% to 7.5% of affected children) [4].

For this reason, revealing adulterations of DM and GM is of fundamental importance for both economic and clinical reasons. To this aim, effective analytical methods to assess the genuineness of these milks are desirable. Currently, both immunological and chemical methods are used to detect milk and dairy product adulteration. The immunological methods are based on the recognition of antigens (*i.e.*, whole casein, β-casein and β-lactoglobulin) by specific antibodies [5,6,7]. However, even well-validated analytical techniques, such as the enzyme-linked immunosorbent assay (ELISA) and immunoblotting, are affected by false-positive results. On the other hand, chemical methods that are based on the detection of distinctive fatty acid and proteins, are very laborious and time-consuming. In fact, they rely on gas chromatography (GC) analyses for fatty acid characterization [8] and on gel or capillary electrophoresis, high-performance liquid chromatography (HPLC), and mass spectrometry (MS) for protein separation, identification and quantification [9,10]. Many MS methods have been developed for investigating protein profiles and structures in milk [11,12,13,14,15,16,17,18]. Matrix-assisted laser desorption/ionization time-of-flight MS (MALDI-TOF MS), owing to its simplicity of use and to the high reproducibility of the mass spectra, has become a powerful technique to obtain informative fingerprints of milk proteins. Moreover, as the MALDI-TOF MS analysis of a milk sample is able to reveal the characteristic peaks of the most abundant proteins, milk adulteration can be, therefore, assessed by identifying the additional protein peaks in the mass spectrum of the analyzed milk. In the last few years, the identification of whey proteins, such as α-lactalbumin and β-lactoglobulins, by MALDI-TOF MS have been employed by: (i) Cozzolino *et al.* [8] to reveal CM and ewe milk (EM) addition to fresh water buffalo mozzarella cheese with a limit of detection (LOD) below 5% and up to 2% respectively; (ii) Cozzolino *et al.* [19] to discover CM in EM and buffalo milk (BM) with a LOD below 5% and Consulo *et al.* [20] to identify CM or GM in DM with a LOD up to 2% and 0.5% respectively. Moreover, with the aim to identify possible fraudulent addition of CM to EM, employed for the production of commercial ewe cheese (Pecorino), Fanton *et al.* [21] described a MALDI-TOF MS method, able to assess the adulteration of ewe cheese by CM (LOD 10%), by detecting the presence of the γ2-casein mass peak. Following a bottom-up proteomic strategy (MS analysis of enzymatic digests of milk proteins), Chen *et al.* [10], and Calvano *et al.* [22], analyzed CM adulteration in GM by assessing β-lactoglobulin whey protein as the molecular marker, employing on-line HPLC electrospray MS (HPLC/ESI-MS) and MALDI-TOF MS, respectively (LOD 5%).

In this experimental work, we evaluated the performance of the total MALDI-TOF MS profiles, in the mass range 2000–25,000 Da, for the identification of DM and GM adulteration by CM, EM and BM. We have demonstrated that MALDI-TOF MS coupled to unsupervised hierarchical clustering, principal component (PCA) and Pearson’s correlation analyses represent a rapid, robust and very sensitive method, to reveal CM, EM, and BM adulteration in DM and GM at very low levels (down to 0.5%). Considering the high sensitivity that we obtained following this approach (more sensitive than the analytical method proposed by Consulo *et al.* [20]), we envisage that our analytical method could be successfully applied also for the evaluation of water buffalo mozzarella cheese, buffalo milk and ewe cheese (Pecorino) adulterations with a higher precision than that reported by Cozzolino *et al.* [8,19] and Fanton *et al.* [21].

## 2. Results and Discussion

### 2.1. Assessing the Performance of the MALDI-TOF MS Profiles in Discriminating Milk Species

**Figure 1:**
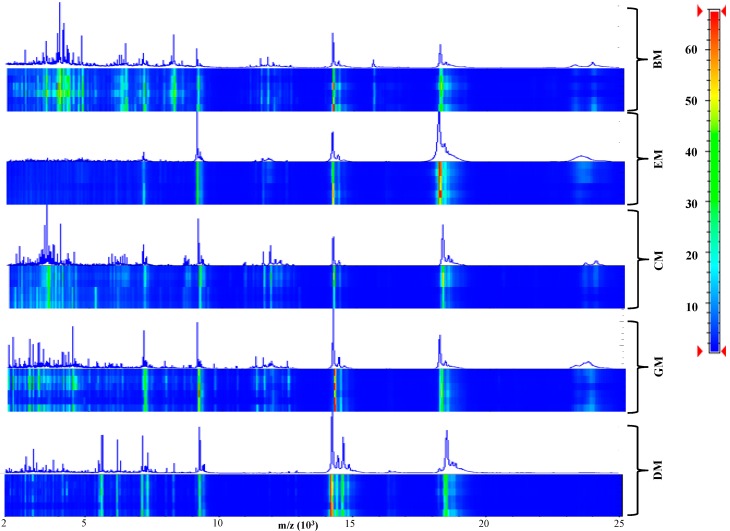
Pseudo-gel like and mass spectrometry (MS) proteomic profiling of crude milk from six she-donkey, goat, cow, ewe and buffalo animals. The mass values (*m*/*z*) are reported on the *X* axis, while the color bar on the *Y* axis indicates the peak intensity.

**Figure 2:**
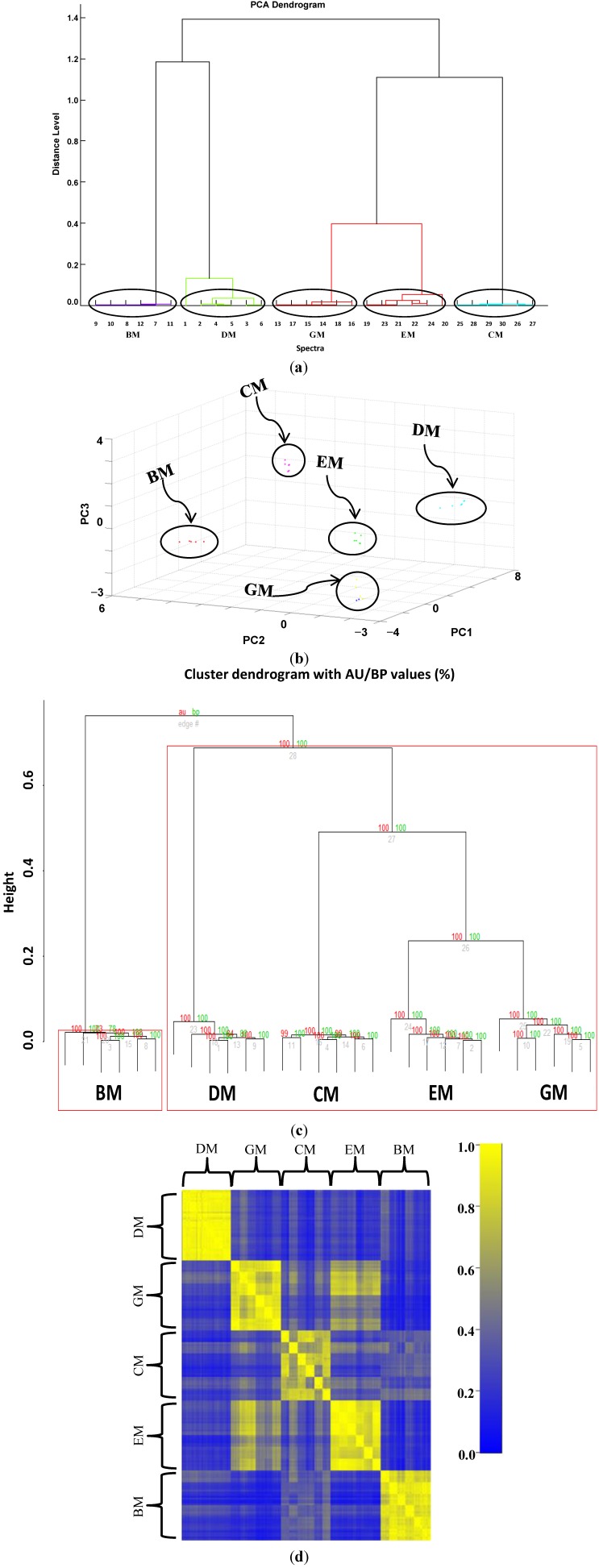
Data analysis of six independent matrix-assisted laser desorption/ionization time-of-flight mass spectrometry (MALDI-TOF MS)profiles of crude she-donkey’s milk (DM), goat’s milk (GM), cow’s milk (CM), ewe milk (EM) and buffalo milk (BM). (**a**) Hierarchical clustering dendrogram; (**b**) Principal component (PCA) analysis with corresponding 3D scatter plot image obtained by Biotyper; (**c**) Bootstrapped (*n* = 1000) hierarchical clustering tree generated via pvclust. Values on the edges of the clustering are *p*-values (%). Red values are AU (Approximately Unbiased) *p*-values, and green values are BP (Bootstrap Probability) values as explained in methods section. Clusters with AU larger than 95% are highlighted by red boxes and indicate reliable clades; (**d**) Pearson’s correlation coefficients represented as a correlation matrix. The correlation coefficients have been colored according to a scale ranging from 0 to 1, where blue corresponds to 0 and yellow to 1.

After acquisition of six independent MALDI-TOF MS samples from she-donkey’s, goat’s, cow’s, ewe’s, and buffalo’s crude milks, spectra were visually inspected (Figure 1) and the resulting flattened profiles were compared by gel-like representations, analyzed by unsupervised hierarchical clustering (Figure 2a) and PCA (Figure 2b) using the integrated software Biotyper 3.1 (Bruker Daltonics, Bremen, Germany), and finally by bootstrapped (*n* = 1000) clustering (Figure 2c) and correlation matrices (Figure 2d) with external statistical software (R Bioconductor, Fred Hutchinson Cancer Research Center, Seattle, WA, USA).

According to the dendrogram (Figure 2a) obtained by clustering the replicated profiles of the five milks and the PCA representation (Figure 2b), we found that the six mass spectra for each milk closely clustered together, whereas the different types of milk resulted well separated from each other. GM and EM displayed the lowest distance (*D* = 0.39) and clustered in the same clade, proximate to CM. BM and DM (*D* = 1.18) generated a second clade which in turn clustered with the larger clade formed by GM, EM and CM (*D* = 1.16). The bootstrapping procedure performed on external software (Figure 2c) confirmed the reliability of the Biotyper elaboration. The Pearson’s correlation matrix obtained from all the profiles displayed a high intra-group similarity and a low inter-group correlation (Figure 2d). GM and EM samples correlated quite well, and this result is in agreement also with clustering analysis that locates these two milks in the same clade.

All together these data indicate that the high reproducibility and discriminating power of MALDI-TOF MS analysis, coupled to a set of robust data analysis tools, allows a straightforward identification of the five types of milk and that the different types can be easily distinguished from each other.

### 2.2. Assessing the Performance of the MALDI-TOF MS Profiles in Discriminating She-Donkey’s Milk (DM) and Goat’s Milk (GM) Adulteration with Cow’s Milk (CM)

**Figure 3:**
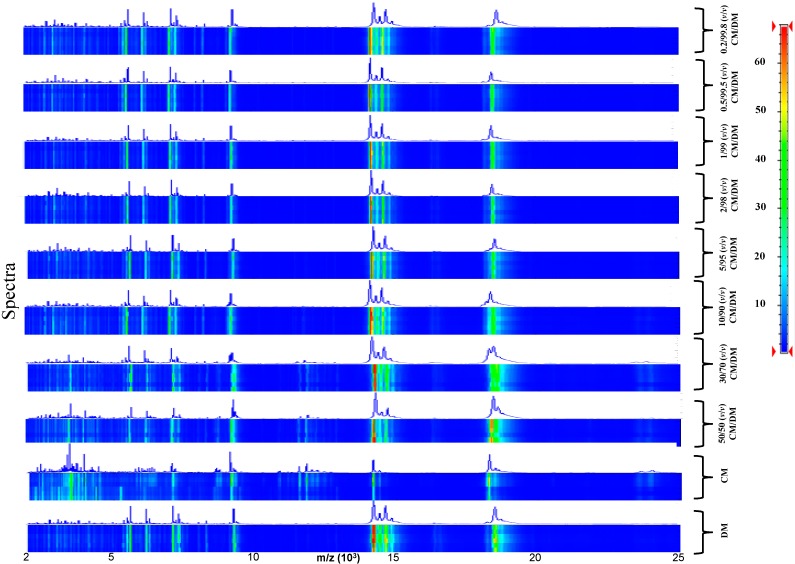
Pseudo-gel like image and MALDI-TOF MS spectra of the simulated adulteration of DM by CM.

**Figure 4:**
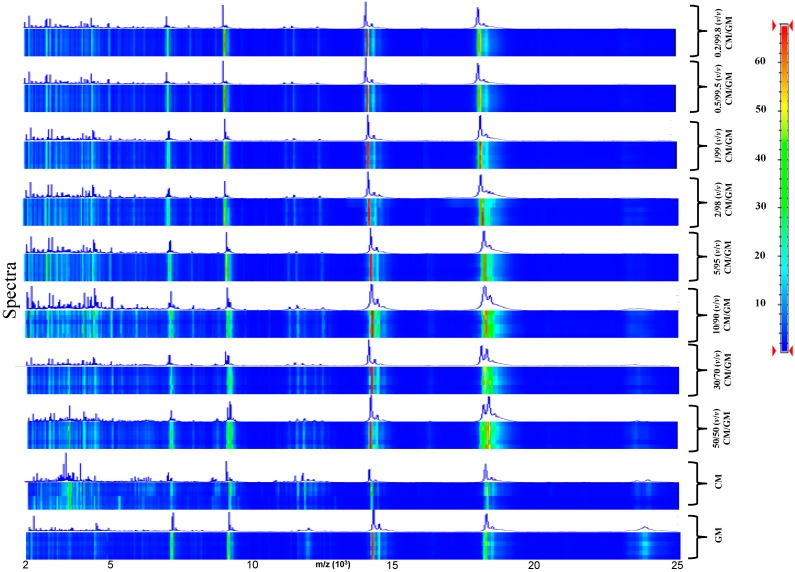
Pseudo-gel like image and MALDI-TOF MS spectra of the simulated adulteration of GM by CM.

To track milk adulteration, eight mixtures were prepared by adding CM at different percentages (0.2%, 0.5%, 1%, 2%, 5%, 10%, 30%, and 50% *v*/*v*) to DM (Figure 3) and GM (Figure 4). Six replicas for each sample were collected and analyzed.

**Figure 5:**
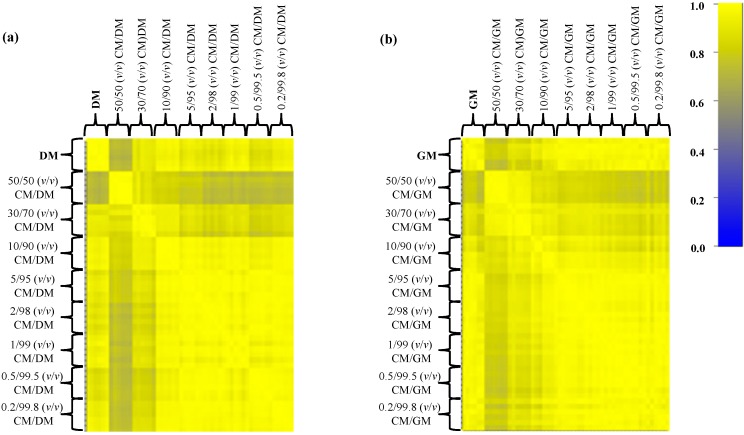
Pearson’s correlation matrices of all spectral replicates for the eight mixtures (from 50% to 0.2%) of simulated adulterations of (**a**) DM with CM and (**b**) GM with CM. Correlation coefficients are represented with decreasing blue and yellow colors according to a scale ranging from 0 to 1, respectively.

In this set-up the discriminant region of the MALDI-TOF MS profiles between 2000 and 25,000 kDa (Figure 3 and Figure 4) was very reproducible among the technical replicates and to gain further information about the correlation between the various mass profiles, we decided to perform a correlation analysis (Pearson’s coefficients) and represent the obtained results in a graphical representation. The Pearson’s correlation matrix representation for DM and GM adulteration with CM (Figure 5 and Table 1) displayed a high intra-class similarity as shown by the replicas in the diagonal, whereas the inter-class correlation appeared variable, as expected, owing to the different percentages of adulteration.

In particular, the native and the 50%-adulterated milks displayed a modest mean correlation value (0.720 for CM/DM and 0.782 for CM/GM), which gradually increased as the percentage of adulteration decreased (up to 0.2%–0.5%) (Table 1). The mean correlation coefficients that we obtained indicate that this simple analysis is not feasible to discern milk adulterations even at high percentages (10%–30%) as the correlation is rather good (0.961 for DM adulterated with CM at 10% *v*/*v*).

**Table 1:** The identification ability (expressed by the symbol √ = Yes, × = No) of different percentages of DM and GM adulteration obtained by our combined analytical and statistical approach; the correspondent correlation coefficients are also reported.

Adulteration	50/50 (*v*/*v*)	30/70 (*v*/*v*)	10/90 (*v*/*v*)	5/95 (*v*/*v*)	2/98 (*v*/*v*)	1/99 (*v*/*v*)	0.5/99.5 (*v*/*v*)	0.2/99.8 (*v*/*v*)
CM/DM	**√** 0.720	**√** 0.907	**√** 0.961	**√** 0.921	**√** 0.902	**√** 0.954	**√** 0.885	× 0.946
CM/GM	**√** 0.782	**√** 0.854	**√** 0.908	**√** 0.967	**√** 0.961	**√** 0.968	**√** 0.974	× 0.961

**Figure 6:**
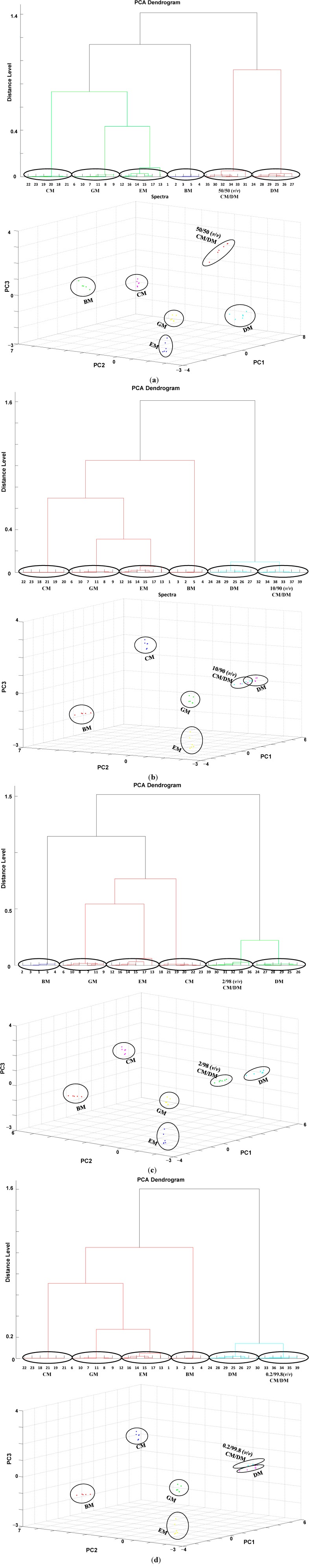
Hierarchical clustering dendrograms and principal component (PCA) 3D scatter plots of the first three principle components for the four mixtures (50%, 10%, 2%, and 0.2%) of the simulated adulteration of DM by CM (**Panels a**–**d**). All milk adulteration grouped inside the correct clade and cluster.

**Figure 7:**
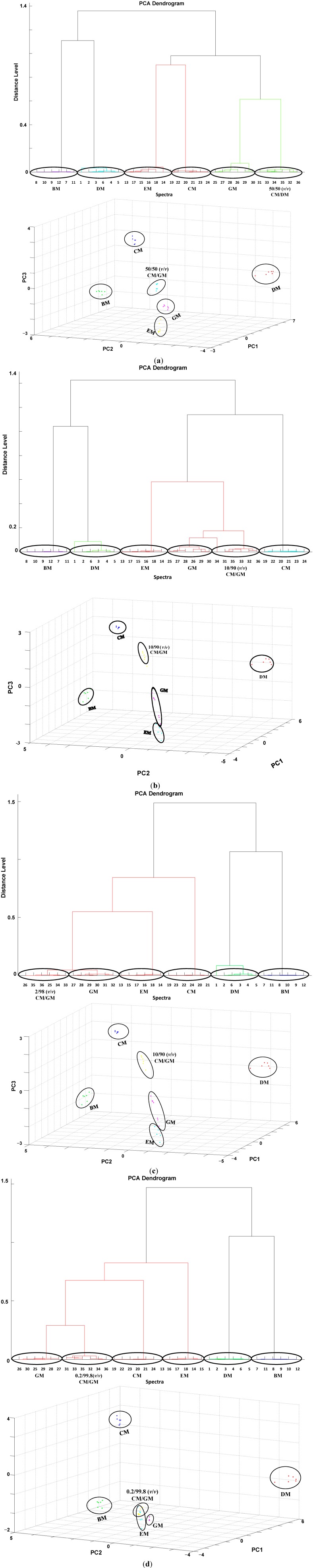
Hierarchical clustering dendrograms and PCA 3D scatter plots of the first three principle components for the four mixtures (50%, 10%, 2%, and0.2%) of the simulated adulteration of GM by CM (**Panels a**–**d**). All milk adulteration grouped inside the correct clade and cluster.

In order to increase the ability to discern milk adulterations up to a very low limit (0.2% of CM addition), we grouped all spectra within consistent clades and PCA clusters (Figure 6 and Figure 7).

Both hierarchical clustering and PCA analysis obtained with Biotyper outlined the good separation between the spectra belonging to the different percentages of adulteration in DM and GM, although with some differences.

In particular, hierarchical clustering is a 2D representation of the euclidean distance between the various spectra, whereas PCA is a 3D representation where suitable principal components can be properly selected to group similar samples. The advantage of PCA is the possibility to visually rotate the principal components graph for a better inspection of the acquired samples.

**Figure 8:**
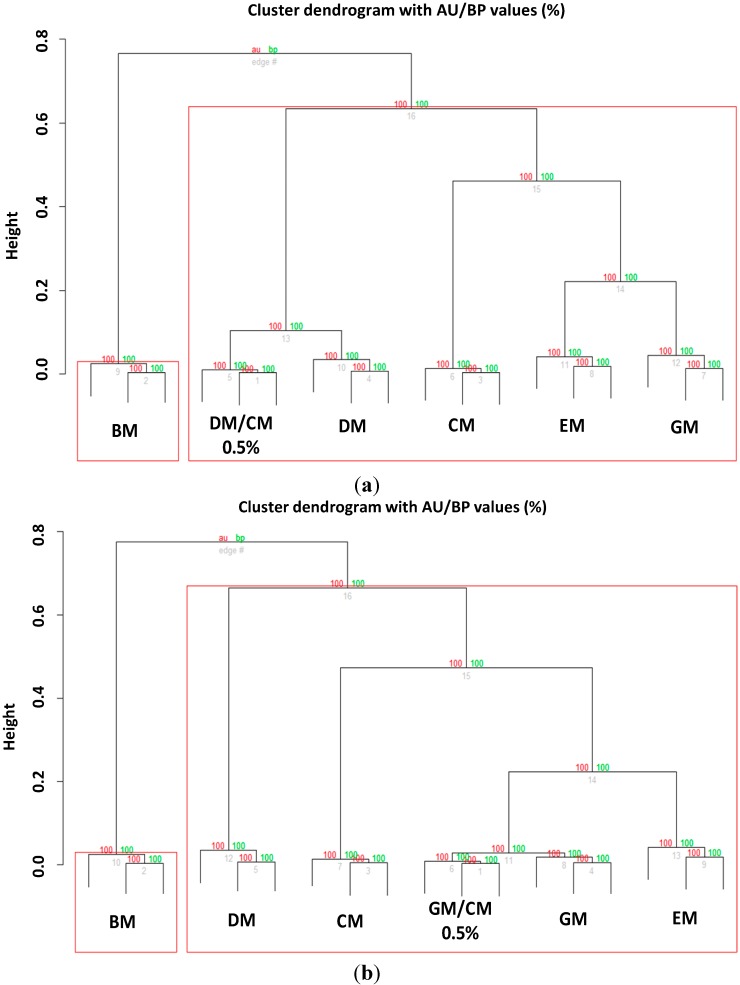
Bootstrapped (*n* = 1000) hierarchical clustering tree of (**a**) adulterated DM and (**b**) GM with CM (0.5% adulteration) generated via pvclust. Values on the edges of the clustering are *p*-values (%). Red values are AU (Approximately Unbiased) *p*-values, and green values are BP (Bootstrap Probability) values as explained in methods section. Clusters with AU larger than 95% are highlighted by red boxes and indicate reliable clades.

However, although Biotyper was able to discriminate adulterations up to a limit of 0.2%, the external clustering validation limited the recognition ability of our systems to a percentage of 0.5% for both DM (Figure 8a) and GM (Figure 8b).

**Figure 9:**
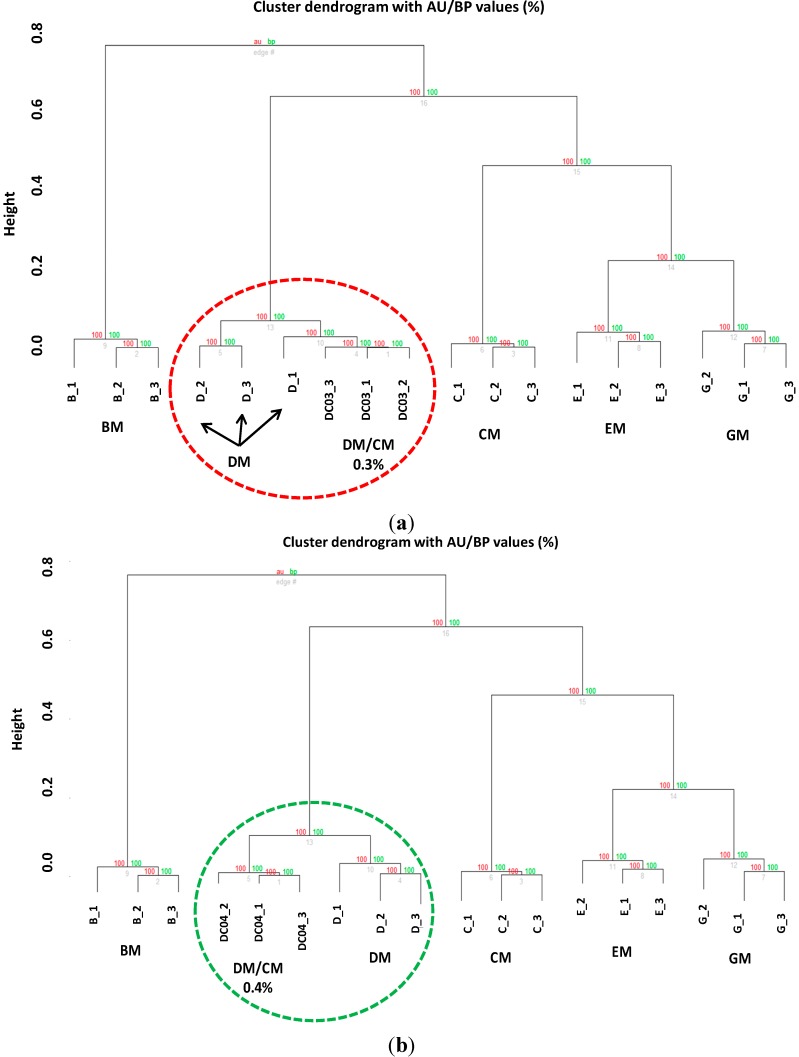
Hierarchical clustering tree of (**a**) adulterated DM with CM (0.3% *v*/*v*) and (**b**) DM with CM (0.4% *v*/*v*). Red circle indicates that the classification did not allow to obtain separate clades, whereas the green circle indicates that a good separation of the clades is obtained at an adulteration level of 0.4%.

To validate further the robustness of our analytical method, we prepared other seven DM samples added with different amounts (*v*/*v*) of CM (CM/DM mixtures: 0.3/99.7, 0.4/99.6, 0.7/99.3, 1.5/98.5, 9/91, 15/85, 40/60) and another investigator analyzed them (independent blind analysis). Again, although the researcher was able to identify all the adulteration levels by means of Biotyper software (Figure S1–S3) the in-depth statistical analysis revealed that the limit of detection is a little bit higher, as the 0.3% adulterated samples were not recognized correctly (Figure 9a) compared to 0.4% adulterated samples (Figure 9b).

This analytical method (using Biotyper 3.1 software) was also tested by using DM and GM samples treated by high (pasteurization) and ultra-high temperature (UHT) method. CM/DM and CM/GM mixtures (0.2/99.8, 10/90, and 50/50 volume ratios) were prepared and subsequently analyzed. Supplementary Figure S4 and S5 show that the method allowed the identification of adulterated milk as previously done with raw milk, suggesting that the proposed workflow is well-suited also for the analysis of commercial/market milks.

### 2.3. Assessing the Performance of the MALDI-TOF MS Profiles in Discriminating DM and GM Adulteration with EM and BM

We performed the same analysis also for DM and GM adulterated with other types of milk (EM and BM) obtaining similar results, briefly described here and illustrated in more details in the Supplementary section (Supplementary Figure S6–S8).

Briefly, correlation analysis (Pearson’s coefficients) emphasized once more that this kind of analysis is not useful to discriminate low percentage of adulteration. In fact, similarly to what obtained for DM and GM adulterated with CM, adulteration of DM and GM with EM and BM follows a similar behavior.

In particular, the native and the 50%-adulterated milks displayed a modest correlation value (from 0.583 for BM/GM to 0.846 for EM/GM), which gradually increased as the percentage of adulteration decreased (up to 0.2%–0.5%) (Table 2). Again, at adulteration percentages of about 10%–30% the values of the correlation coefficients are quite high (up to 0.959 for a 10% adulteration of DM with EM) indicating that the spectra are overall very well correlated. These results confirm that correlation analysis alone is not sufficient to discriminate efficiently milk adulteration.

Of note, from hierarchical clustering analysis (Biotyper software) of GM adulteration by EM at a percentage of 0.2% we found that the MALDI-TOF MS profiles grouped outside the correct clade and the PCA cluster, limiting the correct identification of adulteration of GM with EM at 0.5% mixtures. This is however a good results if we consider that the limit of detection generally obtained with other electrophoretic analyses is about 10%.

**Table 2:** The identification ability (expressed by the symbol √ = Yes, × = No) of different percentages of DM and GM adulteration obtained by the Biotyper analysis; the correspondent correlation coefficients are also reported.

Adulteration	50/50 (*v*/*v*)	30/70 (*v*/*v*)	10/90 (*v*/*v*)	5/95 (*v*/*v*)	2/98 (*v*/*v*)	1/99 (*v*/*v*)	0.5/99.5 (*v*/*v*)	0.2/99.8 (*v*/*v*)
EM/DM	**√** 0.664	**√** 0.804	**√** 0.959	**√** 0.974	**√** 0.984	**√** 0.967	**√** 0.888	**√** 0.895
EM/GM	**√** 0.846	**√** 0.902	**√** 0.891	**√** 0.823	**√** 0.871	**√** 0.790	**√** 0.878	× 0.964
BM/DM	**√** 0.644	**√** 0.813	**√** 0.948	**√** 0.969	**√** 0.954	**√** 0.983	**√** 0.978	**√** 0.957
BM/GM	**√** 0.583	**√** 0.754	**√** 0.822	**√** 0.876	**√** 0.873	**√** 0.805	**√** 0.828	**√** 0.778

## 3. Experimental Section

### 3.1. Milk Sampling and Preparation

The following samples were analyzed: (i) raw GM, DM, CM, EM, and BM collected from Italian farms (Lazio and Puglia); and (ii) adulterated samples made of CM/GM, EM/GM, BM/GM, CM/DM, EM/DM, and BM/DM mixtures, in volume ratios 0.2/99.8, 0.5/99.5, 1/99, 2/98, 5/95, 10/90, 30/70, and 50/50. Raw GM (from six goats belonging to Maltese breed), DM (from six she-donkeys belonging to Amiata, Viterbese and Martina Franca breeds), CM (from six cows belonging to Frisona breed), EM (from six ewes belonging to Tuscolania breed), and BM (from six buffalos belonging to Mediterranean Italian breed) samples were collected at middle lactation stage (125 ± 20 days), from two Italian herds located in Lazio and Puglia. Raw milk samples were mechanically collected into sterile polystyrene containers, immediately frozen, and stored at −80 °C until use, to prevent undesired proteolysis. After thawing, raw milk samples were defatted by centrifugation (using the Eppendorf Centrifuge 5417 R) and analyzed by linear MALDI-TOF MS for the generation of proteomic phenotyping profiles. The milk was defatted by two-step centrifugation (3000× *g*, 10 min) at 4 °C and the skimmed milk was centrifuged (20,000× *g*, 20 min) at 4 °C to remove bacteria and cell debris. The skimmed milk’s fractions were subsequently diluted 1:100 with ultrapure water (Milli-Q Millipore) and analyzed by linear MALDI-TOF MS as reported in the next paragraph.

### 3.2. MALDI-TOF MS Spectra Acquisition

One microliter of diluted skim milk was placed onto an MSP 96 polished steel target (Bruker Daltonics, Bremen, Germany) and allowed to dry at room temperature. Each sample was overlaid with 1 µL of matrix, which consisted of a solution of 10 mg/mL of sinapinic acid (Sigma–Aldrich, St. Louis, MO, USA) in 50% acetonitrile-0.1% trifluoroacetic acid (Sigma–Aldrich). Measurements were performed with a Microflex LT linear mass spectrometer (Bruker Daltonics), using FlexControl software package (version 3.0 Bruker Daltonics) [23]. Spectra were recorded in the positive linear mode (laser frequency, 20 Hz; ion source 1 voltage, 20 kV; ion source 2 voltage, 18.4 kV; lens voltage, 9.1 kV; mass range, 2000 to 25,000 Da). Six independent spectra (500 shots one step from different positions of the target spot, for spectrum) for each skimmed milk’s fraction were manually collected, externally calibrated by using Bacterial Test Standard (Bruker Daltonics) and subsequently analyzed.

### 3.3. MALDI-TOF MS Spectra Analysis

The entire set of data considered in this work consists in a collection of 468 MS spectra (six animals for 5 milk species for six spectra replicas and, 8 different percentage of adulteration for each adulterated sample’s mixture for six spectra replicas). MS spectra were manually acquired, and visually inspected before statistical analysis.

Spectra were loaded into FlexAnalysis software, version 3.0, (Bruker Daltonics) and preprocessed for: (i) mass adjustment, spectra were compressed by a factor of 10 in the total mass range; (ii) smoothing, mass data were adjusted by the Savitsky–Golay algorithm with a frame size of 25 Da; (iii) baseline subtraction, was applied the minimum value for finding the baseline; (iv) normalization, was applied the maximum morm to normalize the baseline subtracted data; and (v) peak picking, was applied spectra differentiation algorithm for finding the peaks, maximum peaks 100, threshold 0.1, method peak fitting. The entire preprocessed raw datasets of the 180 spectra replicas from the milk samples and 288 spectra replicas from the simulated fraudulent addition of DM and GM by CM, EM and BM were imported in R Bioconductor [24] for correlation matrices calculation (Pearson’s correlation) and hierarchical clustering. To gain confidence on the structure of the tree, we used the resampling package pvclust. For each cluster in hierarchical clustering, *p*-values (between 0 and 1) are calculated via multiscale bootstrap resampling. The package *pvclust* provides two types of *p*-values: AU (Approximately Unbiased) and BP (Bootstrap Probability). AU, which is computed by multiscale bootstrap resampling, is a better approximation to unbiased *p*-value than BP value computed by normal bootstrap resampling. The same set of spectra, were imported in Biotyper 3.1 software and converted into a virtual gel (pseudo-gel-like) format. The mass values (*m*/*z*) were reported on the *X* axis, while the color bar, reported on the *Y* axis, showed the relationship between the color and the pick intensity.

To visualize differences between milk and milk-adulterated populations, hierarchical clustering analysis was performed by principal component analysis (PCA) and dendrogram creation, via the external MATLAB (Matrix Laboratory) tool integrated in the MALDI Biotyper (Bruker Daltonik GmbH, Bremen, Germany).

In order to the PCA clustering method, we used the following parameters: Method, hierarchical; distance measure, correlation; linkage algorithm, average.

## 4. Conclusions

The detection of fraudulent adulterations and unintended contaminations of DM and GM by fast and efficient analytical methods is a matter of fundamental importance because many allergic children are fed with these fundamental nutrients. To this purpose, in this work we presented a suitable analytical method based on MALDI-TOF MS coupled to a robust statistical analysis (hierarchical clustering and PCA analysis) that provides substantial advances in the typing of milk and allows the investigation of the commonest types of adulterations in a straightforward, accurate and sensitive way.

This approach does not involve laborious pre-analytical sample separation steps and represents a fast reliable and robust method for routine analyses also for dairy industry. We would like to emphasize that although the Biotyper analysis allows to recognize adulterated milks (when compared with other types of milk) made by low percentage (even below 0.5%) of adulterant, a *post hoc* refinement of the analysis (bootstrapping analysis) indicated that the method is reliable for recognition of samples adulterated up to 0.5%. Overall, these results suggest that MALDI-TOF spectra of milk samples preliminarily analyzed by the integrated Biotyper software, allows a rapid determination of specimens adulterated even at low percentage of adulterant. Of note, the suggested workflow is far from being a quantitative analytical method, as the discrimination of classes of samples at different percentages of adulteration is still not possible with the analytical and statistical methods employed in this work.

Finally, the analytical strategy herein described is highly sensitive and robust compared to the techniques employed and reported so far in the literature and allows the qualitative identification of adulterated DM and GM milks obtained by the fraudulent addition of other types of milk up to a limit of 0.5%.

## Supplementary Files

Supplementary File 1
