# Supplementary material for: A Sensitive and Effective Proteomic Approach to Identify She-Donkey’s and Goat’s Milk Adulterations by MALDI-TOF MS Fingerprinting
Source: Int J Mol Sci. 2014 Aug 8;15(8):13697–719. doi: 10.3390/ijms150813697 (PMC4159820; doi:10.3390/ijms150813697)

Supplementary Information

Figures S1. For the validation of the analytical method, three best reproducible spectra were chosen for each milk species and, a dendrogram reference was produced (**Panels a**). CM/DM mixtures, in volume ratios 0.3/99.7, 0.4/99.6, 0.7/99.3, 1.5/98.5, 9/91, 15/85, 40/60 were prepared and a blinded investigator analyzed them. **Panels a–h**, shows a hierarchical clustering analysis, producing dendrograms. Figures S1 shows clearly the high performance of the analytical method in the identification of all the adulteration levels (from 0.3% to 40% of CM addition) for all the test samples prepared.

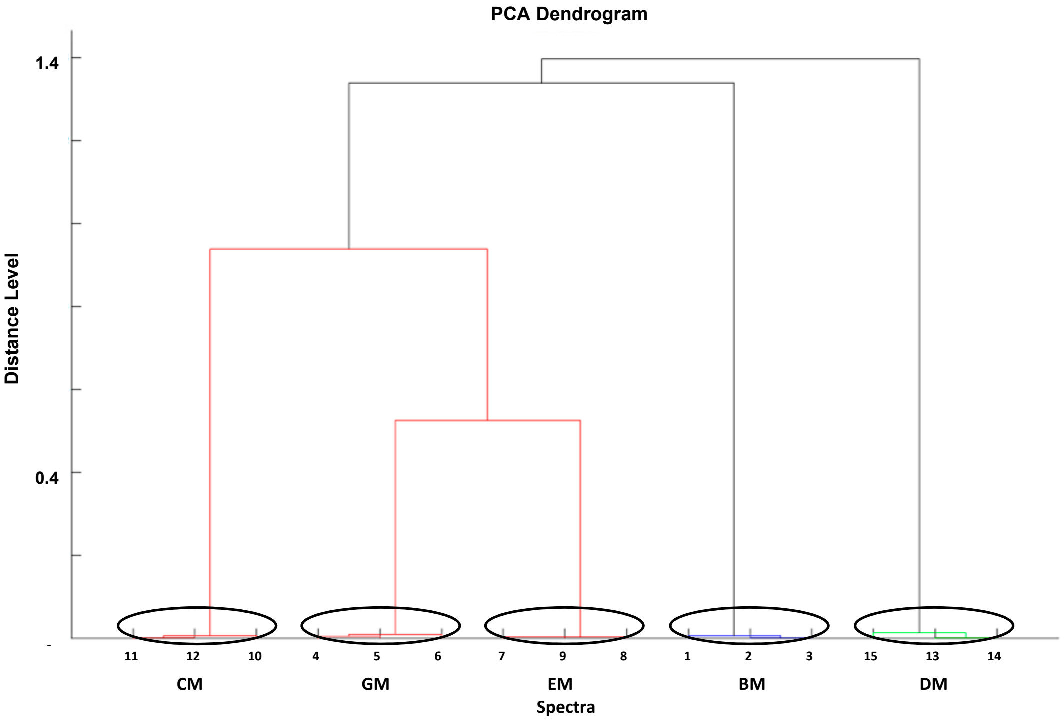

(a)

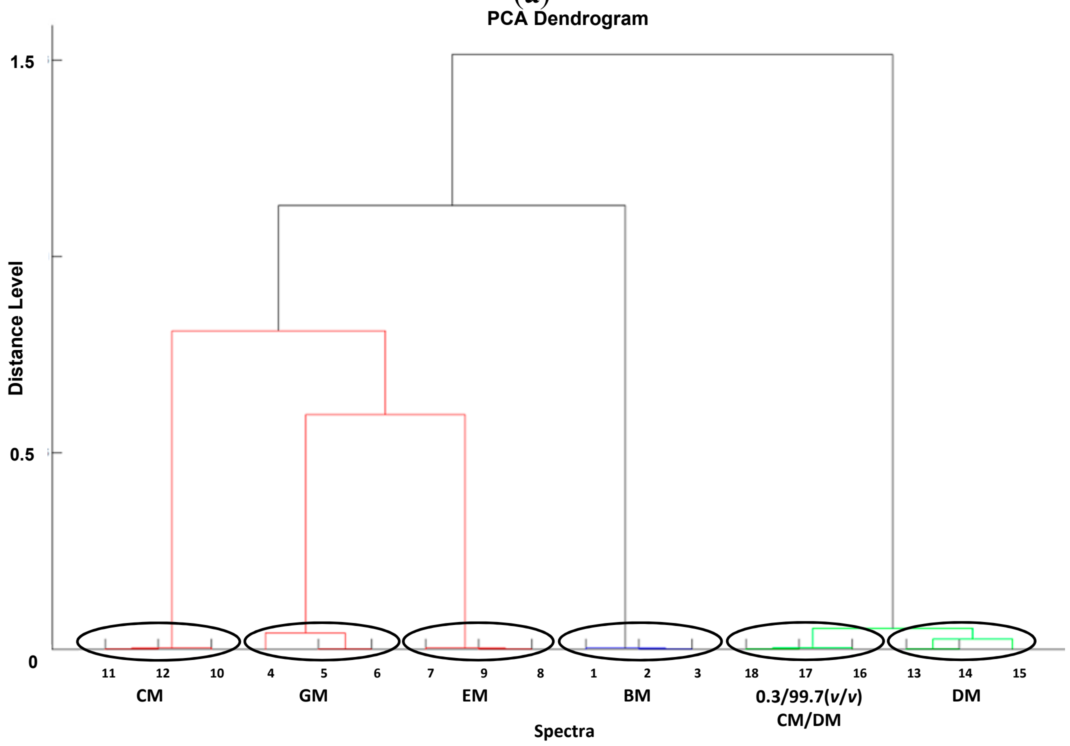

(b)

Figure S1. *Cont.*

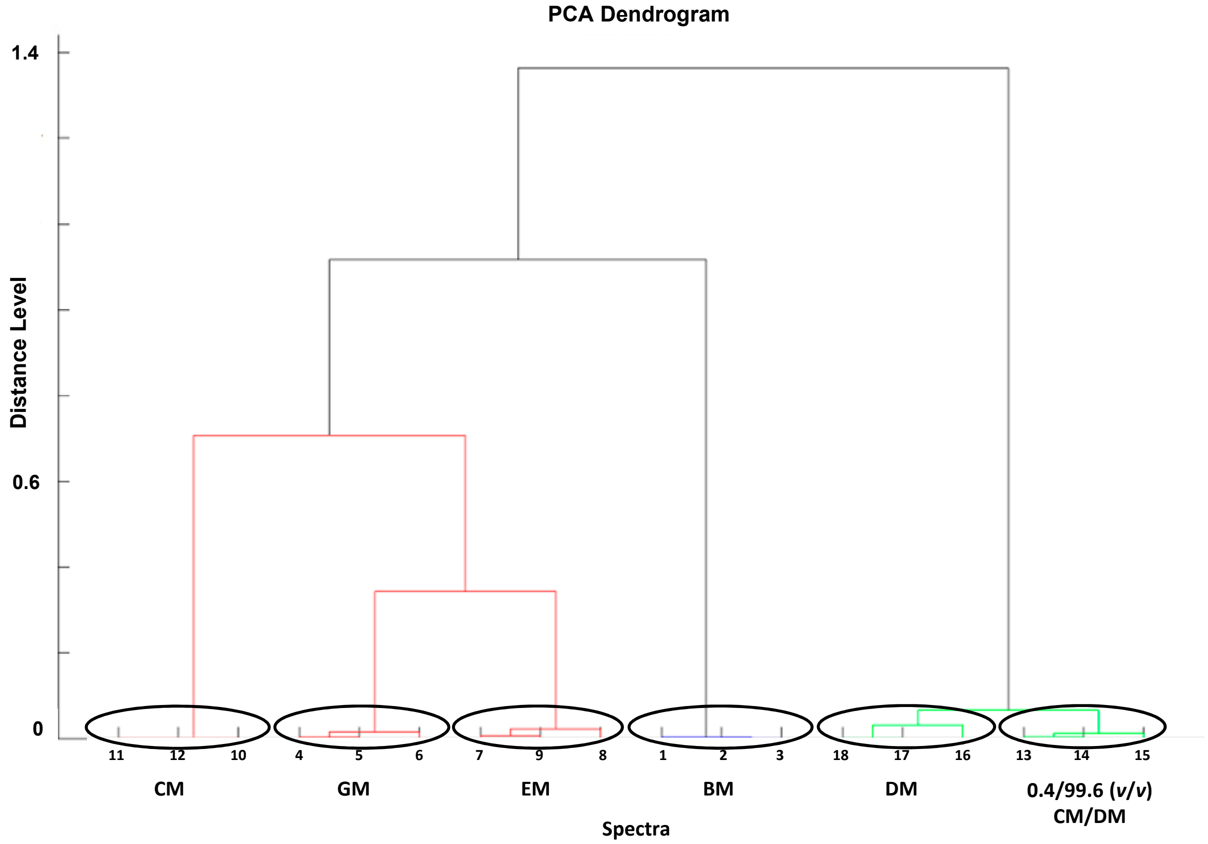

(c)

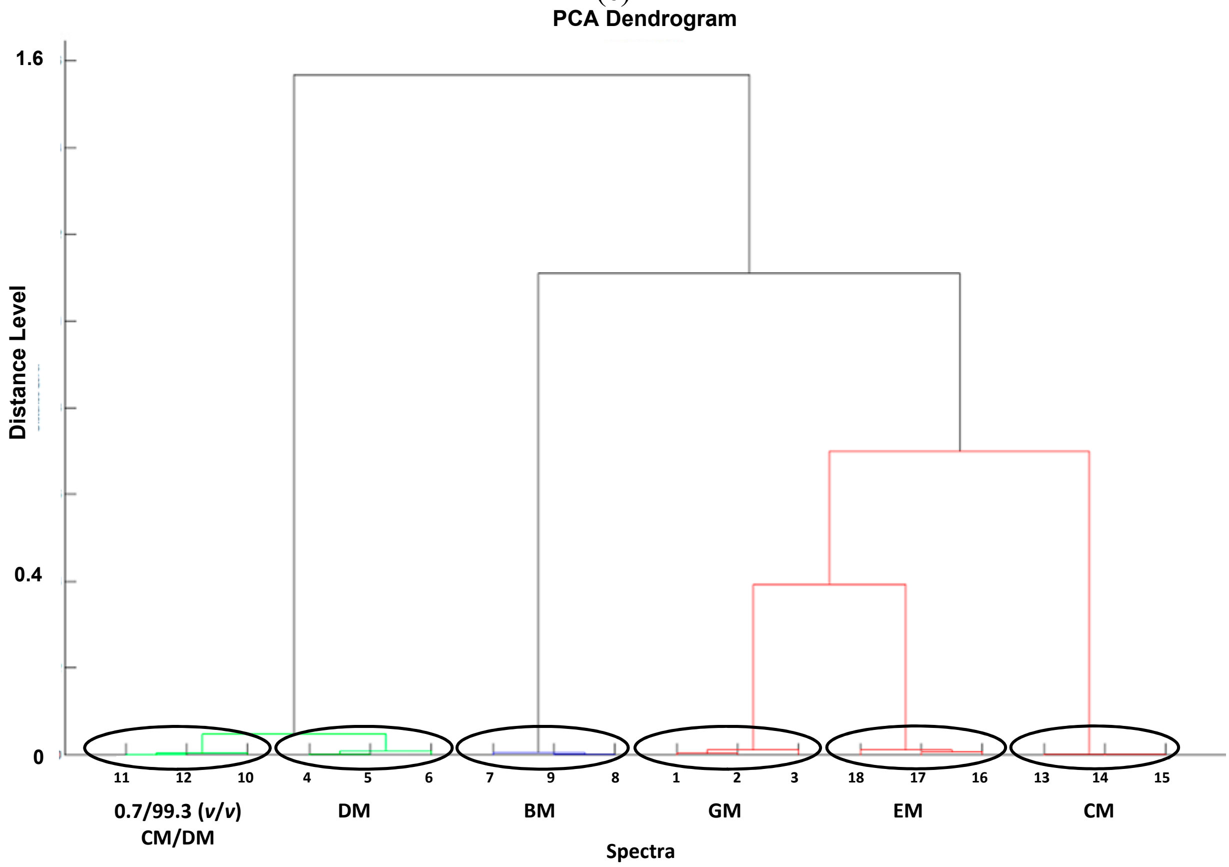

(d)

Figure S1. *Cont.*

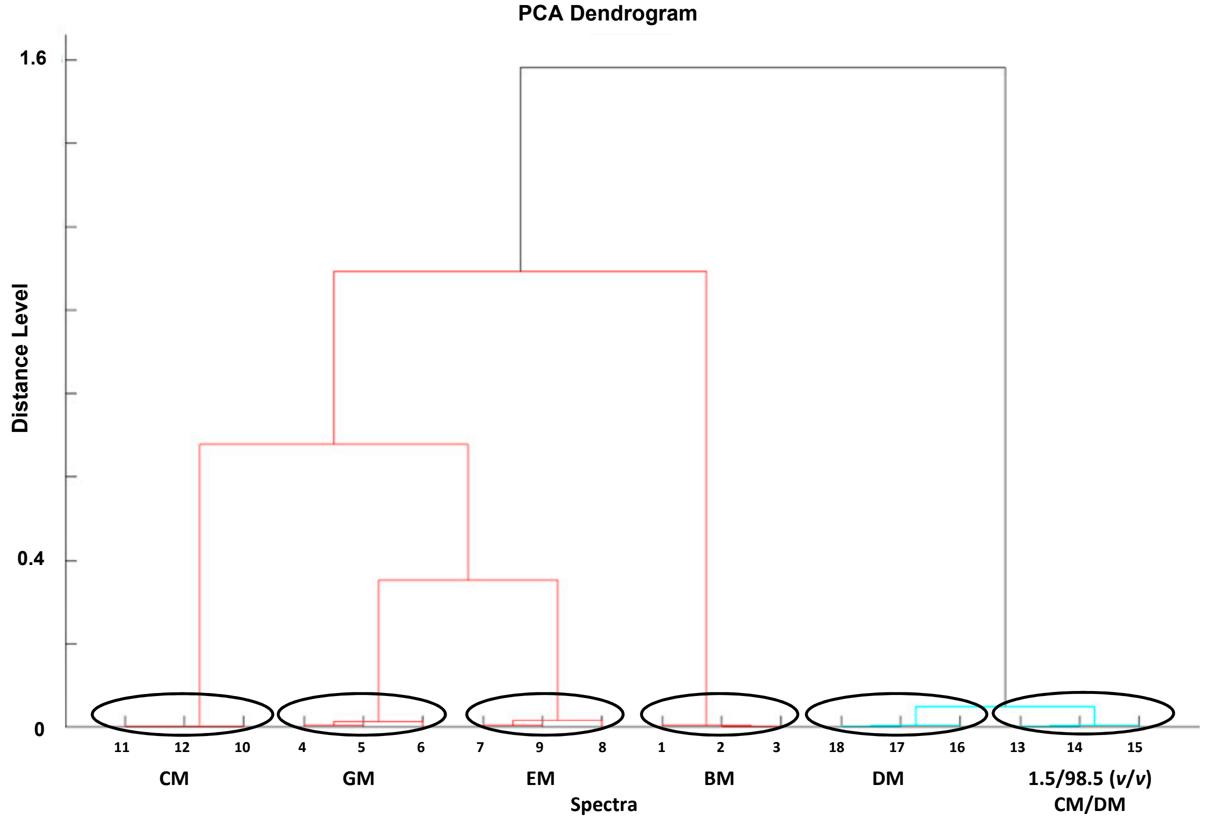

(**e**)

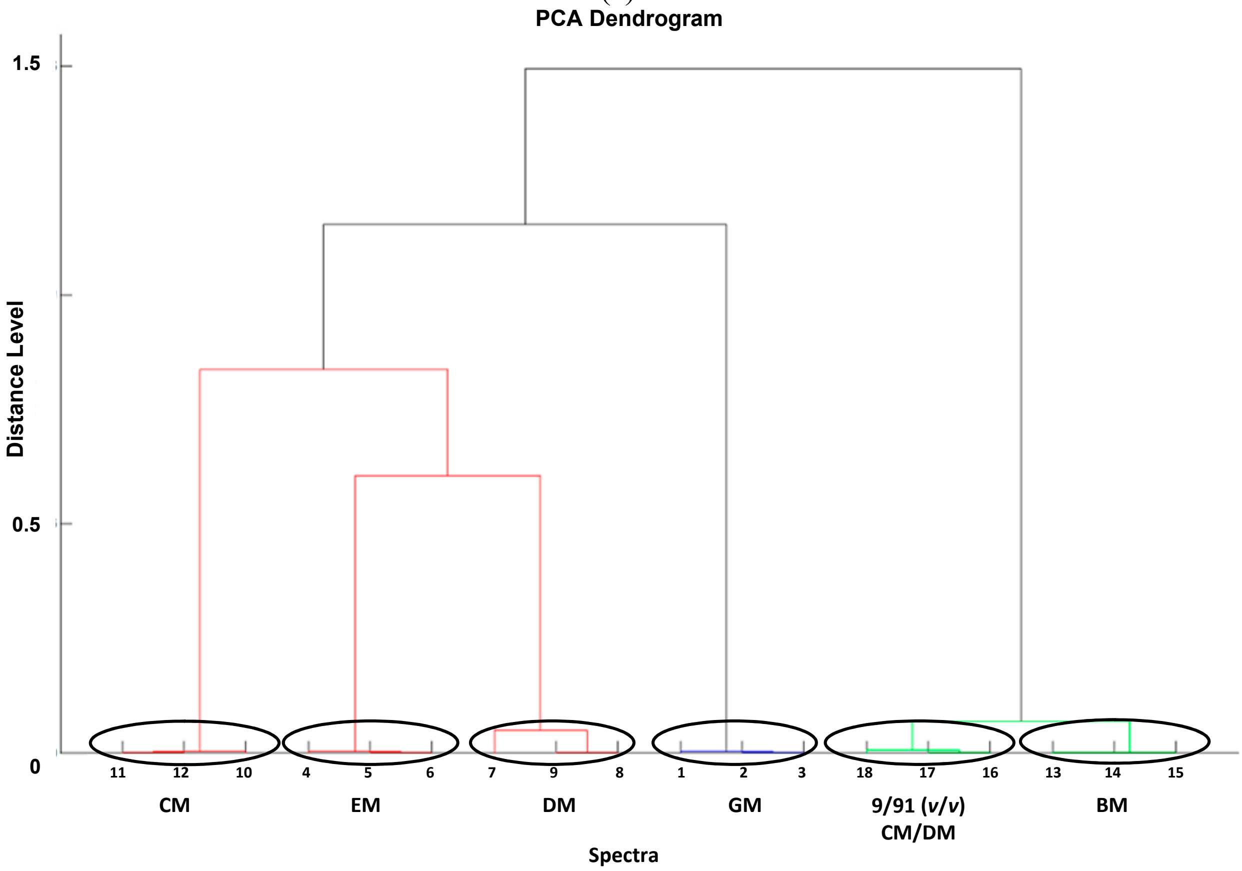

(**f**)

Figure S1. *Cont.*

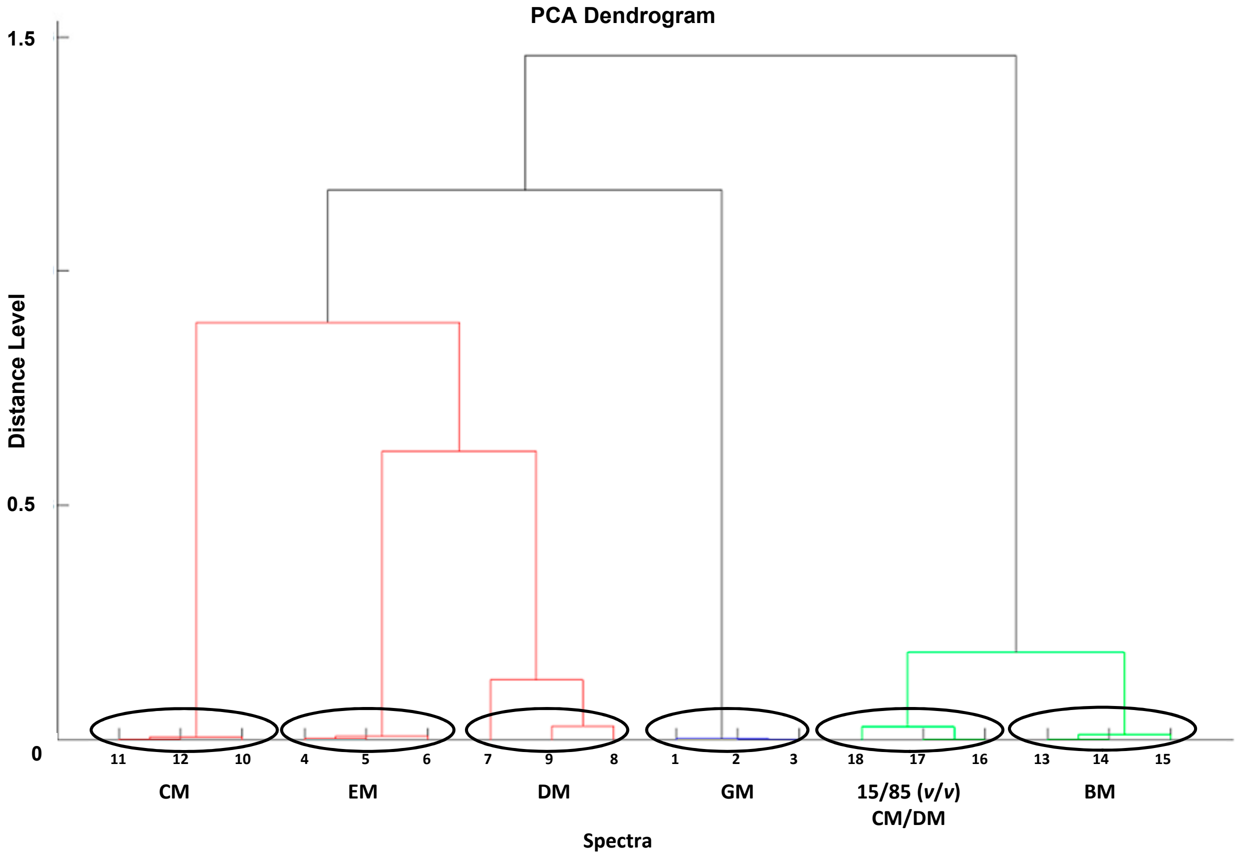

(g)

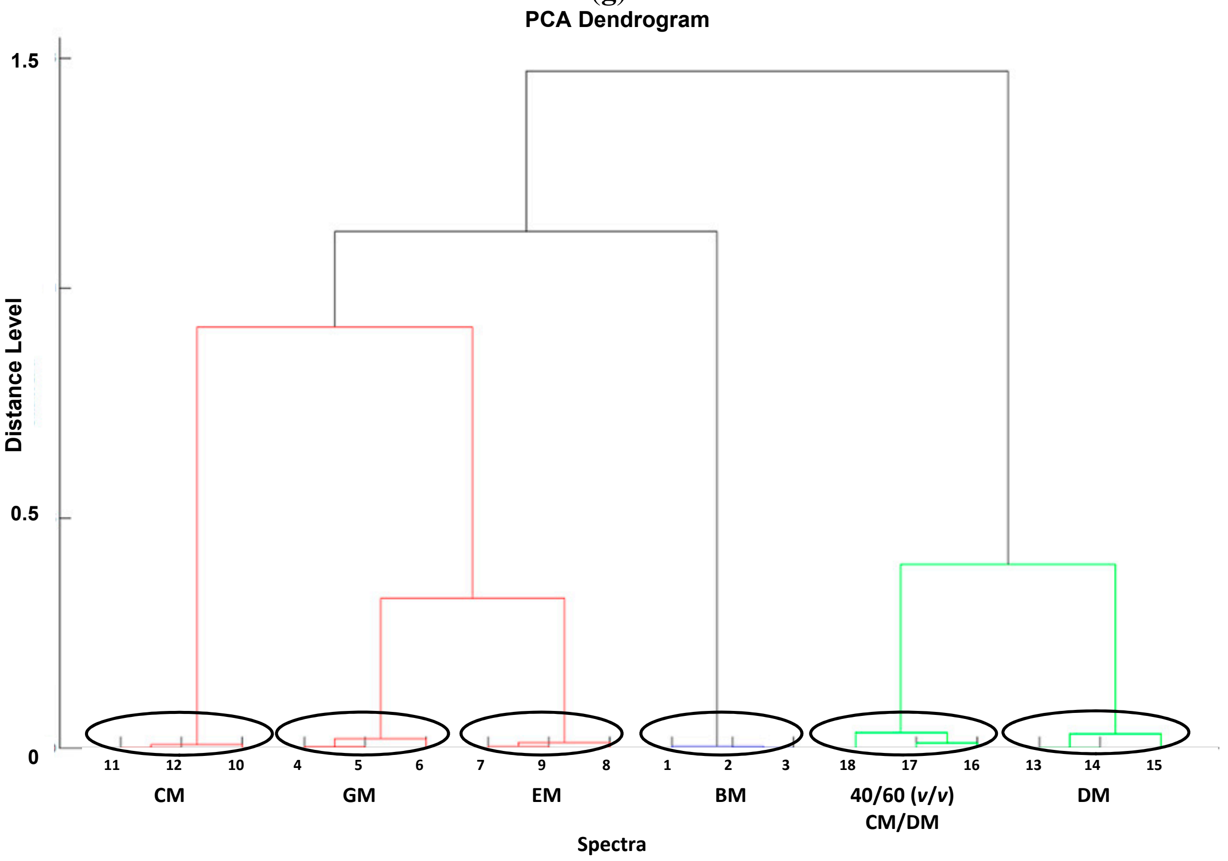

(h)

Figures S2. For the validation of the analytical method, three best reproducible spectra were chosen for each milk species and, a PCA analysis reference was produced (**Panels a**). CM/DM mixtures, in volume ratios 0.3/99.7, 0.4/99.6, 0.7/99.3, 1.5/98.5, 9/91, 15/85, 40/60 were prepared and a blinded investigator analyzed them. **Panels a–h** shows the respective PCA analysis, producing 3D scatter plot images. Figure S2 shows clearly the high performance of the analytical method in the identification of all the adulteration levels (from 0.3% to 40% of CM addition) for all the test samples prepared.

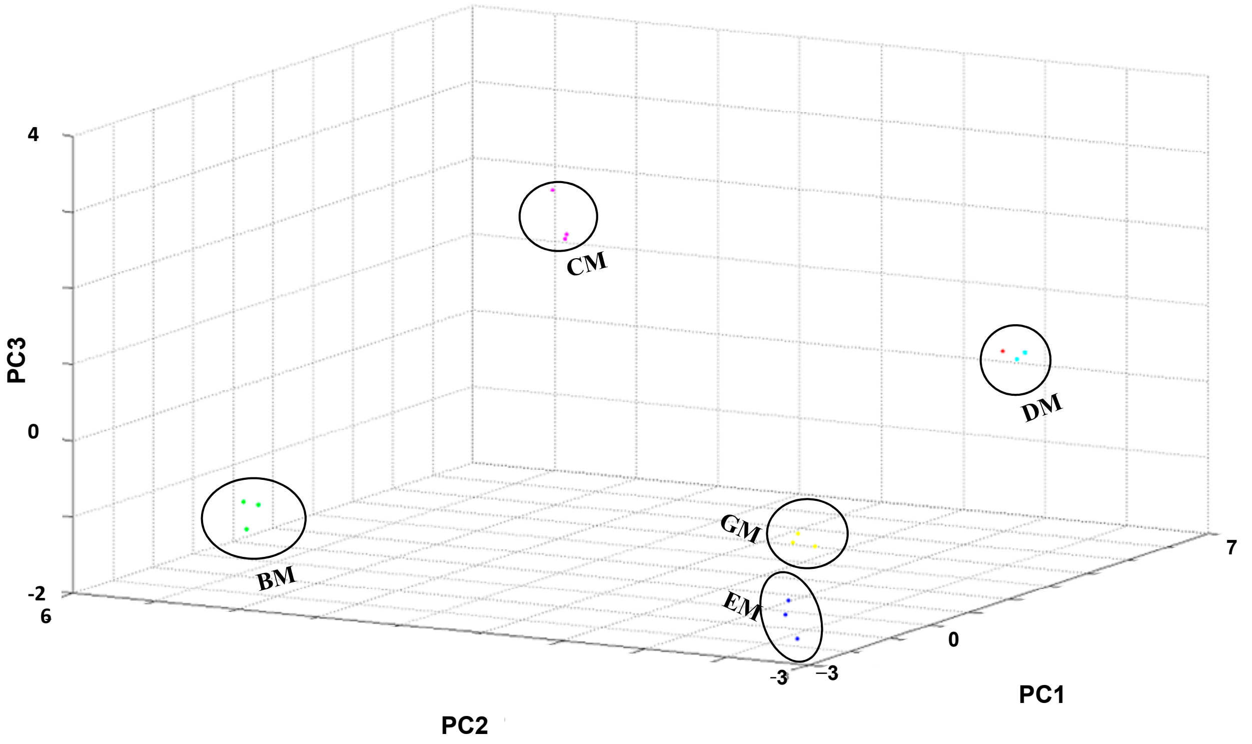

(**a**)

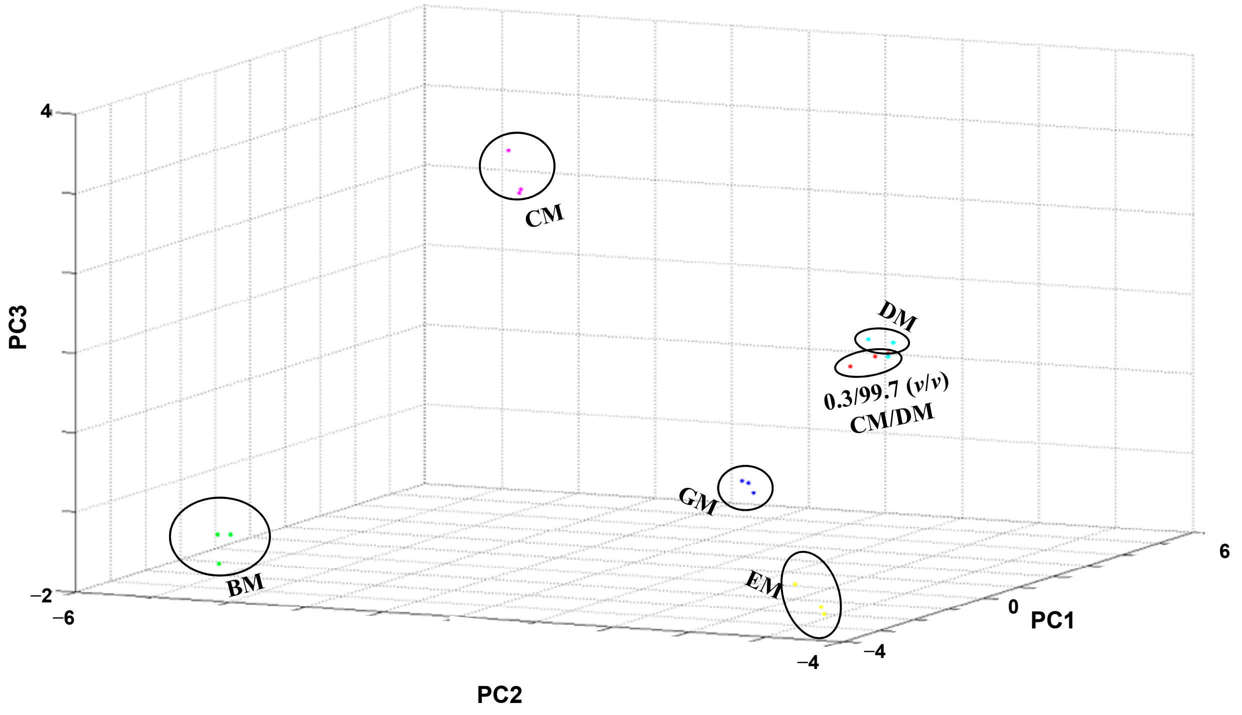

(**b**)

Figure S2. *Cont.*

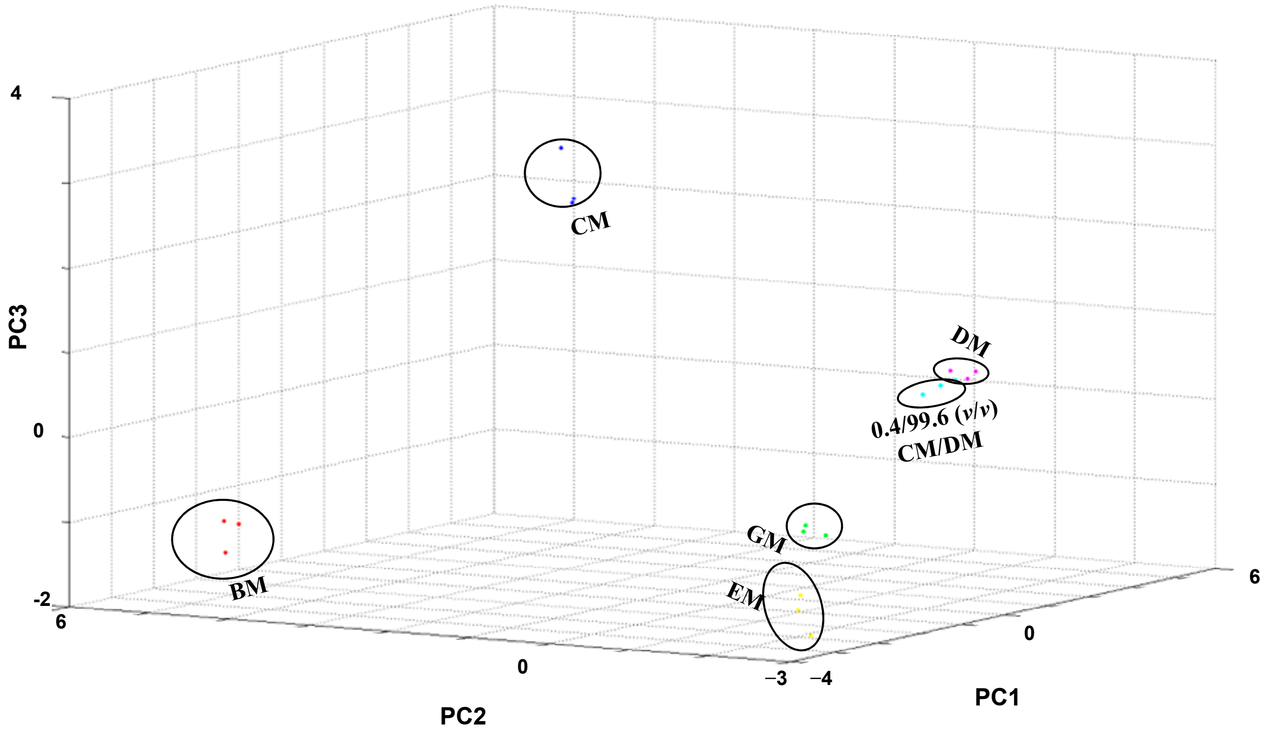

(**c**)

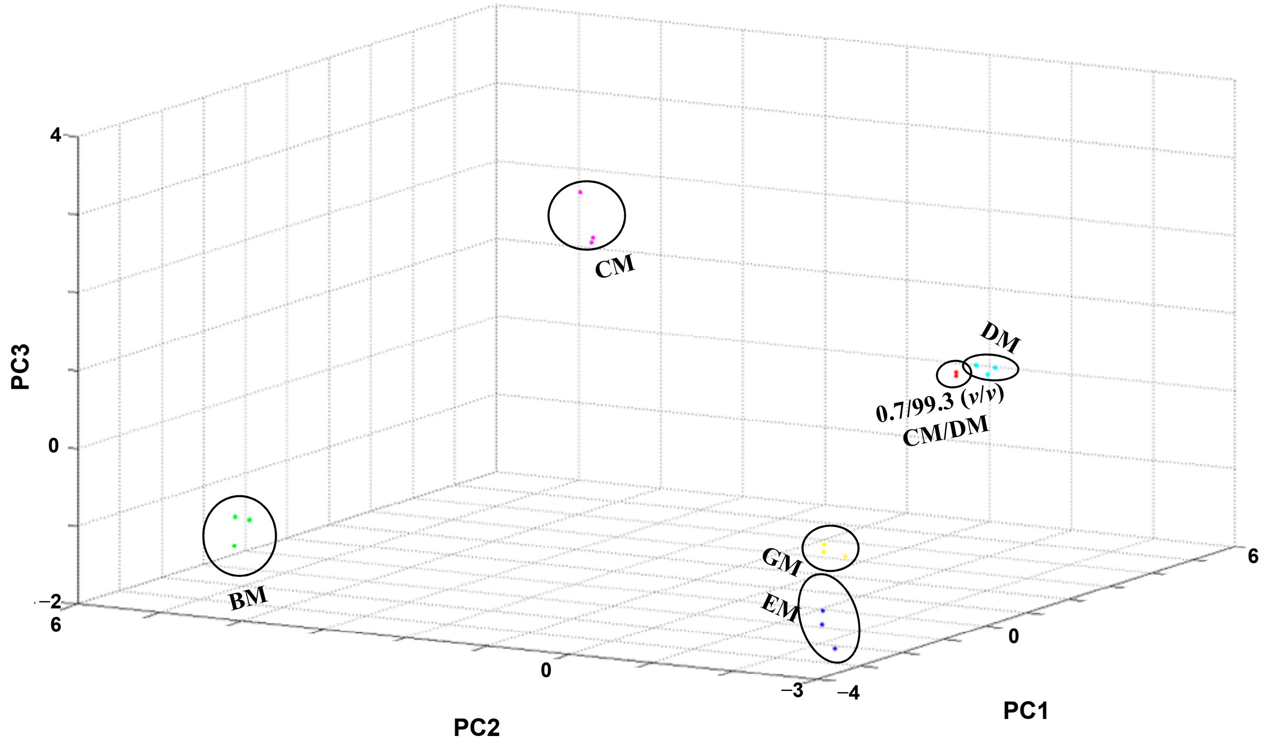

(**d**)

Figure S2. *Cont.*

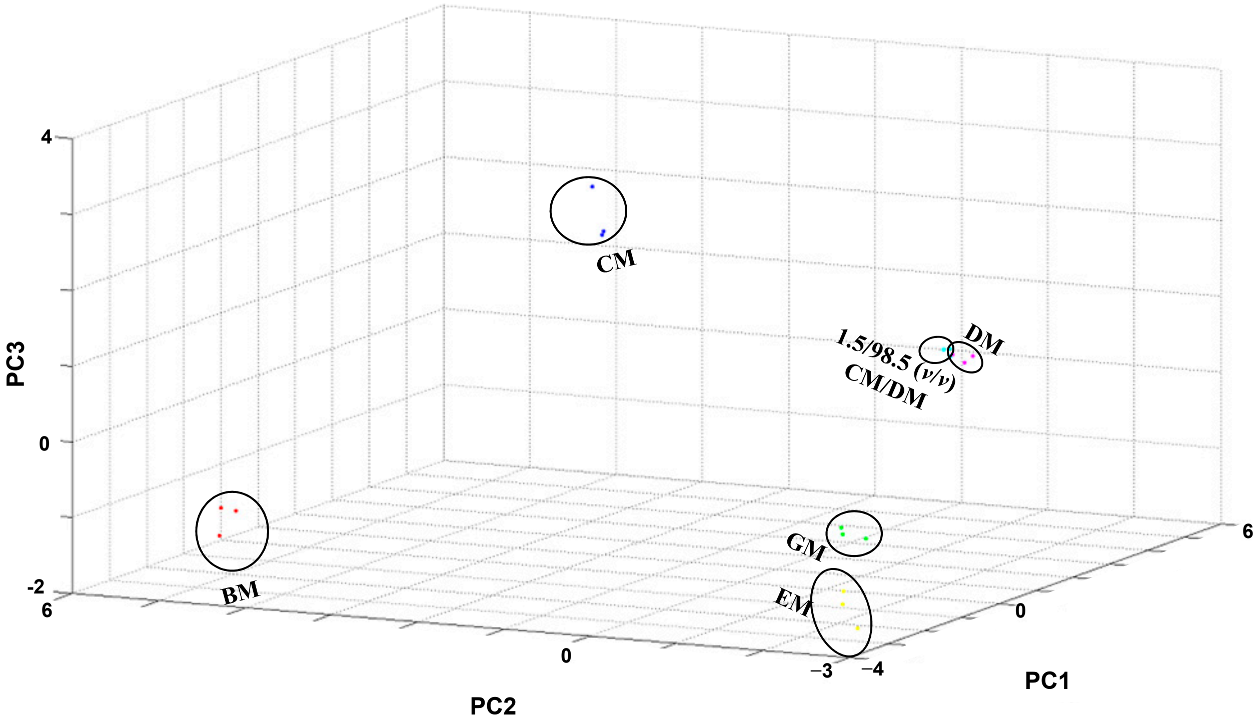

(e)

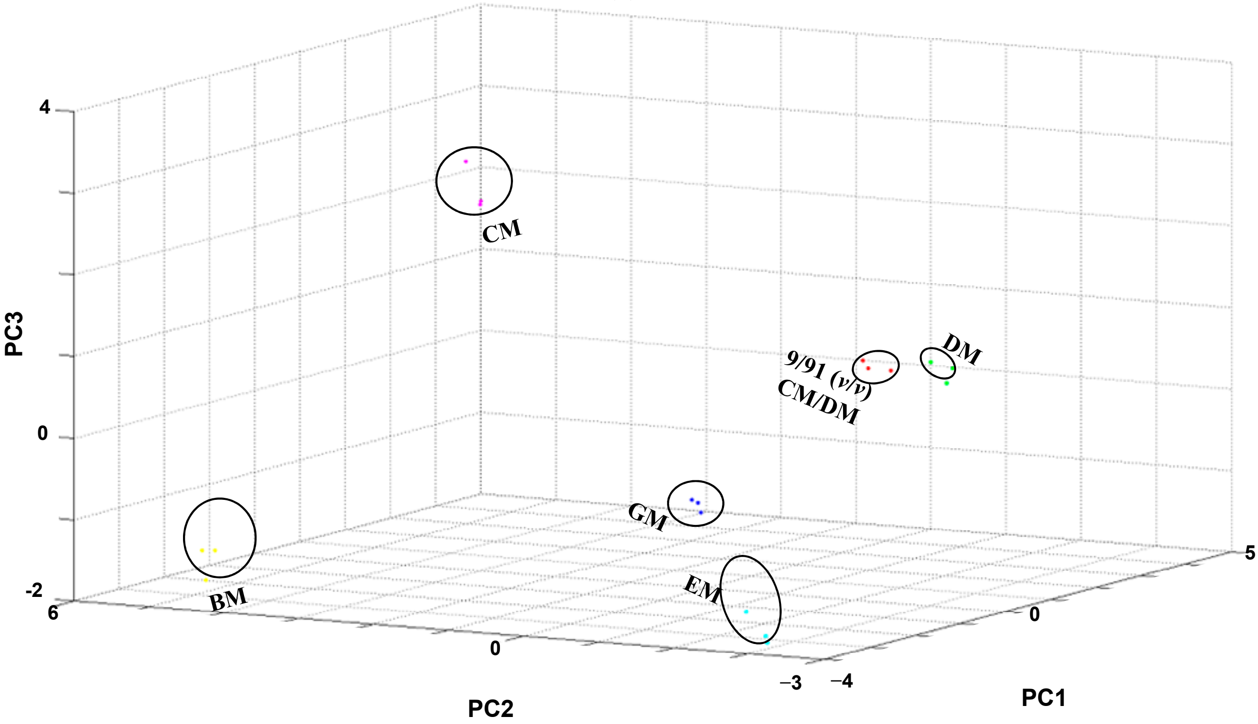

(f)

Figure S2. *Cont.*

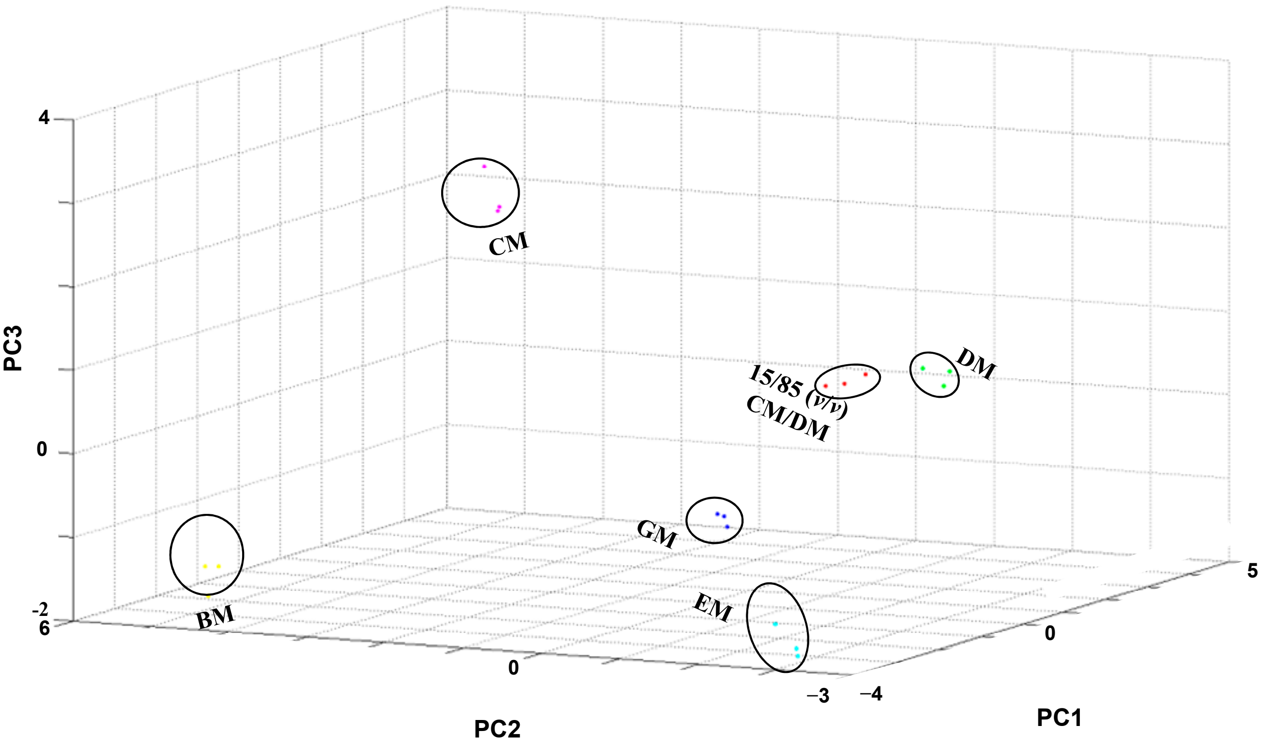

(g)

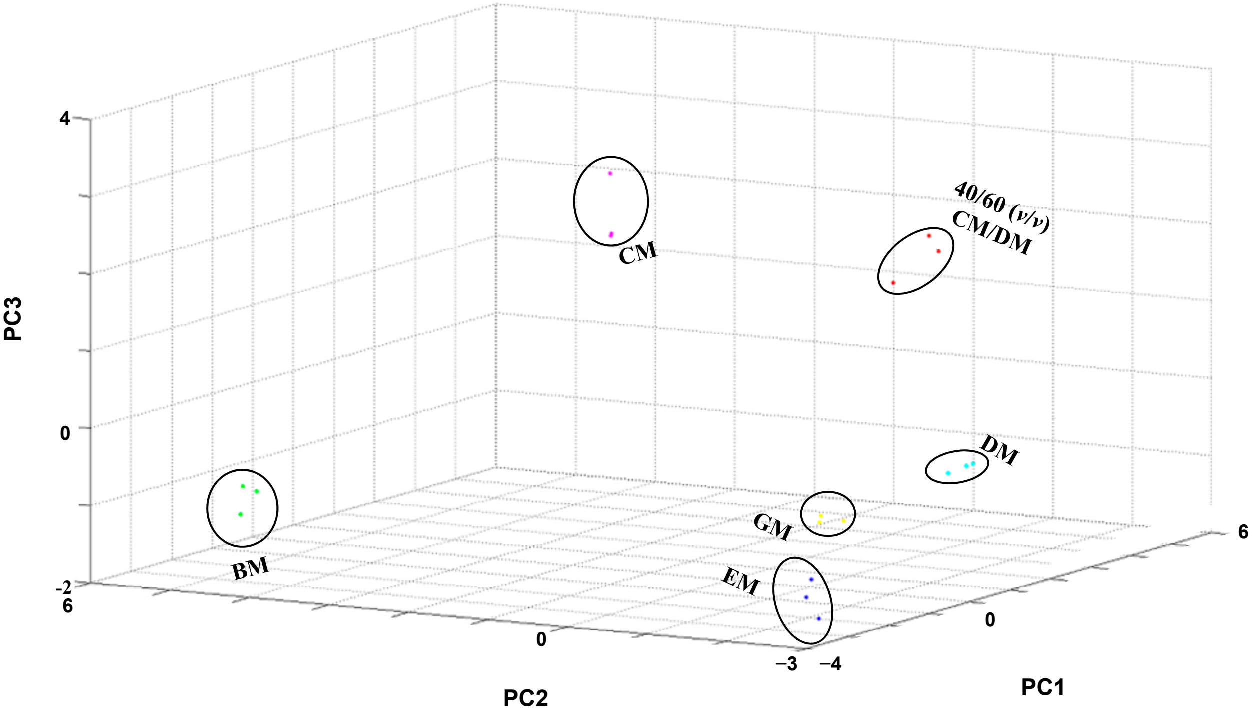

(h)

Figures S3. Three best reproducible spectra were chosen for each level of adulteration, producing a reference dendrogram (**Panel a**). CM/DM mixtures, in volume ratios 0.3/99.7, 0.4/99.6, 0.7/99.3, 1.5/98.5, 9/91, 15/85, 40/60 were prepared and a blinded investigator analyzed them (**Panels b–h**). The figure shows a hierarchical clustering analysis, producing dendrograms. The figure shows the low performance of the analytical method in the quantitative analysis.

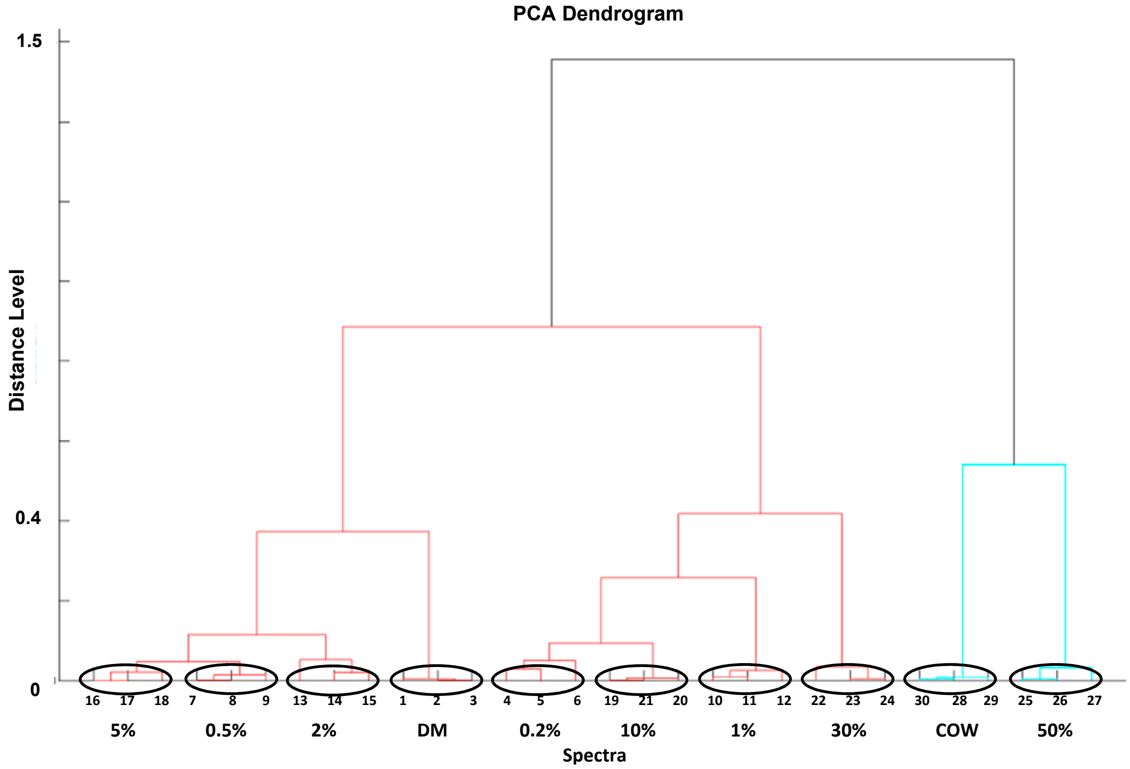

(**a**)

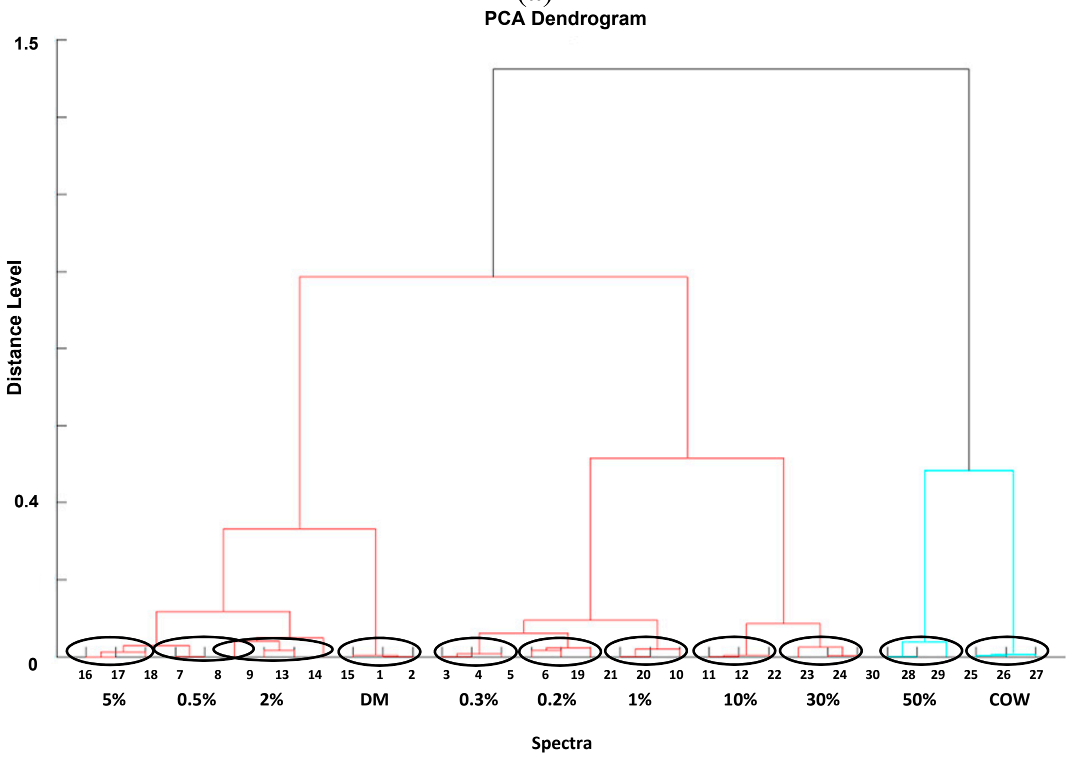

(**b**)

Figure S3. *Cont.*

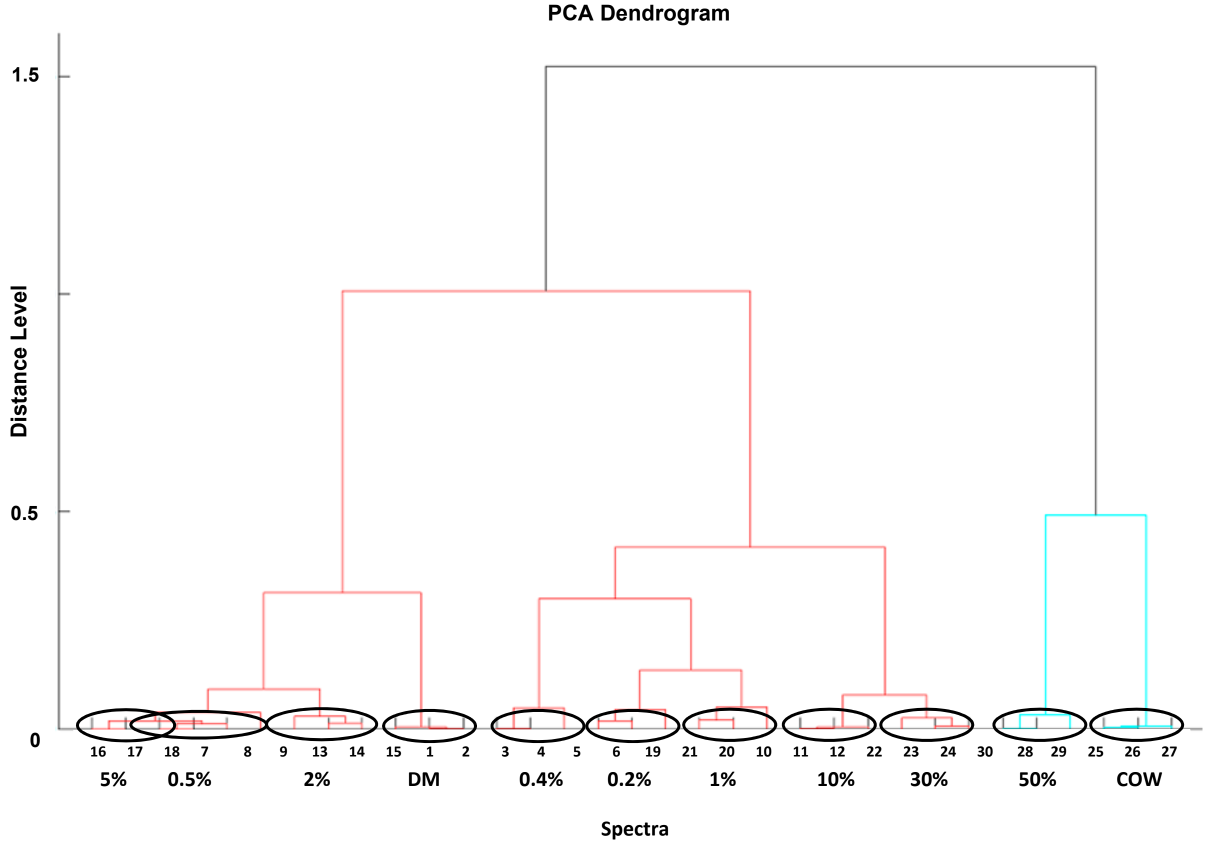

(c)

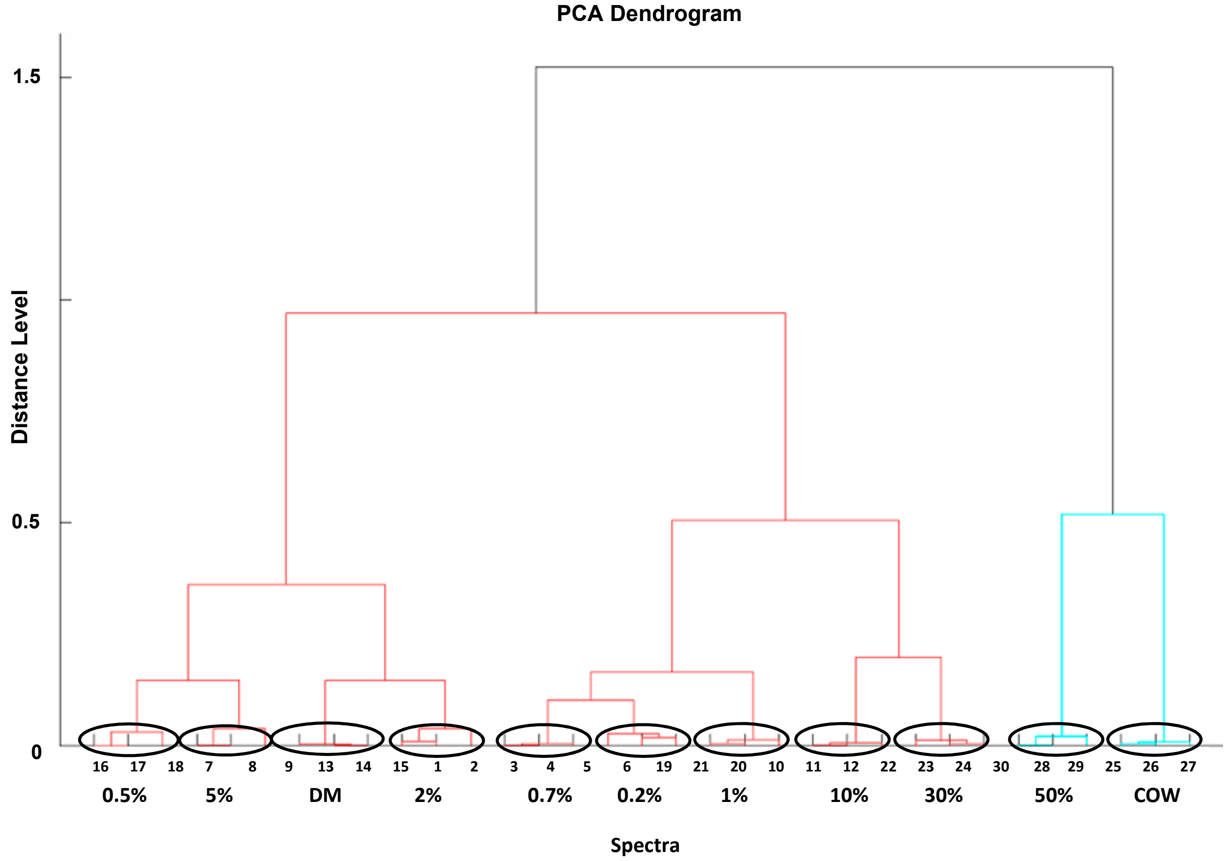

(d)

Figure S3. *Cont.*

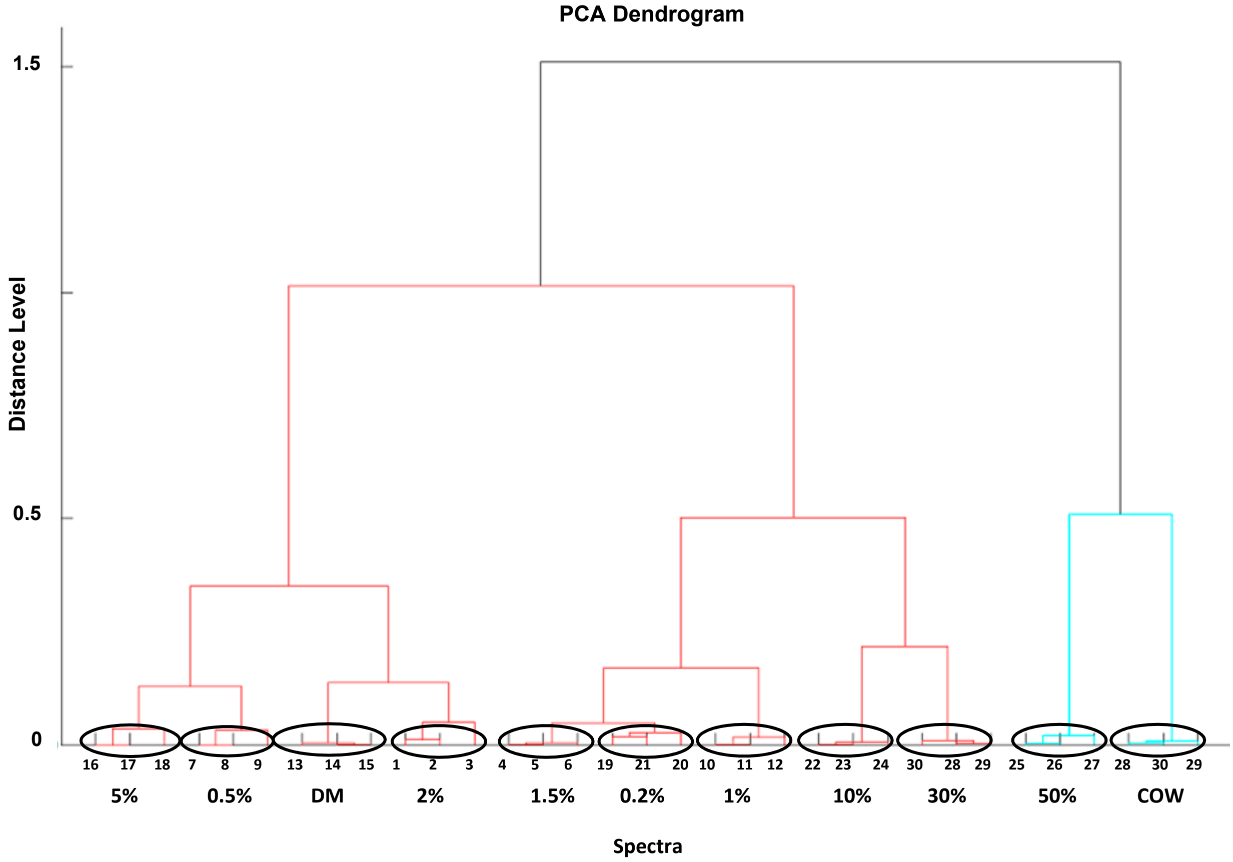

(e)

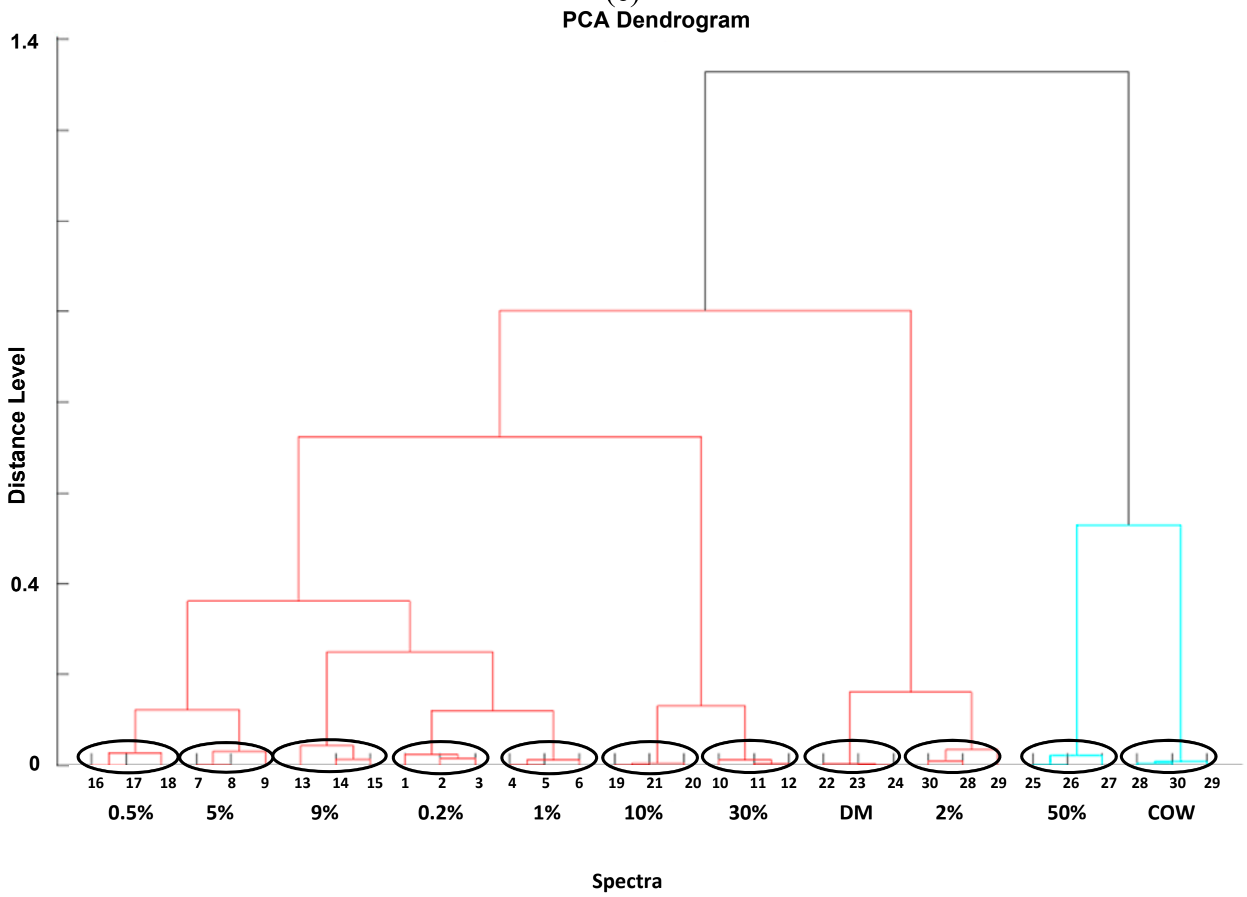

(f)

Figure S3. *Cont.*

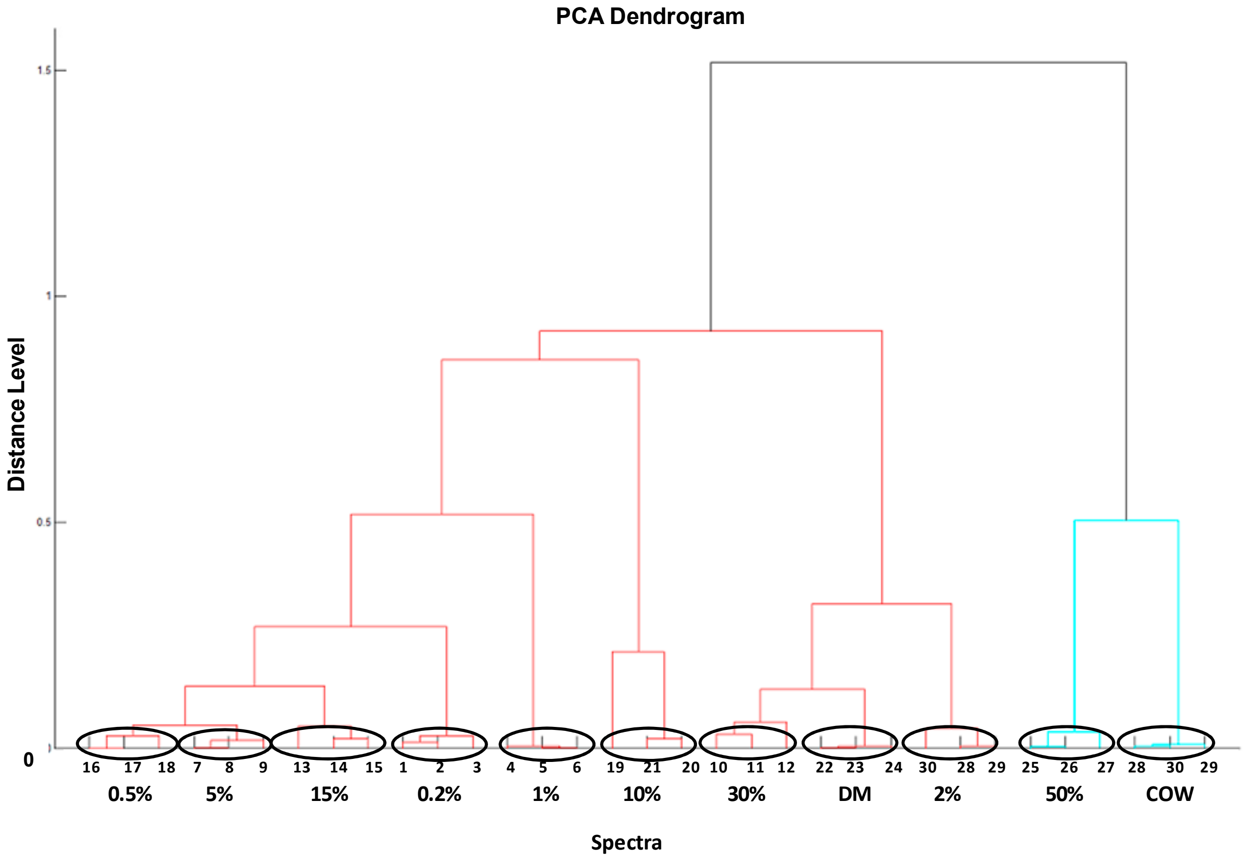

(g)

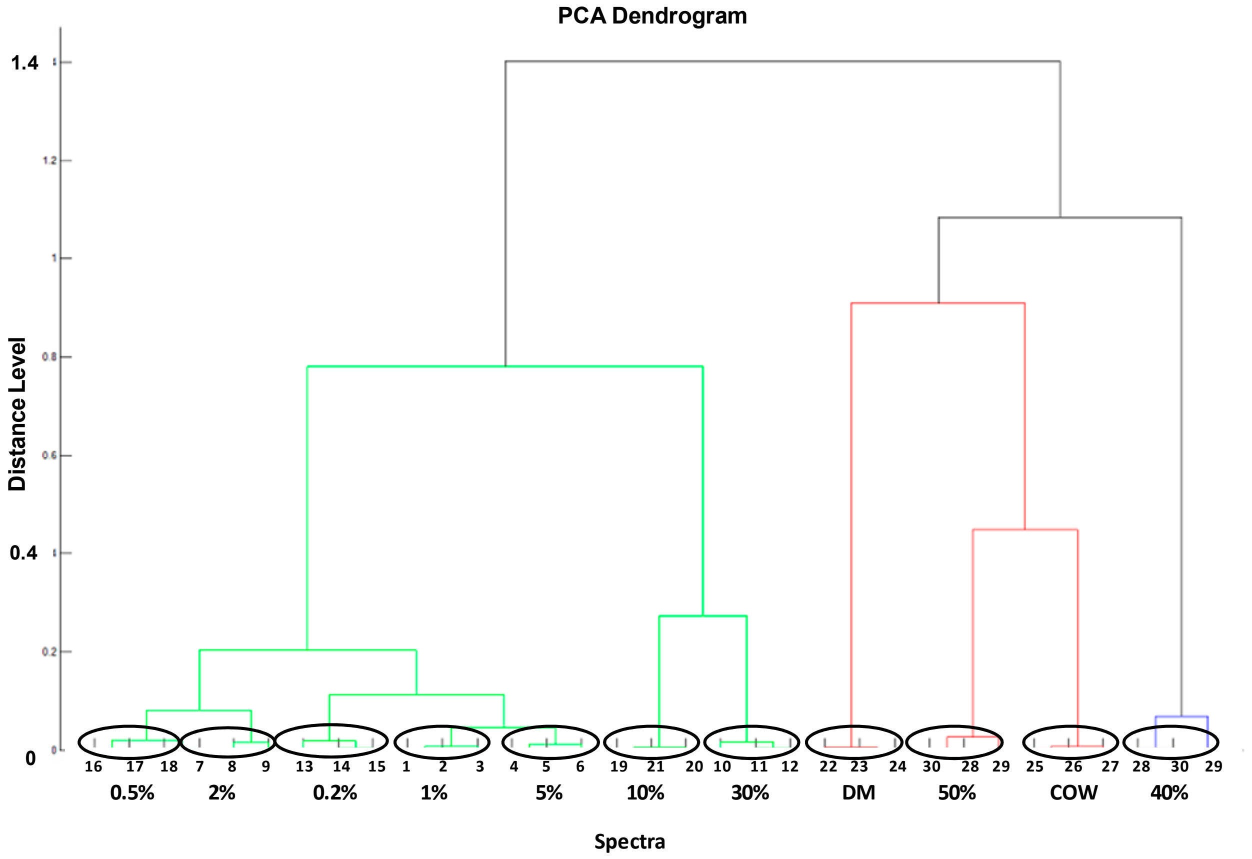

(h)

Figures S4. Hierarchical clustering and dendrograms and PCA 3D scatter plots of the first three principle components for the three mixtures in volume ratios 0.2/99.8, 10/90, and 50/50 of the simulated adulteration of the pasteurized DM and GM by CM (**Panels a–p**). All milk adulterations grouped inside the correct clade and cluster.

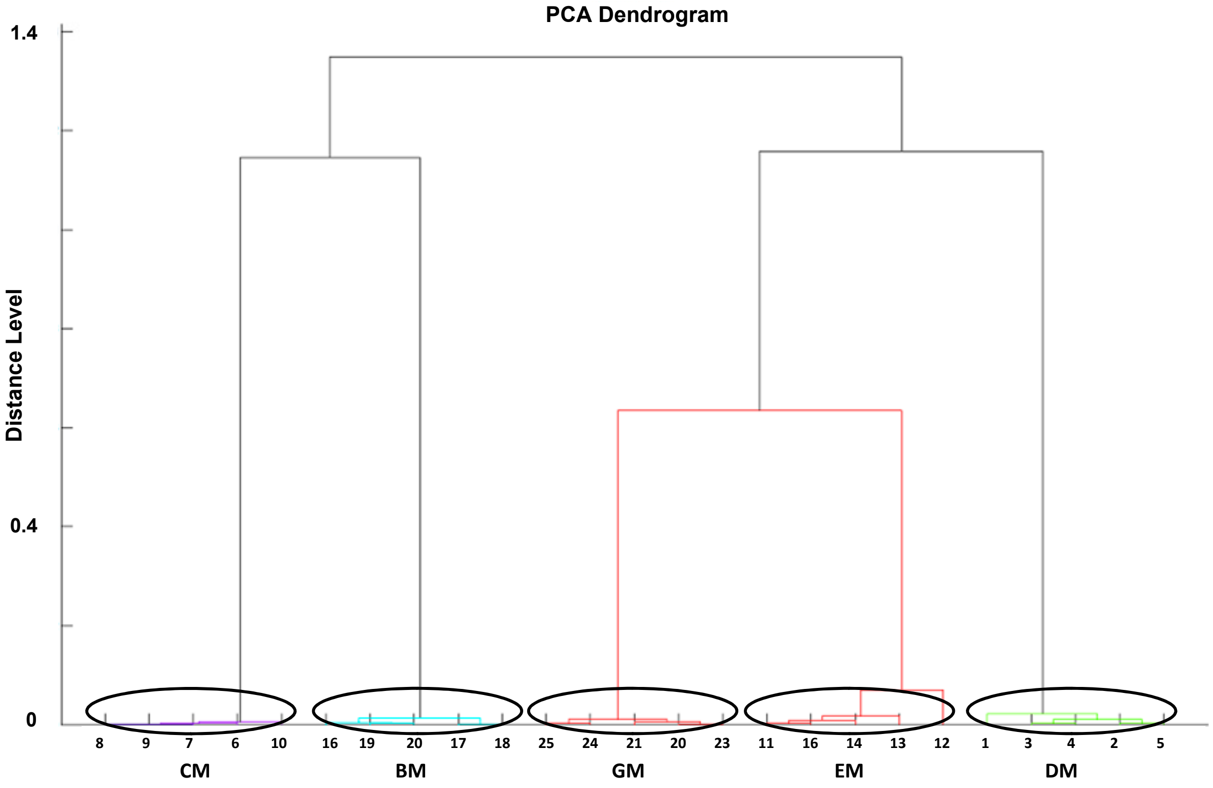

(**a**)

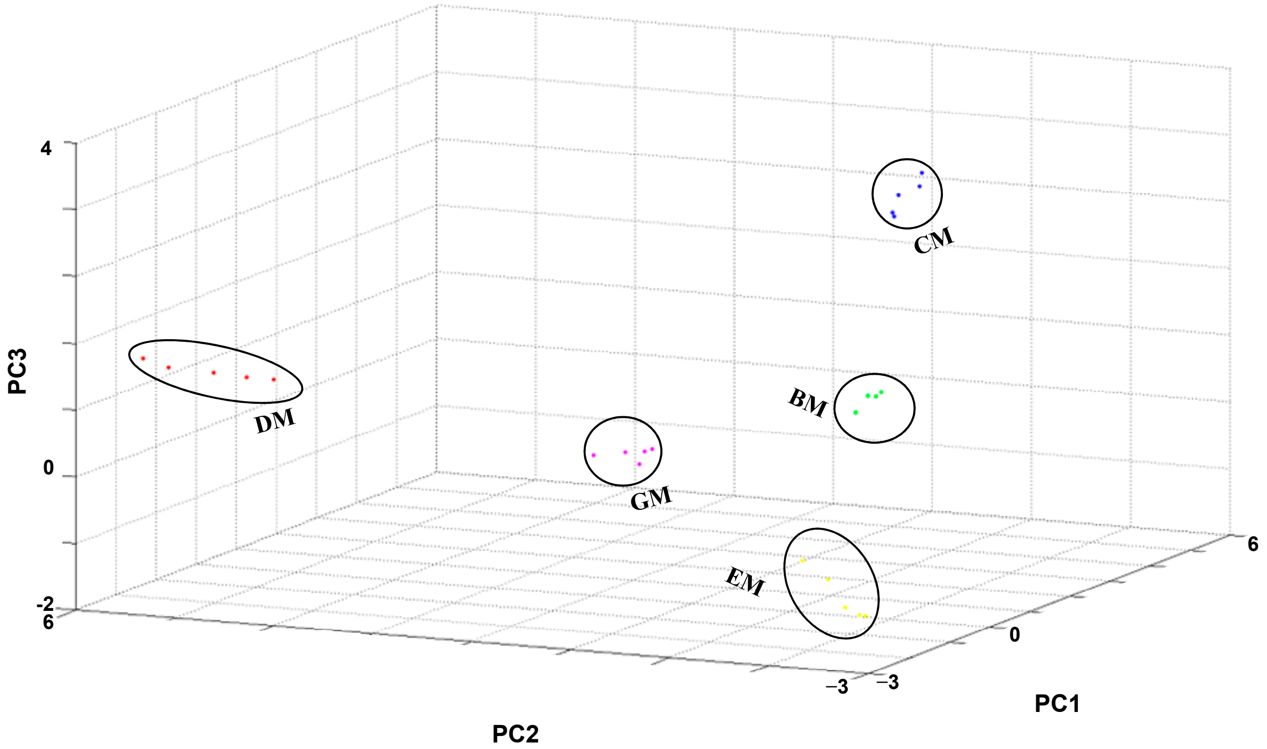

(**b**)

Figure S4. *Cont.*

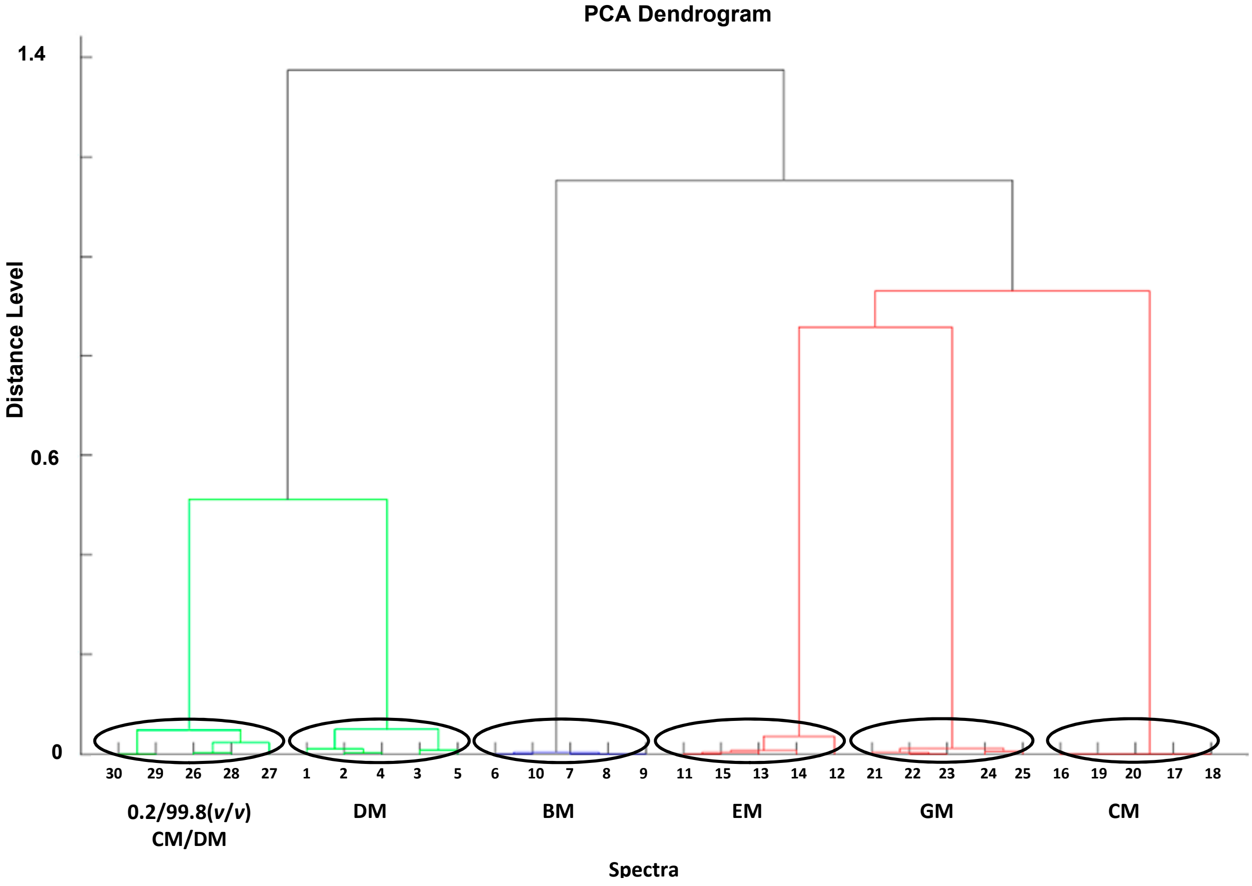

(c)

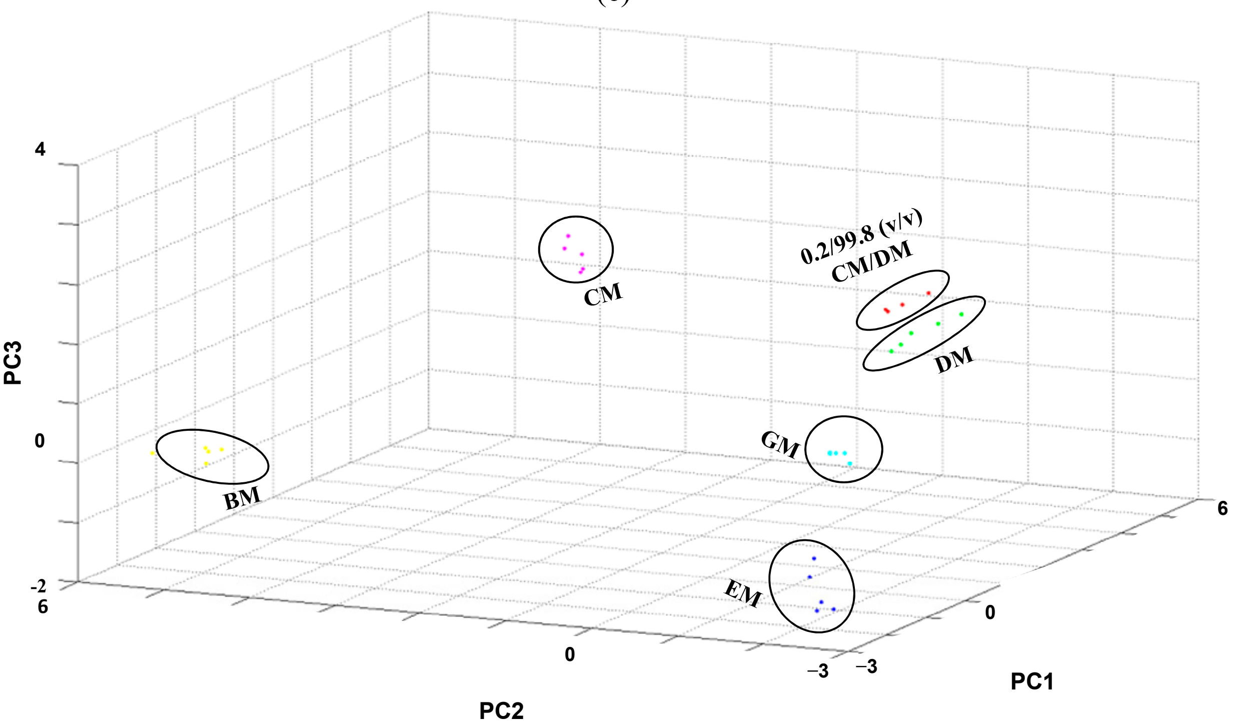

(d)

Figure S4. *Cont.*

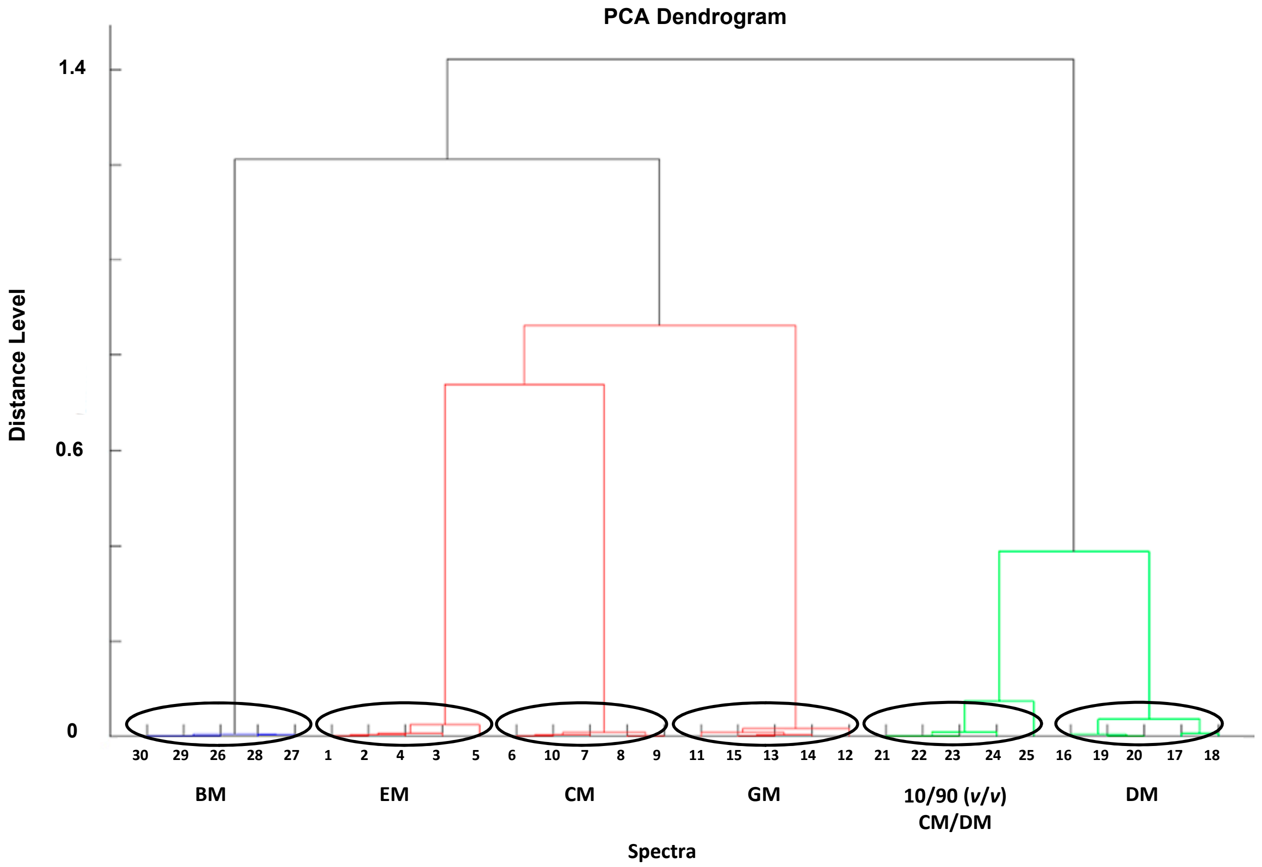

(e)

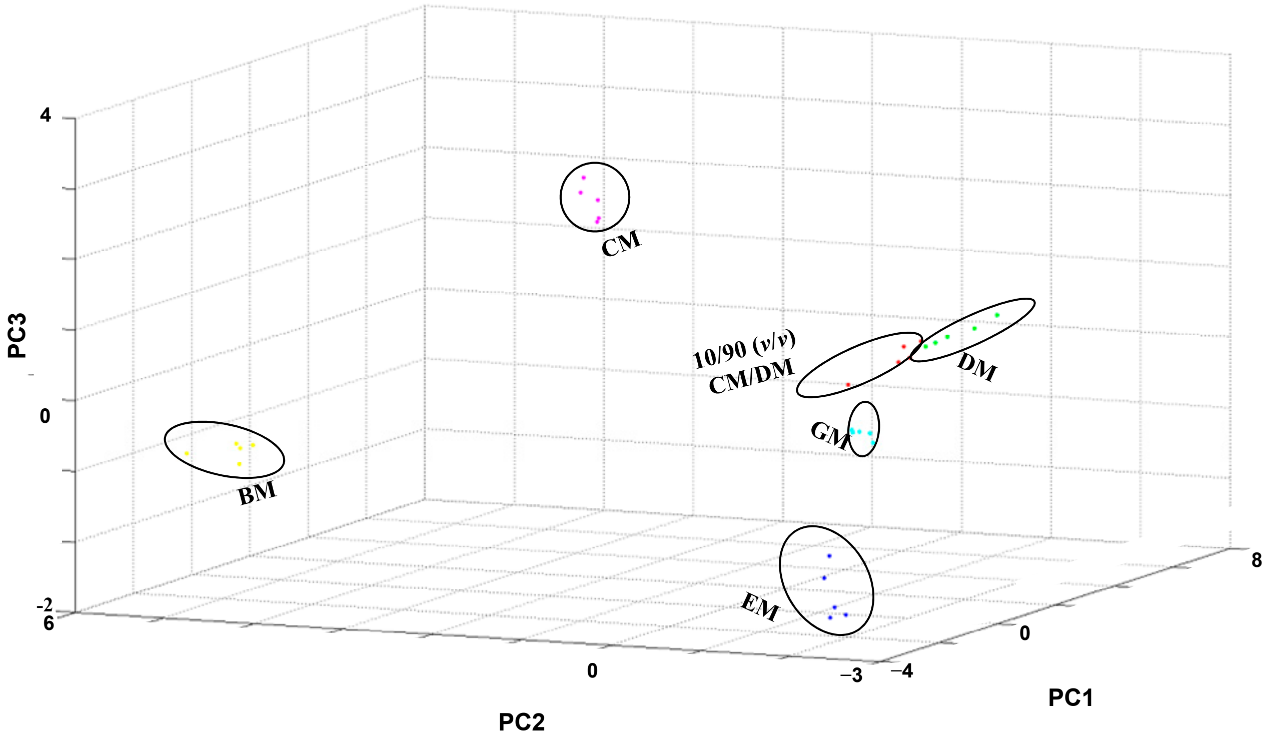

(f)

Figure S4. *Cont.*

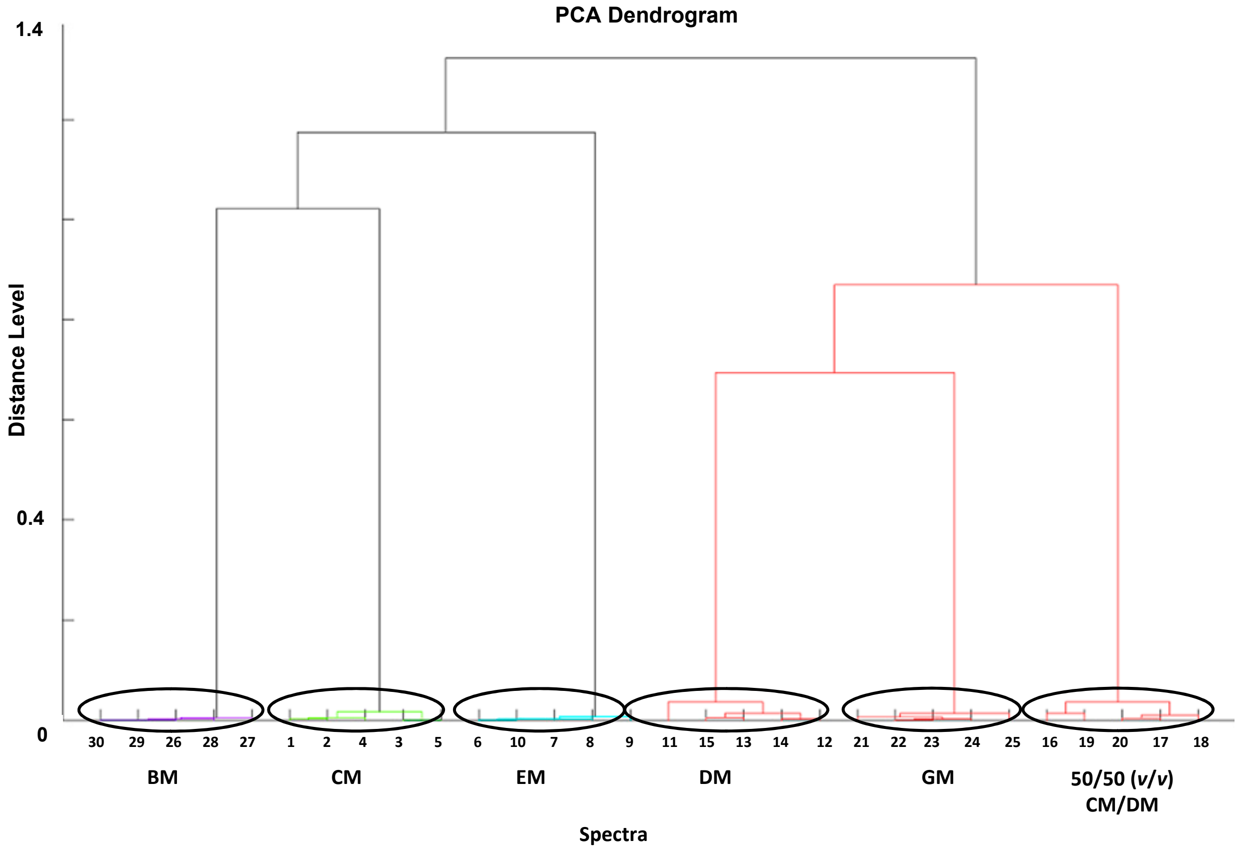

(g)

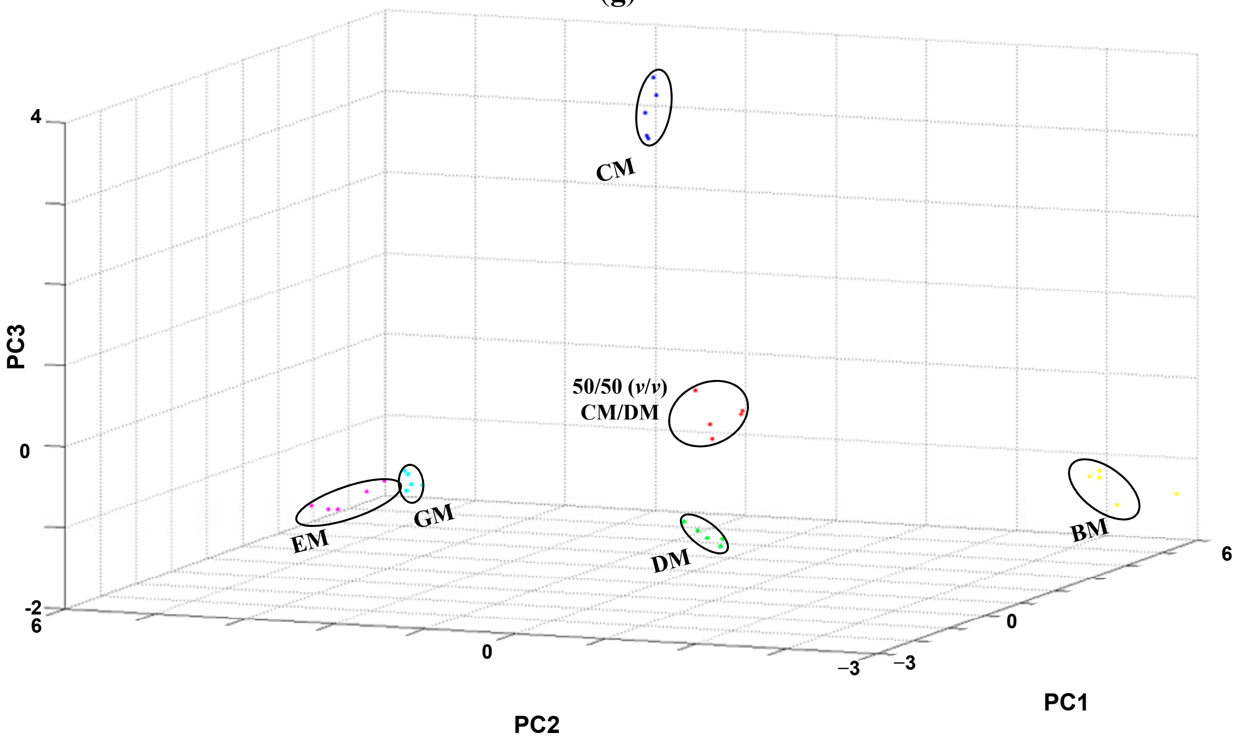

(h)

Figure S4. *Cont.*

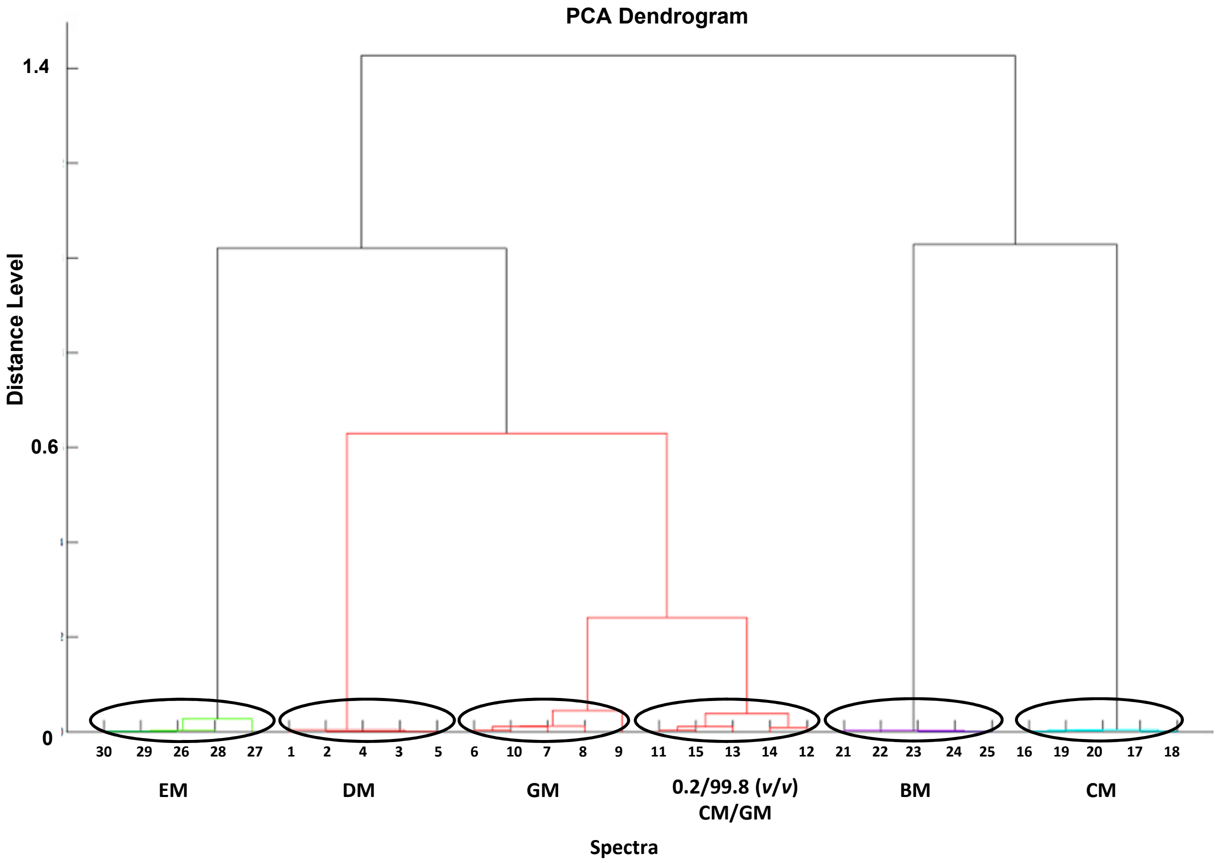

(i)

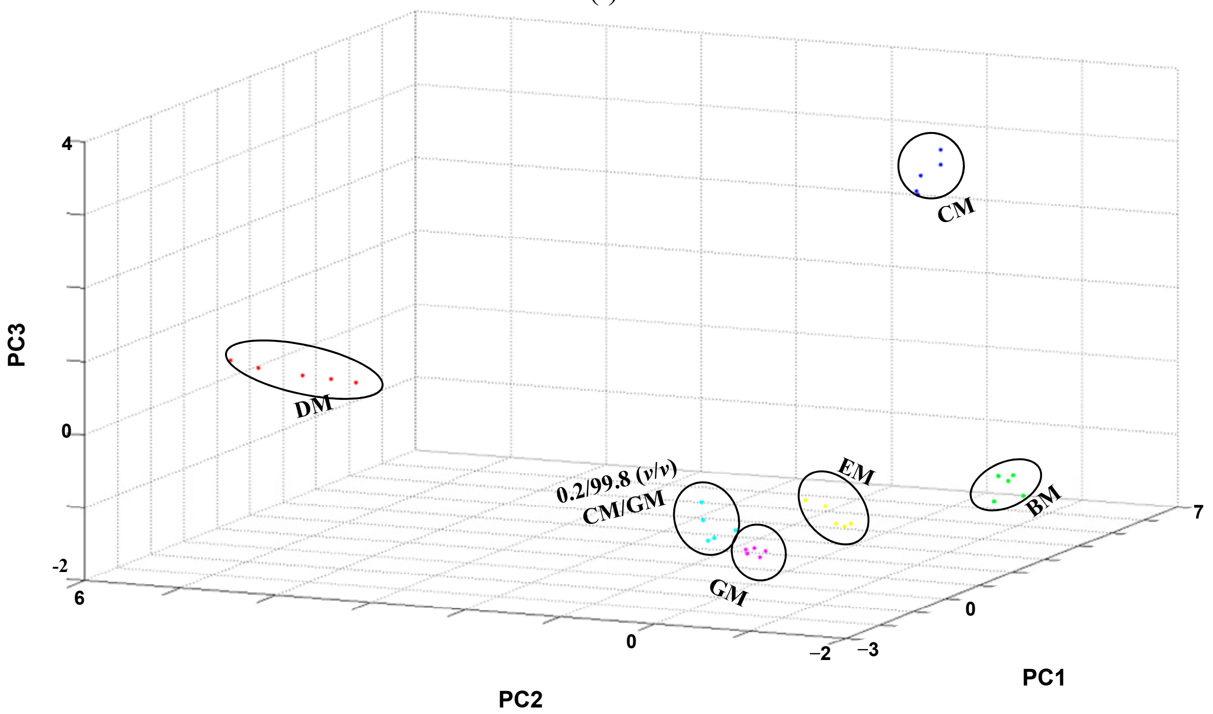

(l)

Figure S4. *Cont.*

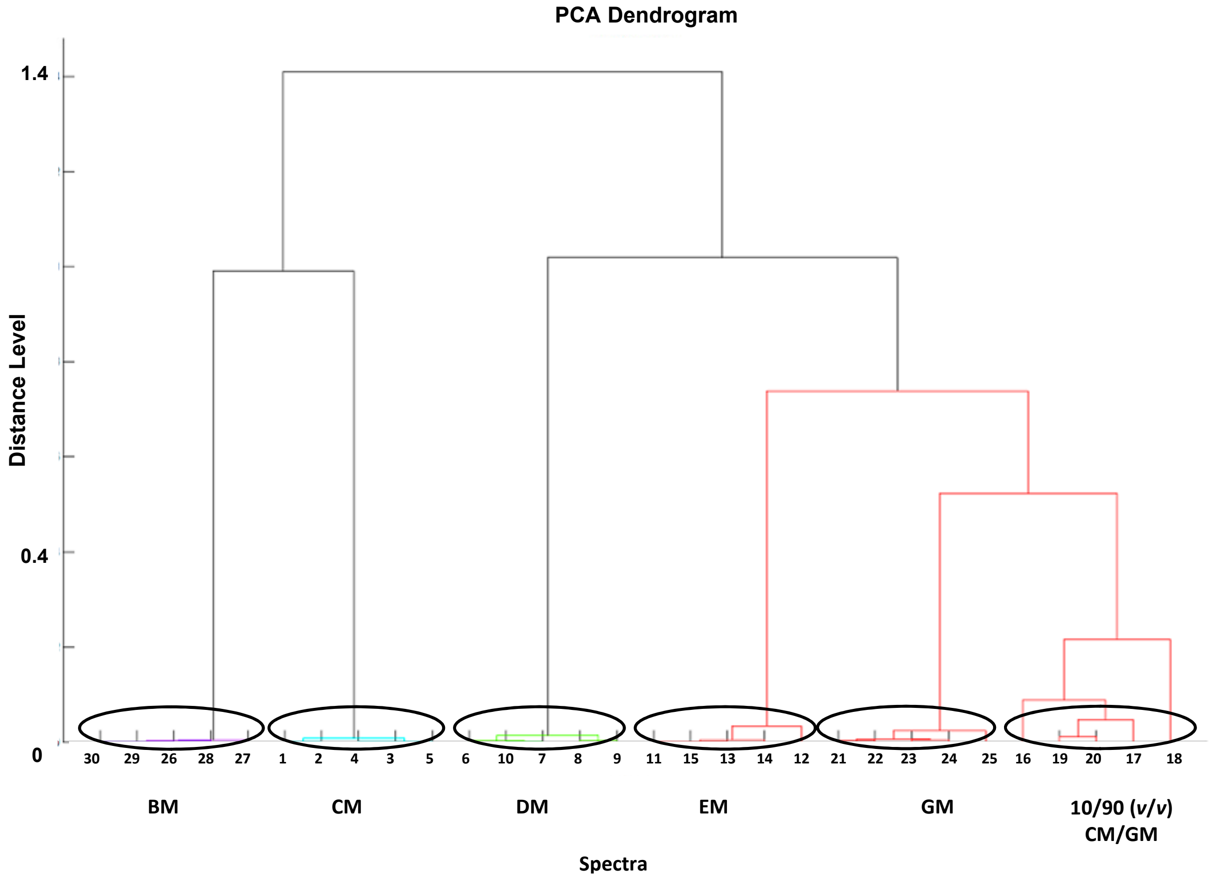

(m)

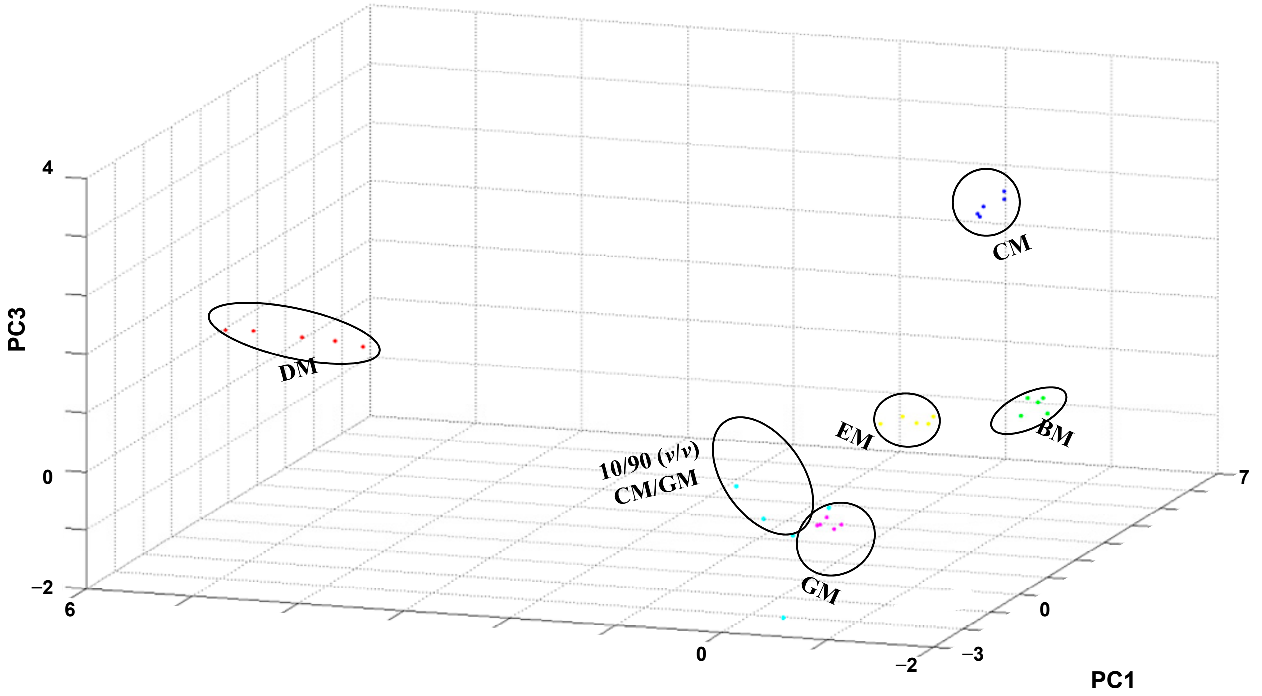

(n)

Figure S4. *Cont.*

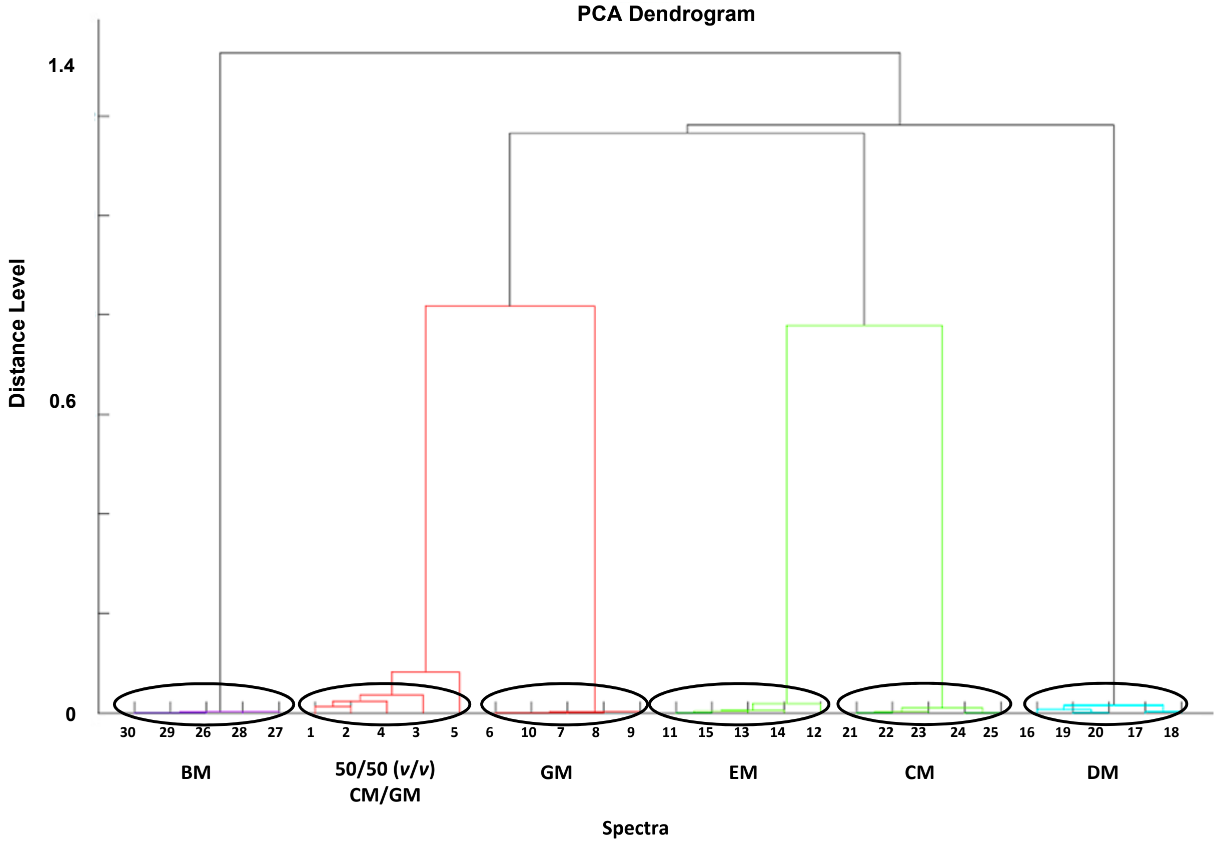

(**o**)

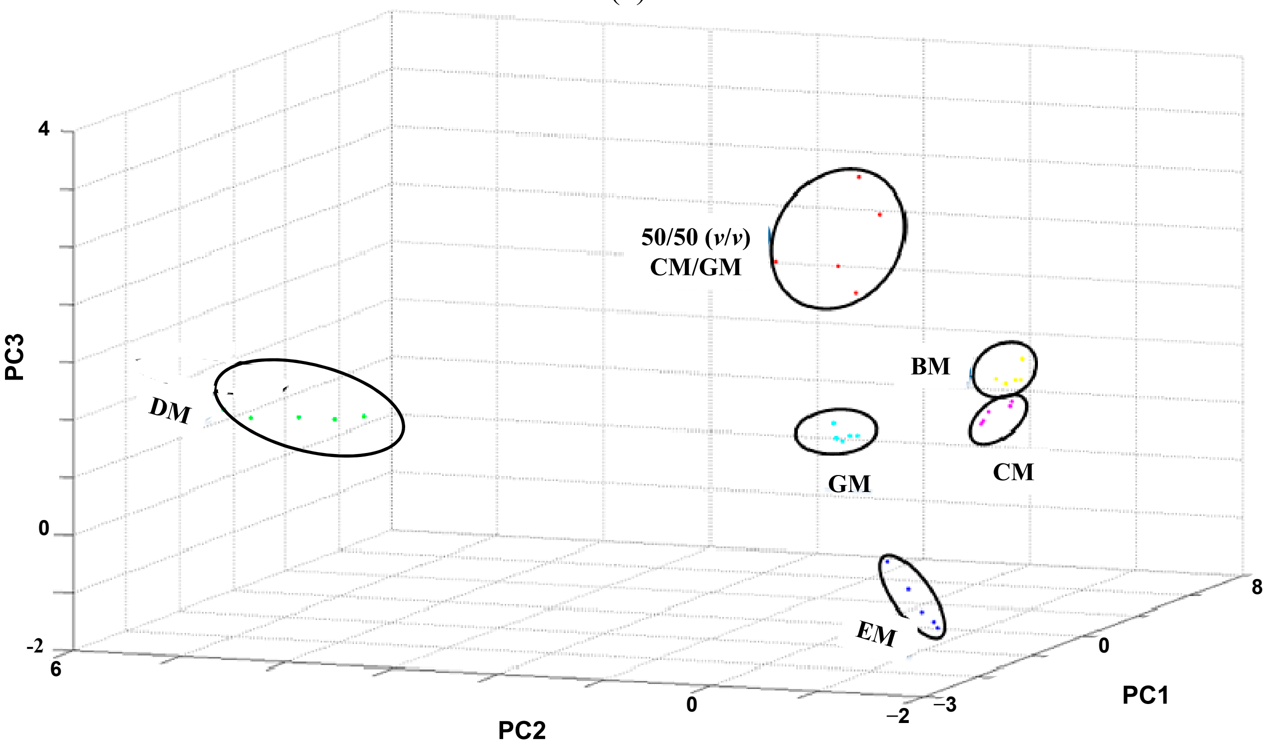

(**p**)

Figures S5. Hierarchical clustering and dendrograms and PCA 3D scatter plots of the first three principle components for the three mixtures in volume ratios 0.2/99.8, 10/90, and 50/50 of the simulated adulteration of the UHT DM and GM by CM. All milk adulterations grouped inside the correct clade and cluster (**Panel a–n)**; **Panel o** illustrates the comparison between DM and pasteurized and UHT DM mass spectra. The circle shows the modulation of mass peaks between spectra; **Panel p** illustrates the comparison between GM and pasteurized and UHT GM mass spectra. The circle and the arrows shows the modulation of mass peaks between spectra.

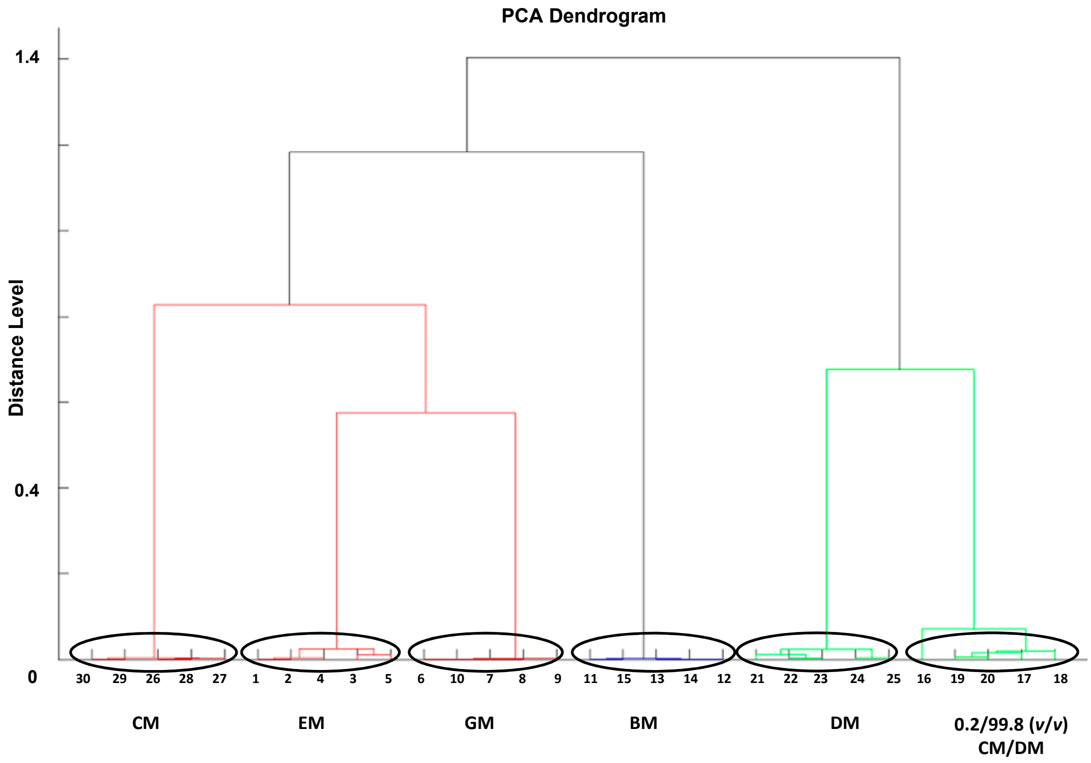

(**a**)

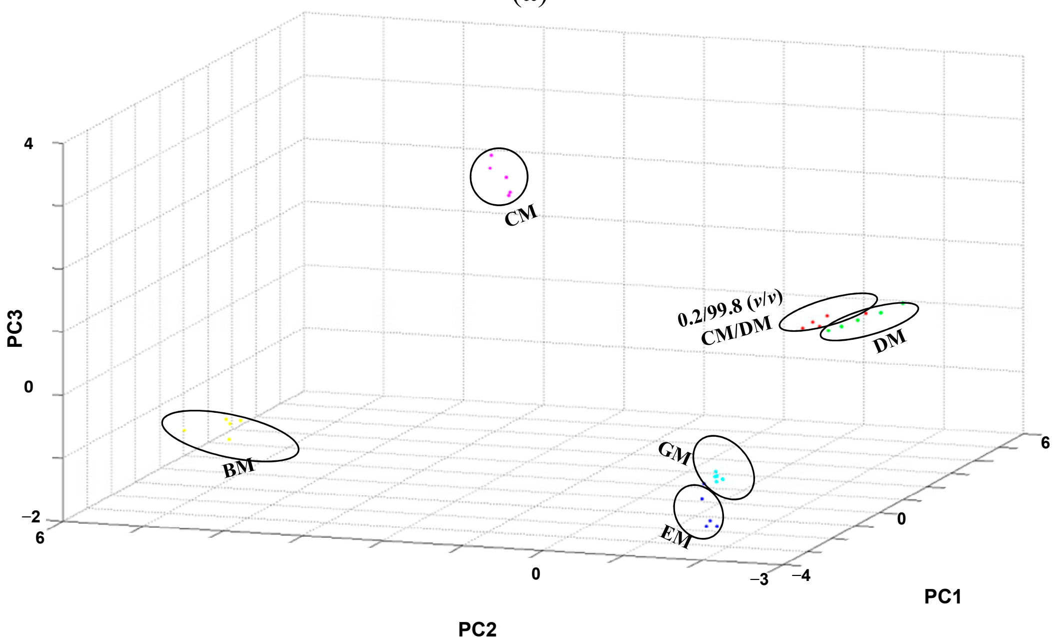

(**b**)

Figure S5. *Cont.*

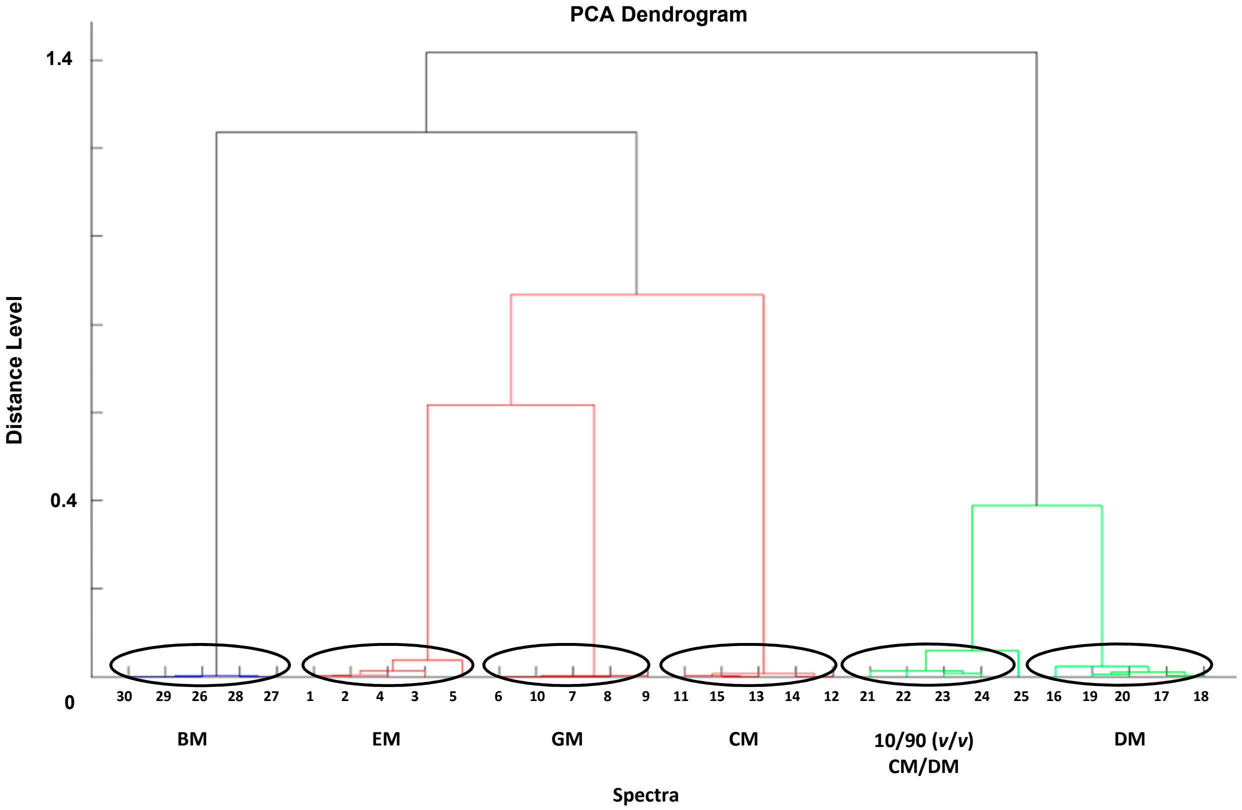

(c)

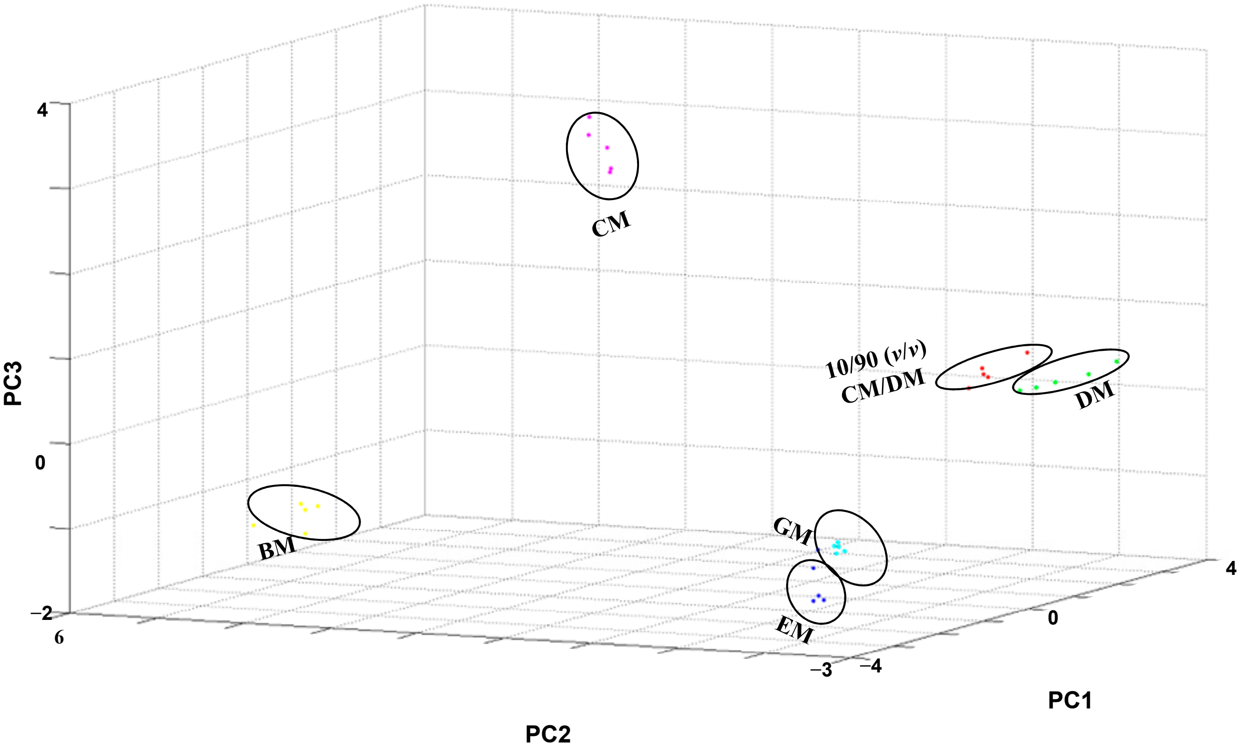

(d)

Figure S5. *Cont.*

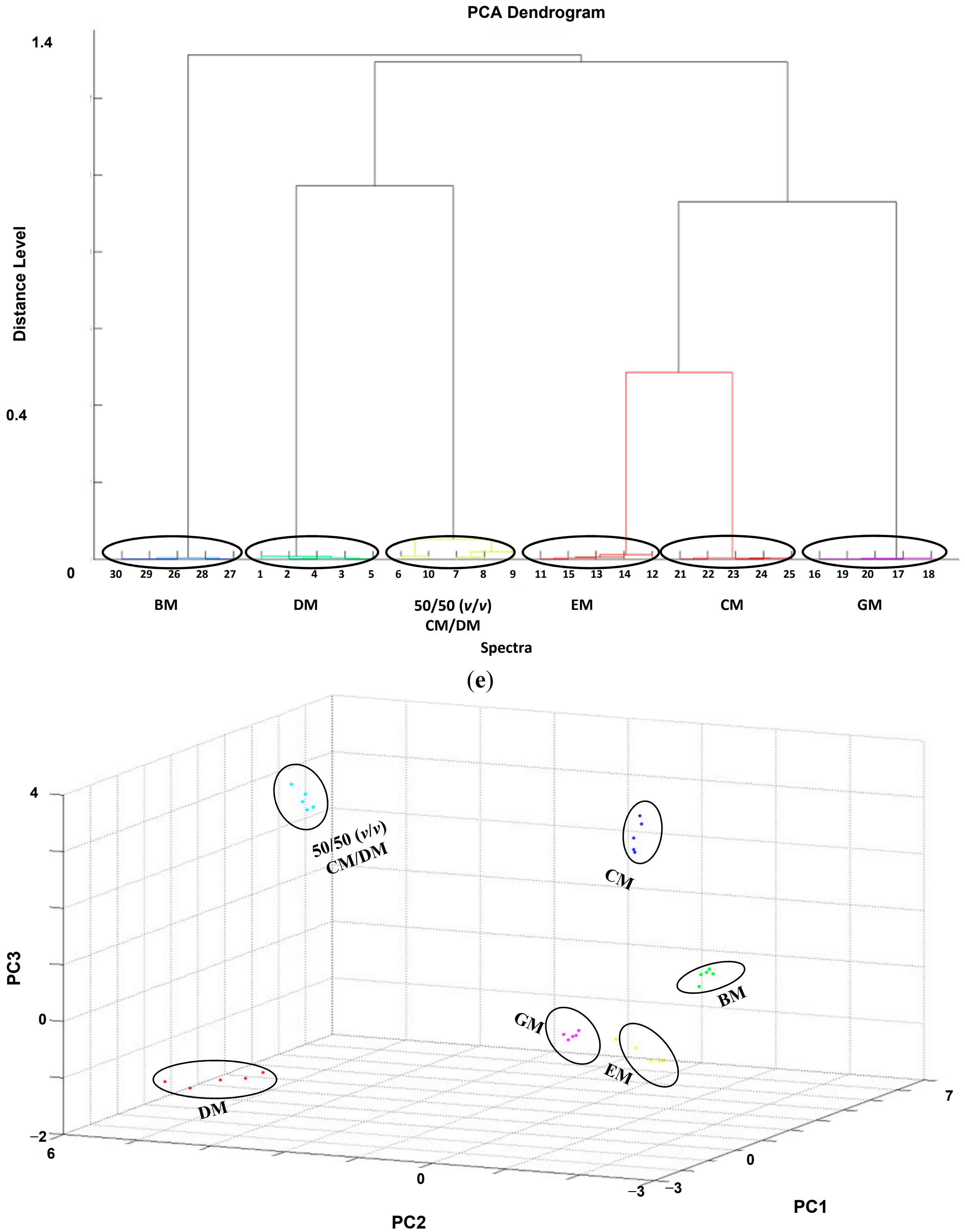

(f)

Figure S5. *Cont.*

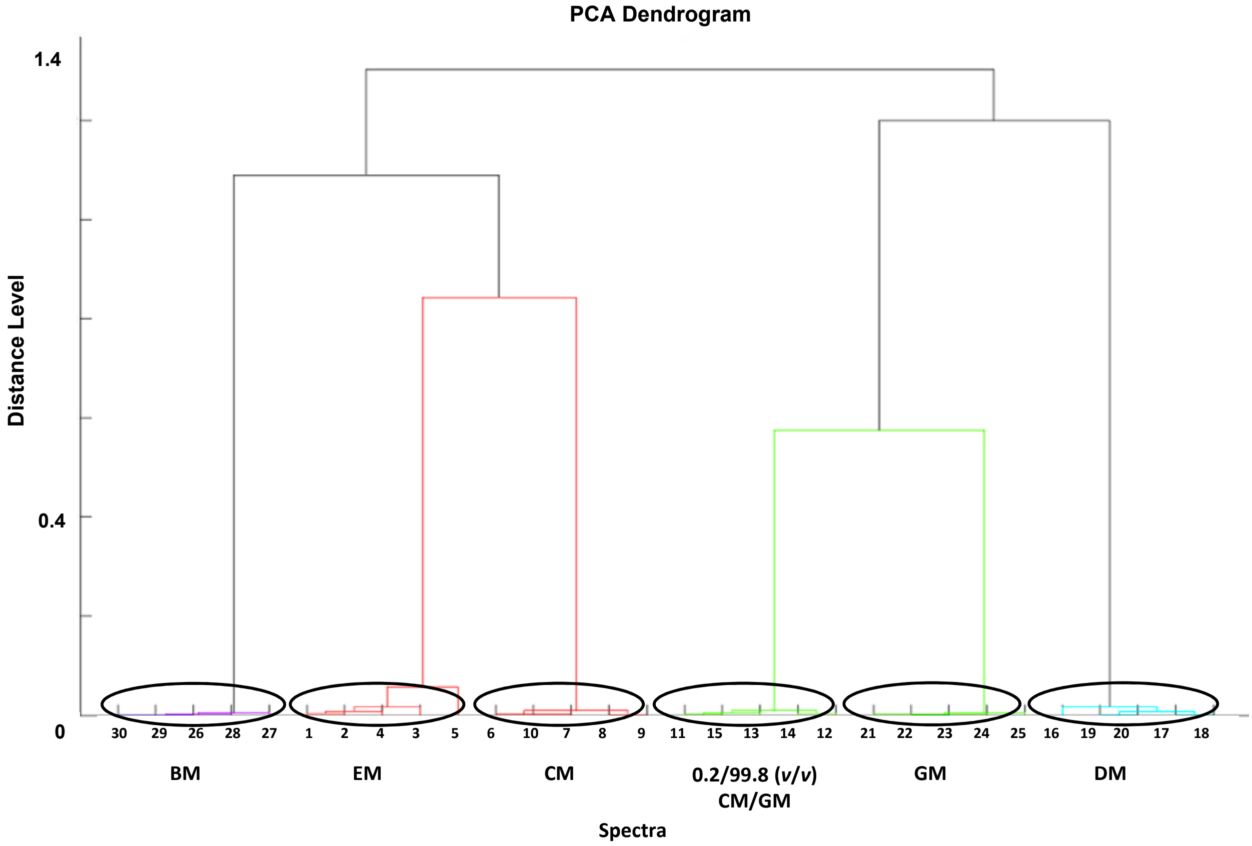

(g)

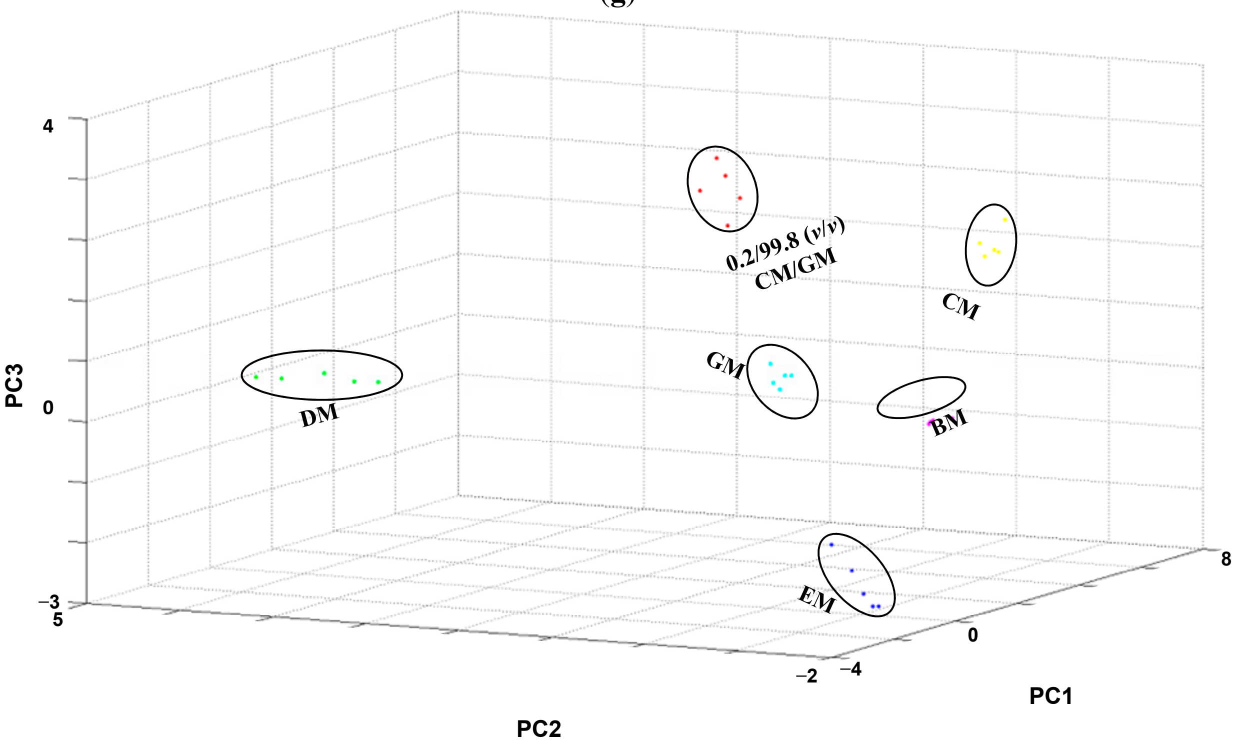

(h)

Figure S5. *Cont.*

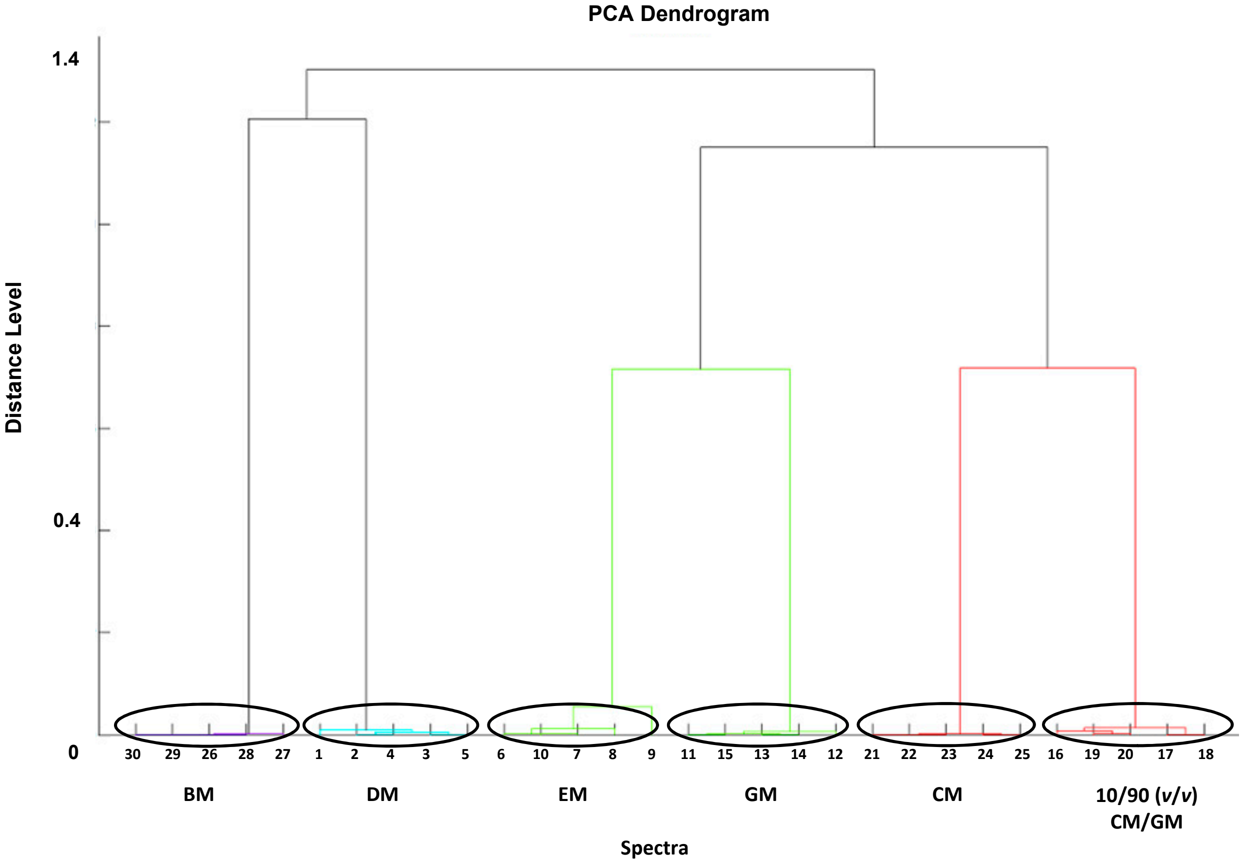

(i)

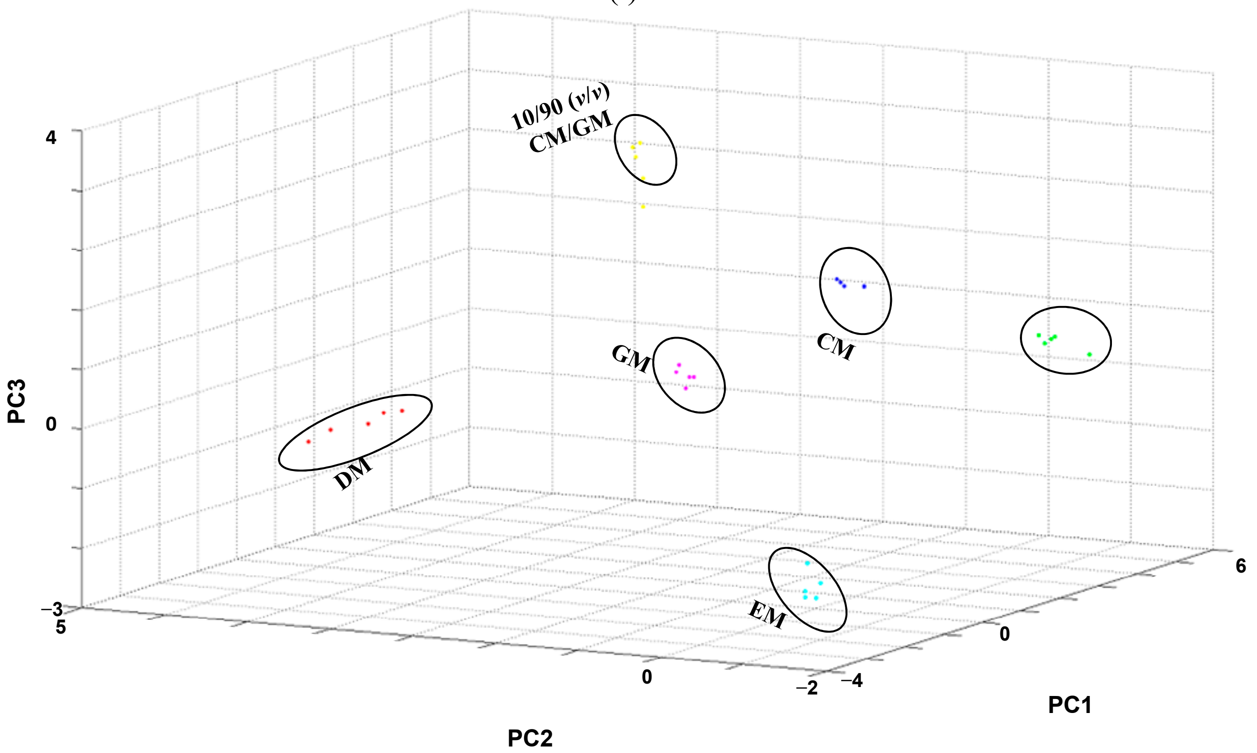

(l)

Figure S5. *Cont.*

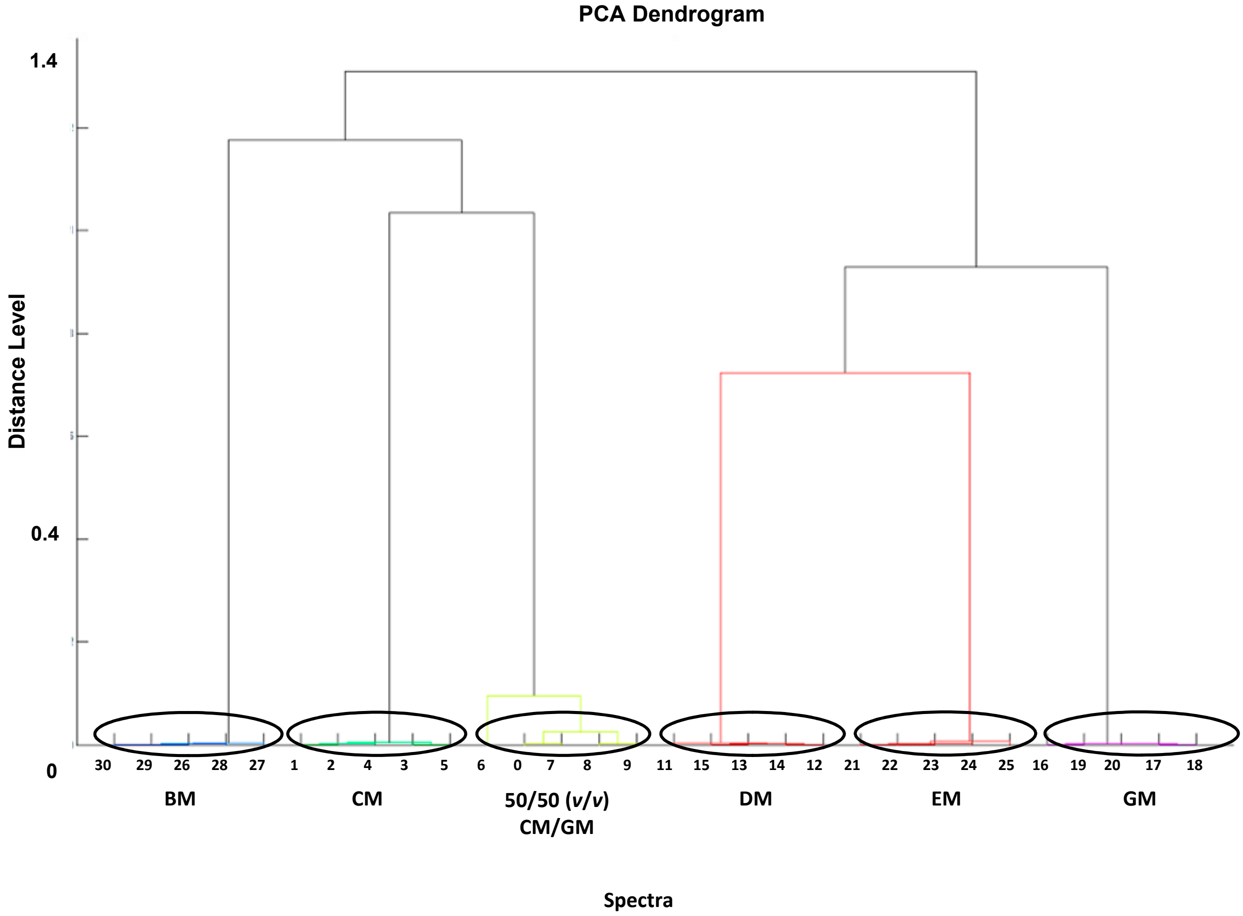

(m)

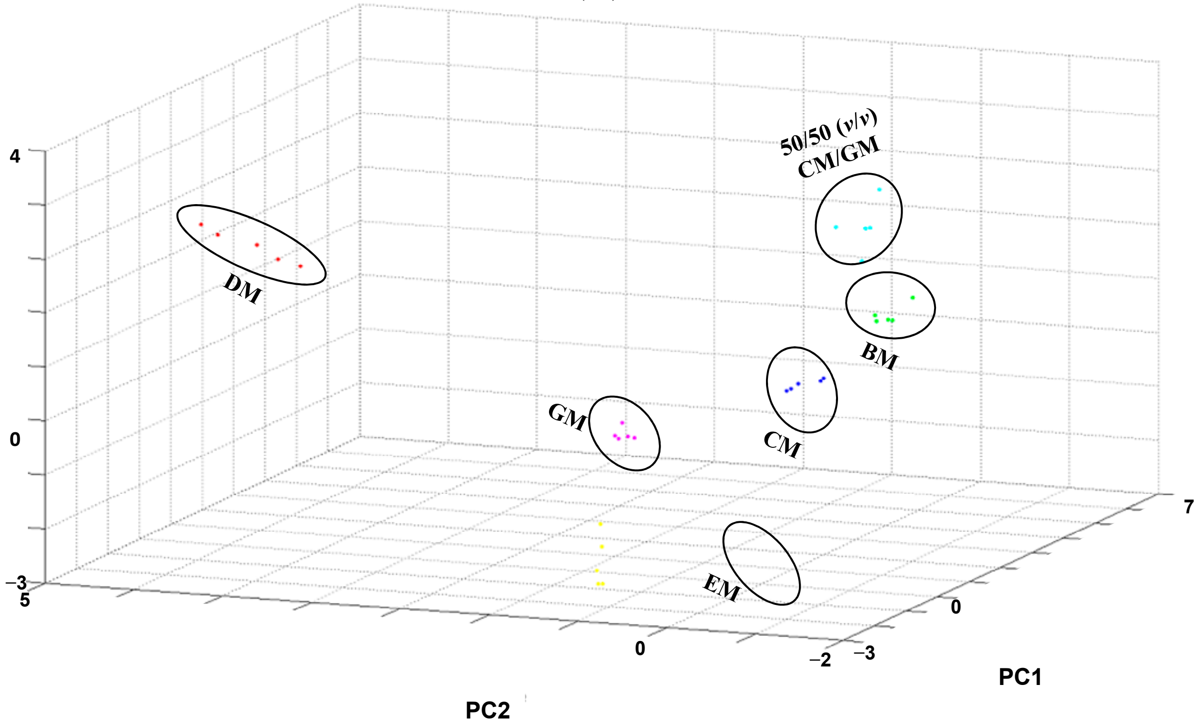

(n)

Figure S5. *Cont.*

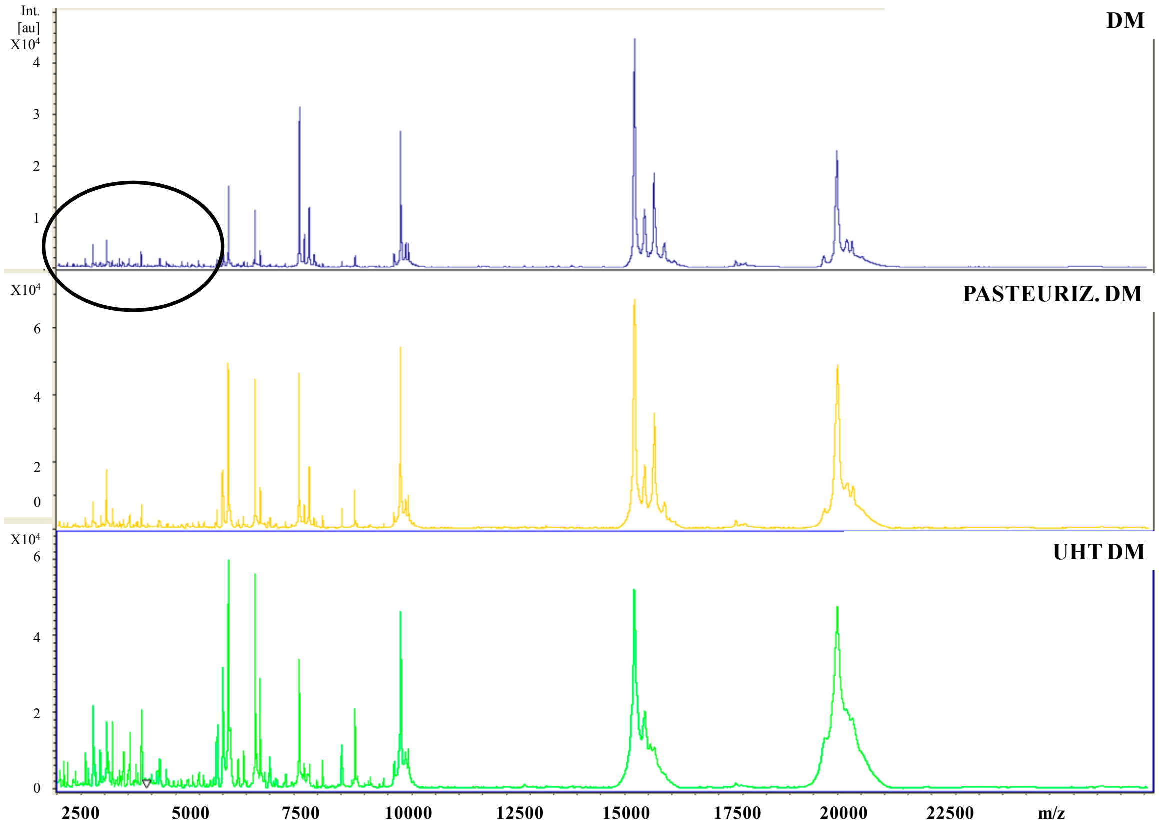

(o)

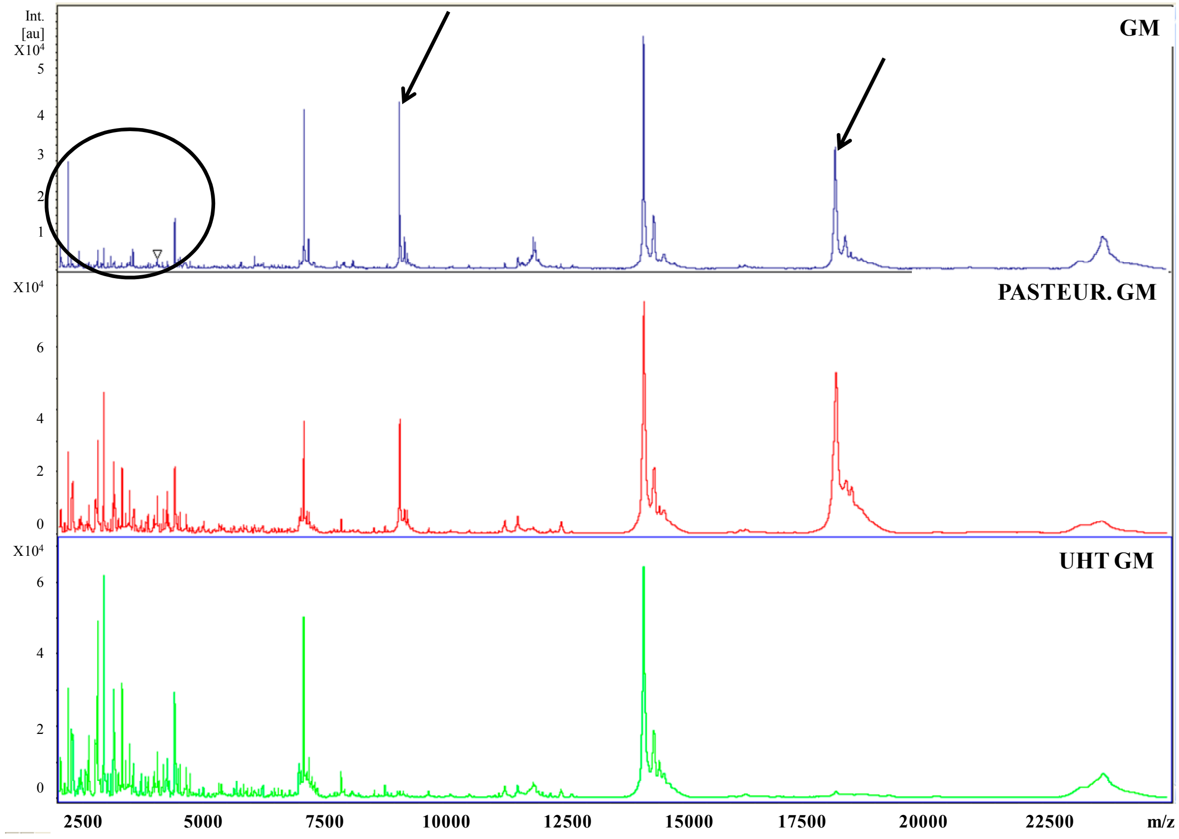

(p)

Figure S6. Pseudo-gel like and MS proteomic profiling of the simulated adulteration of (**a**) DM by BM; (**b**) DM by EM; (**c**) GM by BM; and (**d**) GM by EM. The colour bar, reported on the *Y* axis, indicated the relationship between the colour and the pick intensity and was expressed by arbitrary units (a.u. ×1000), while the mass values (*m*/*z*) were reported on the *X* axis.

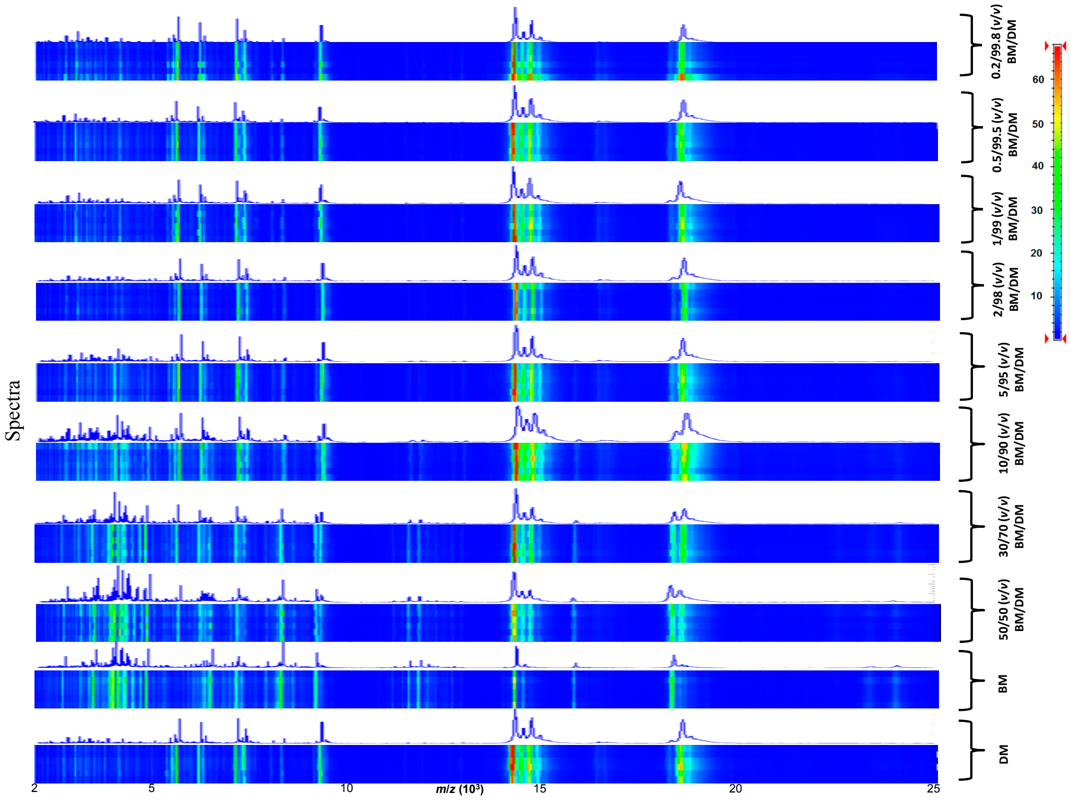

(**a**)

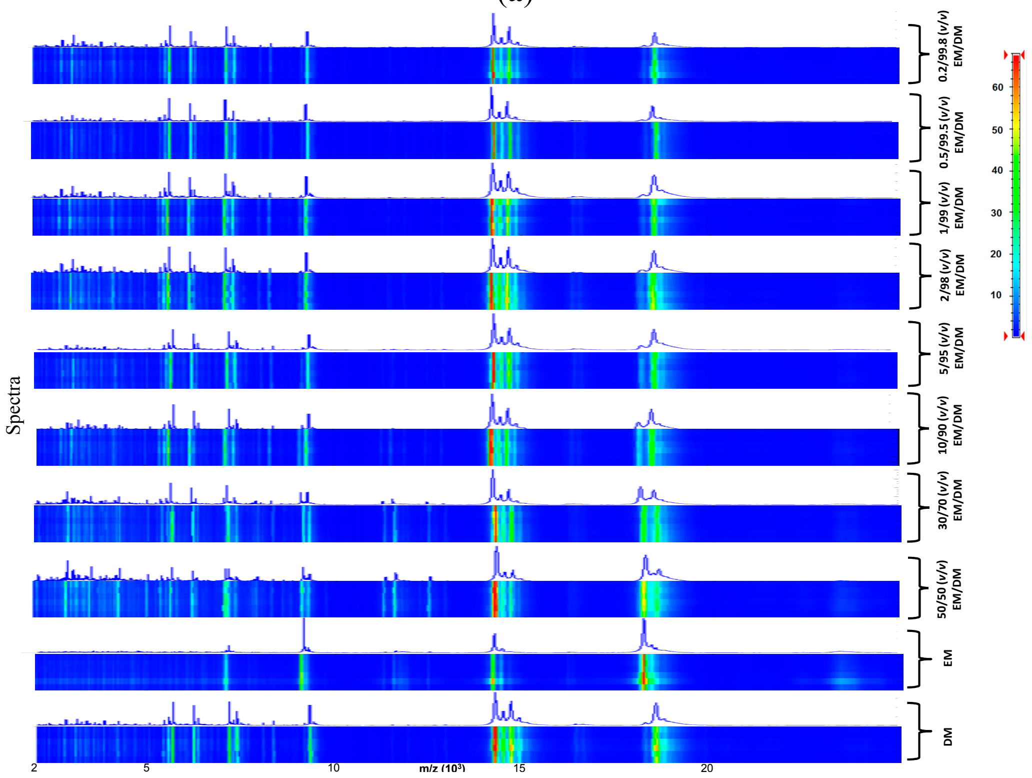

(**b**)

Figure S6. *Cont.*

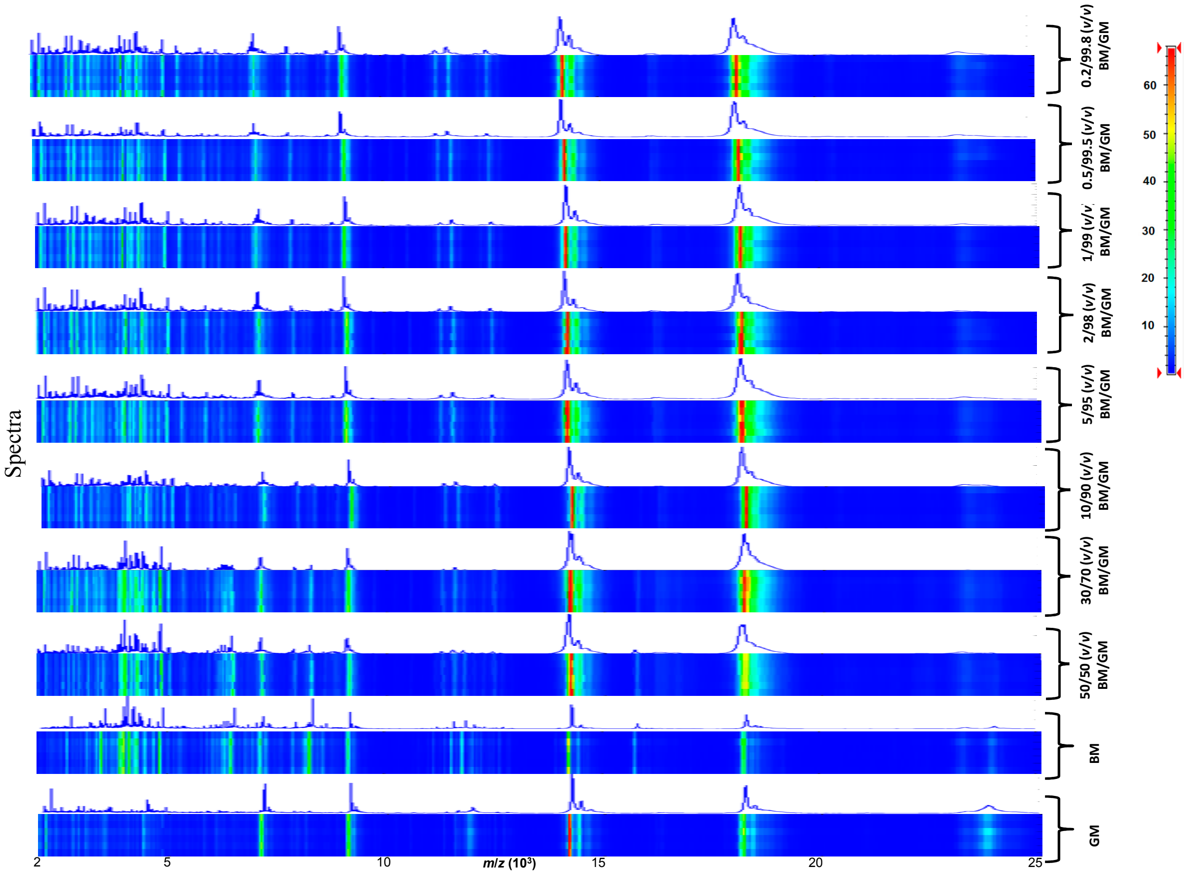

(c)

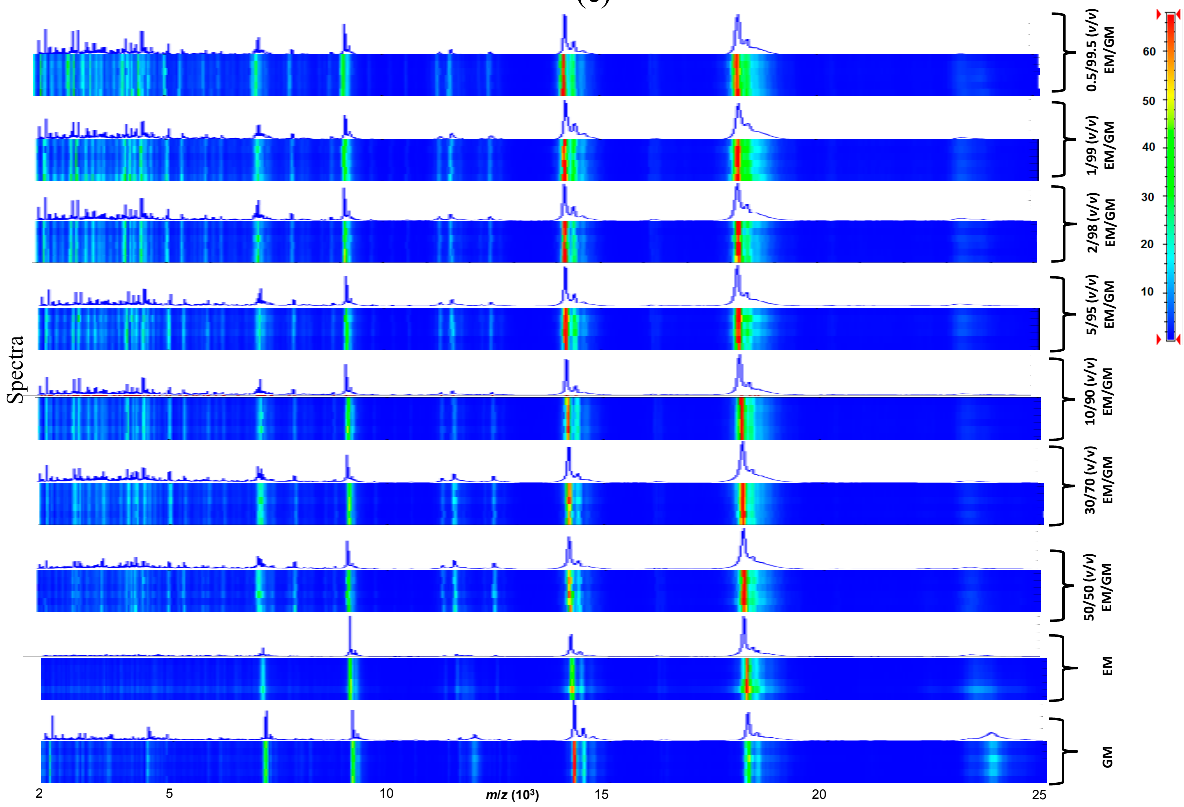

(d)

Figure S7. Mathematical analysis of all spectra replica datasets for the eight mixtures (from 50% to 0.2%) of the simulated adulteration of DM by BM and EM. Hierarchical clustering analysis, producing dendrograms and PCA analysis, producing 3D scatter plot image of DM by BM **(a–h)** and DM by EM (**i**–**r**); Pearson's correlation analysis, producing correlation matrix of DM by BM (**s**) and DM by EM (**t**). The similarity index in the correlation matrix was reported by a scale ranging from 0 to 1 and represented by blue and yellow colours, respectively.

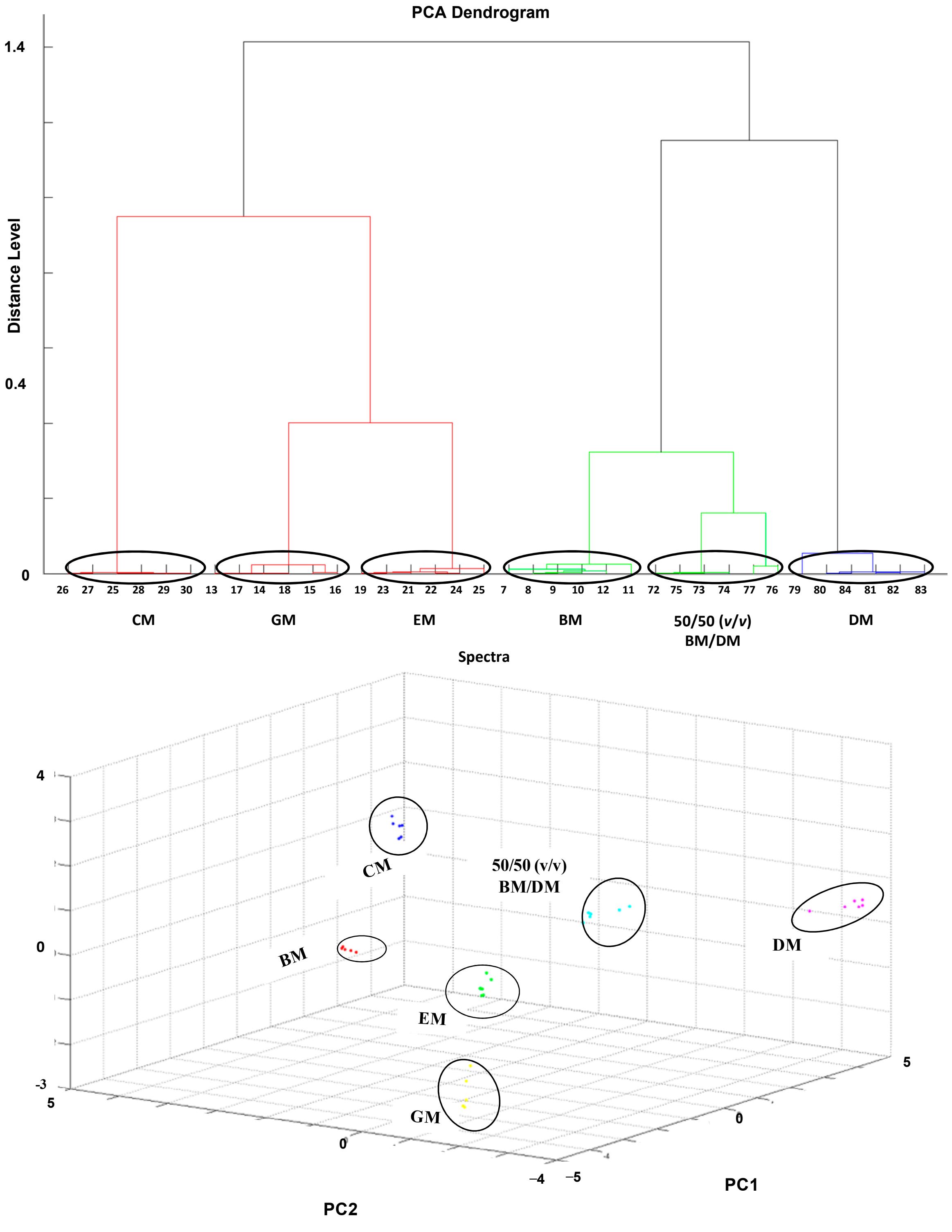

(**a**)

Figure S7. *Cont.*

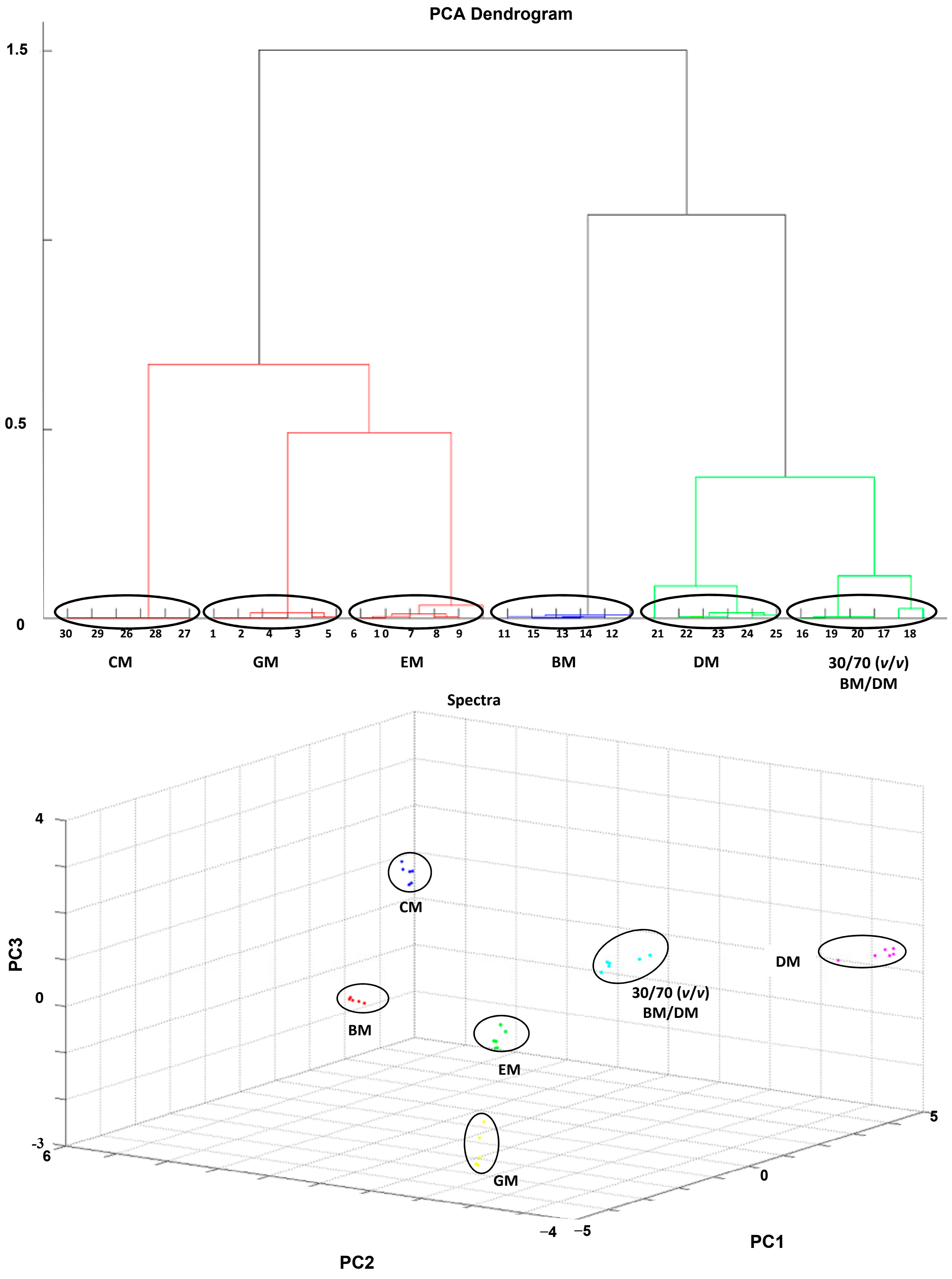

(b)

Figure S7. *Cont.*

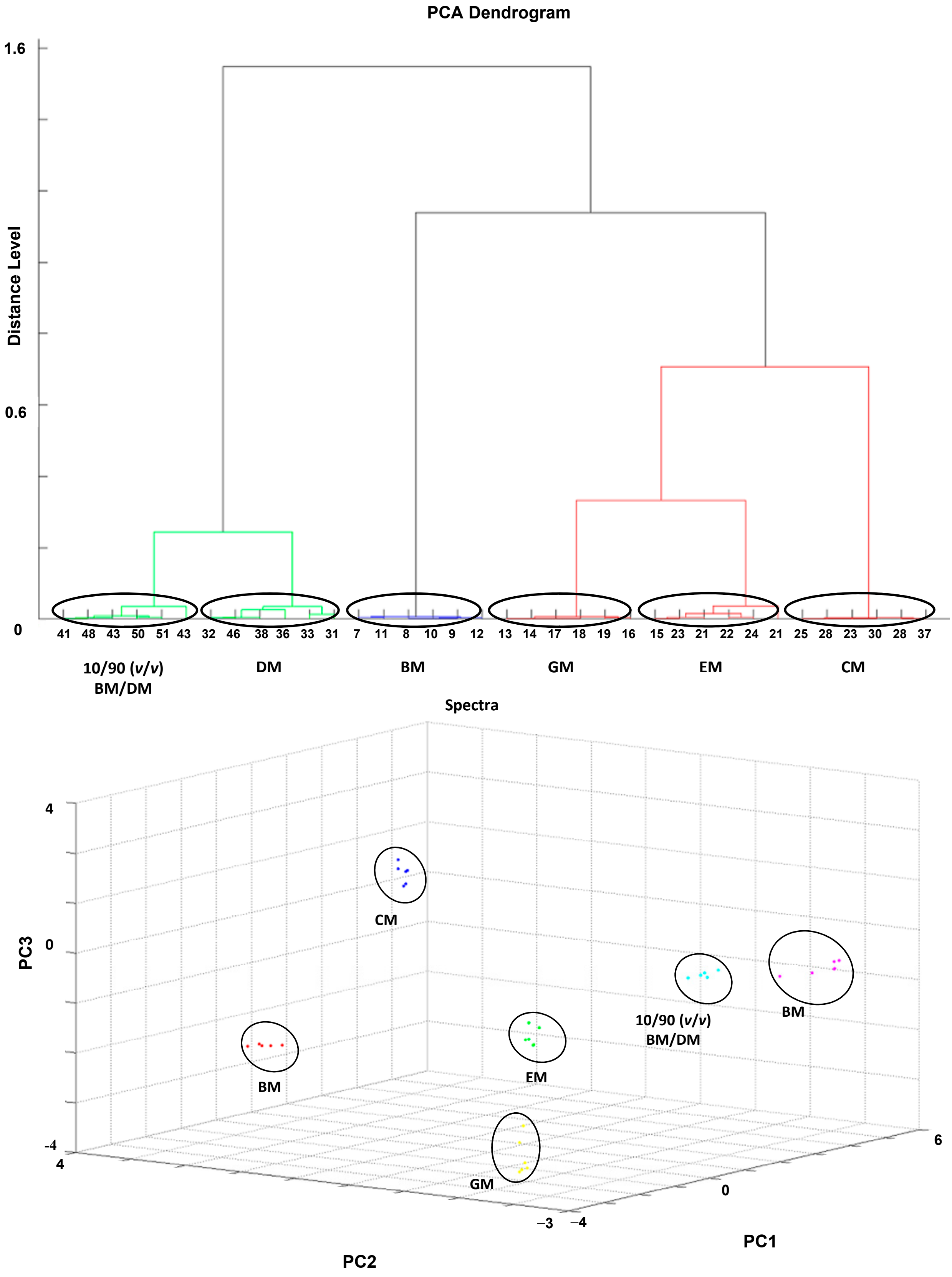

(c)

Figure S7. *Cont.*

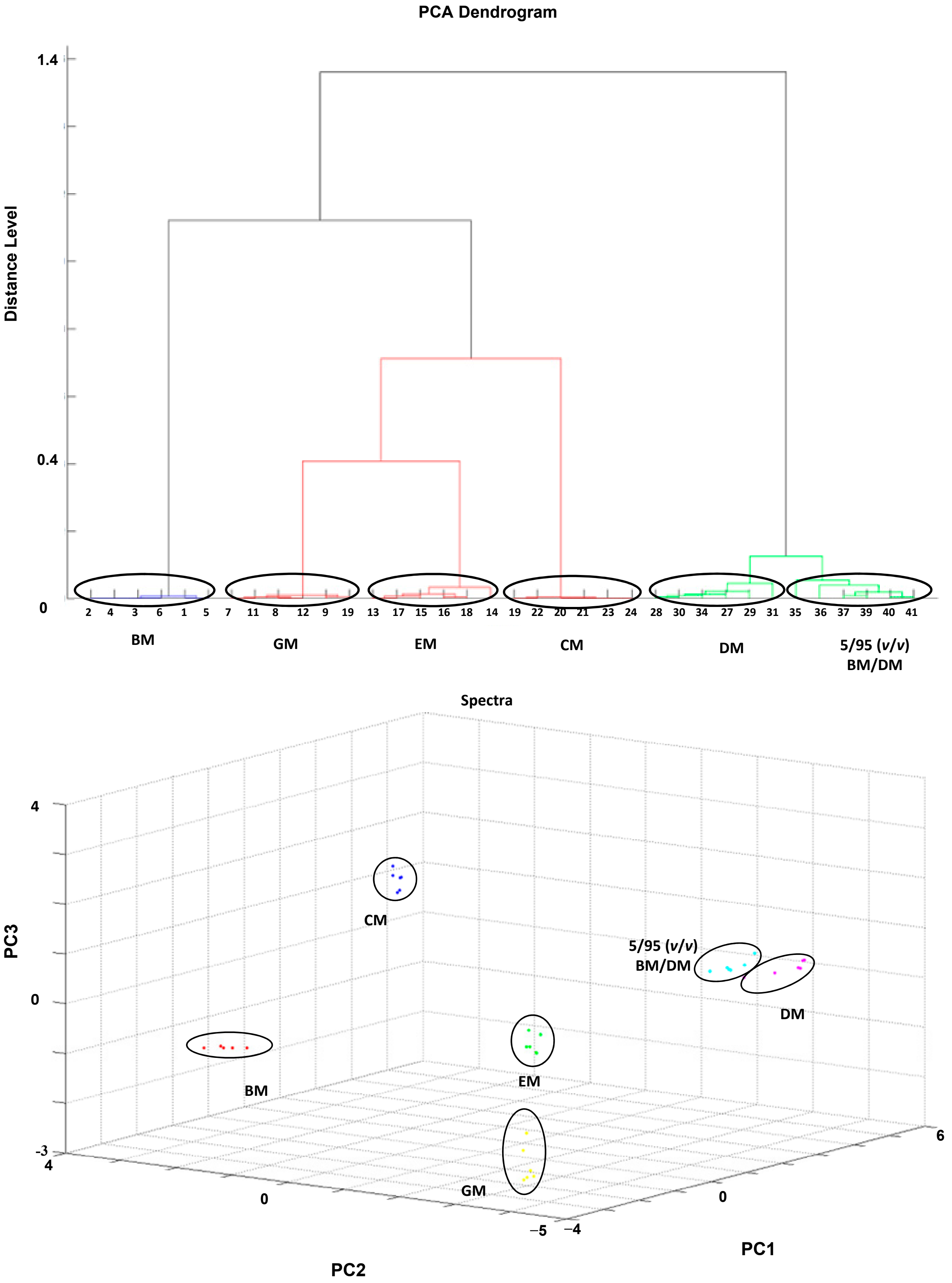

(d)

Figure S7. *Cont.*

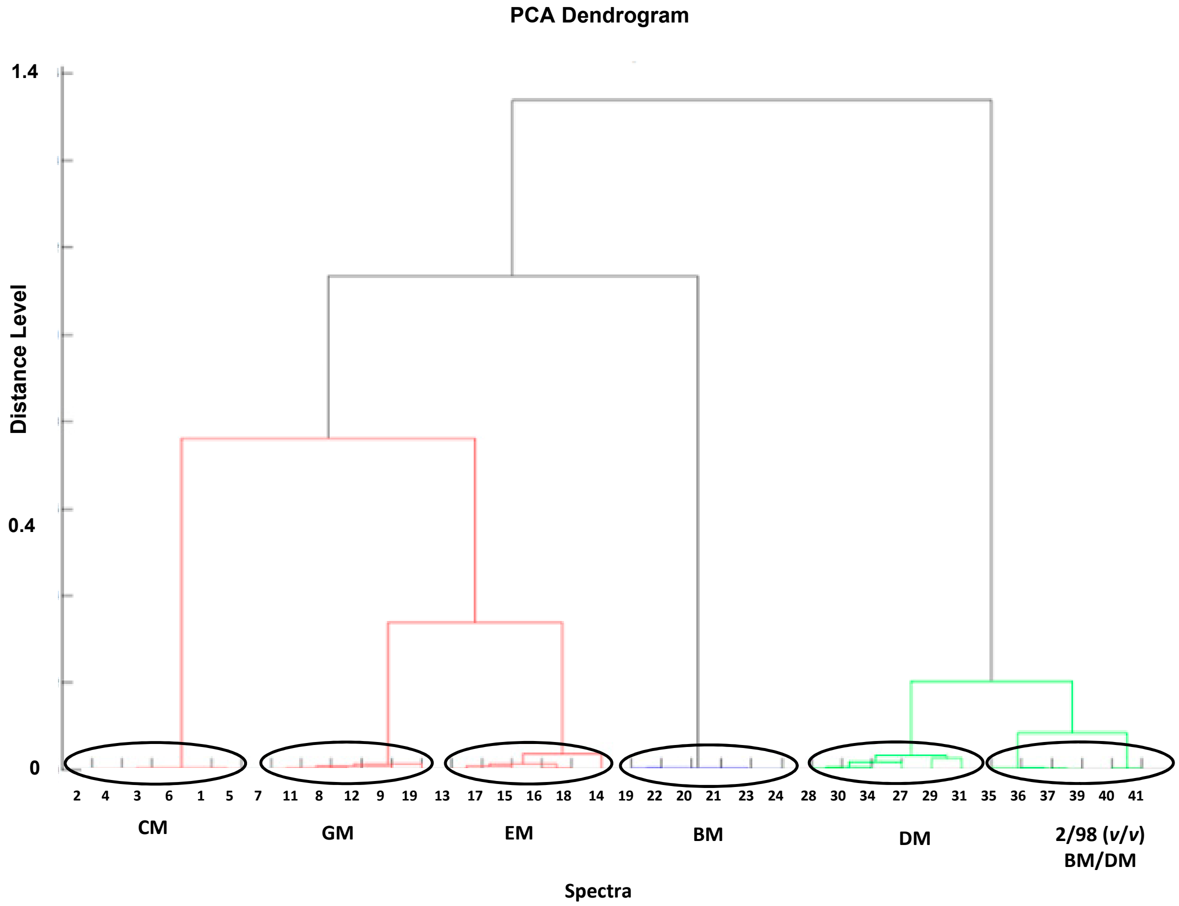

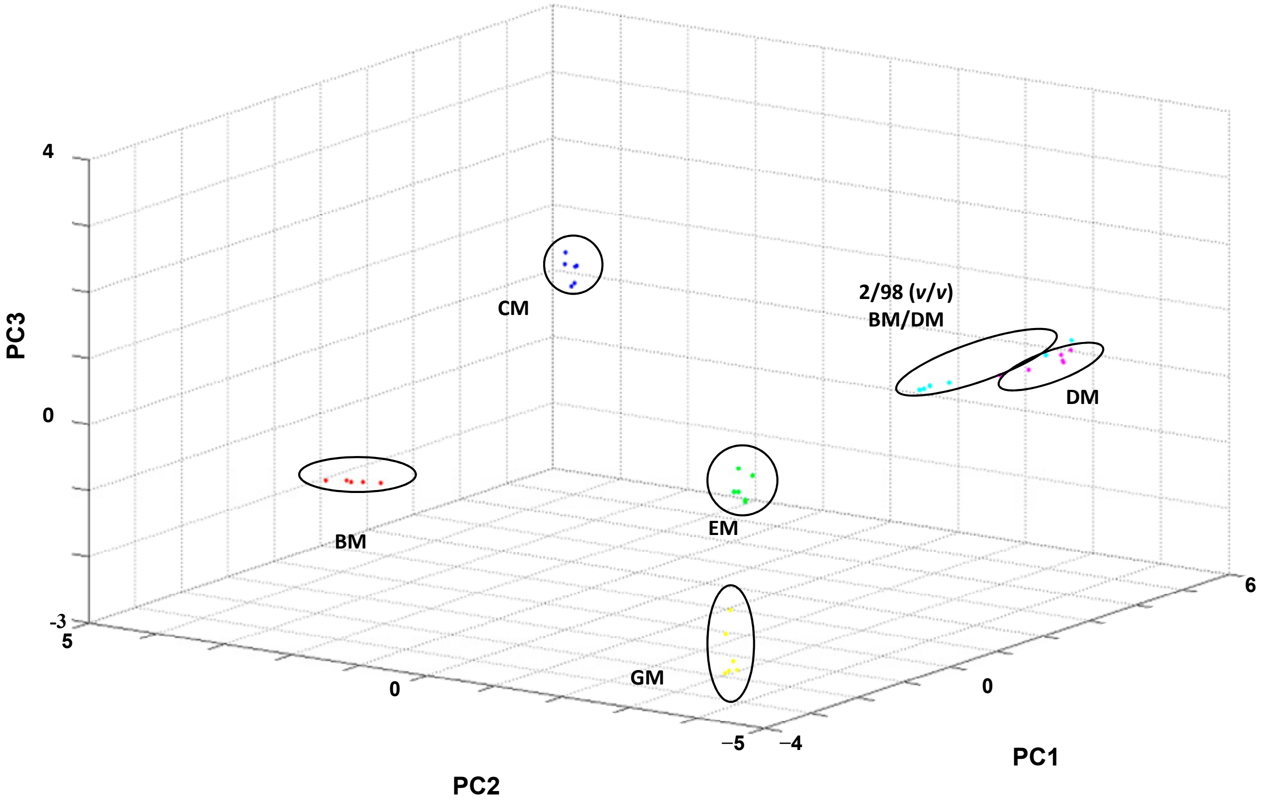

(e)

Figure S7. *Cont.*

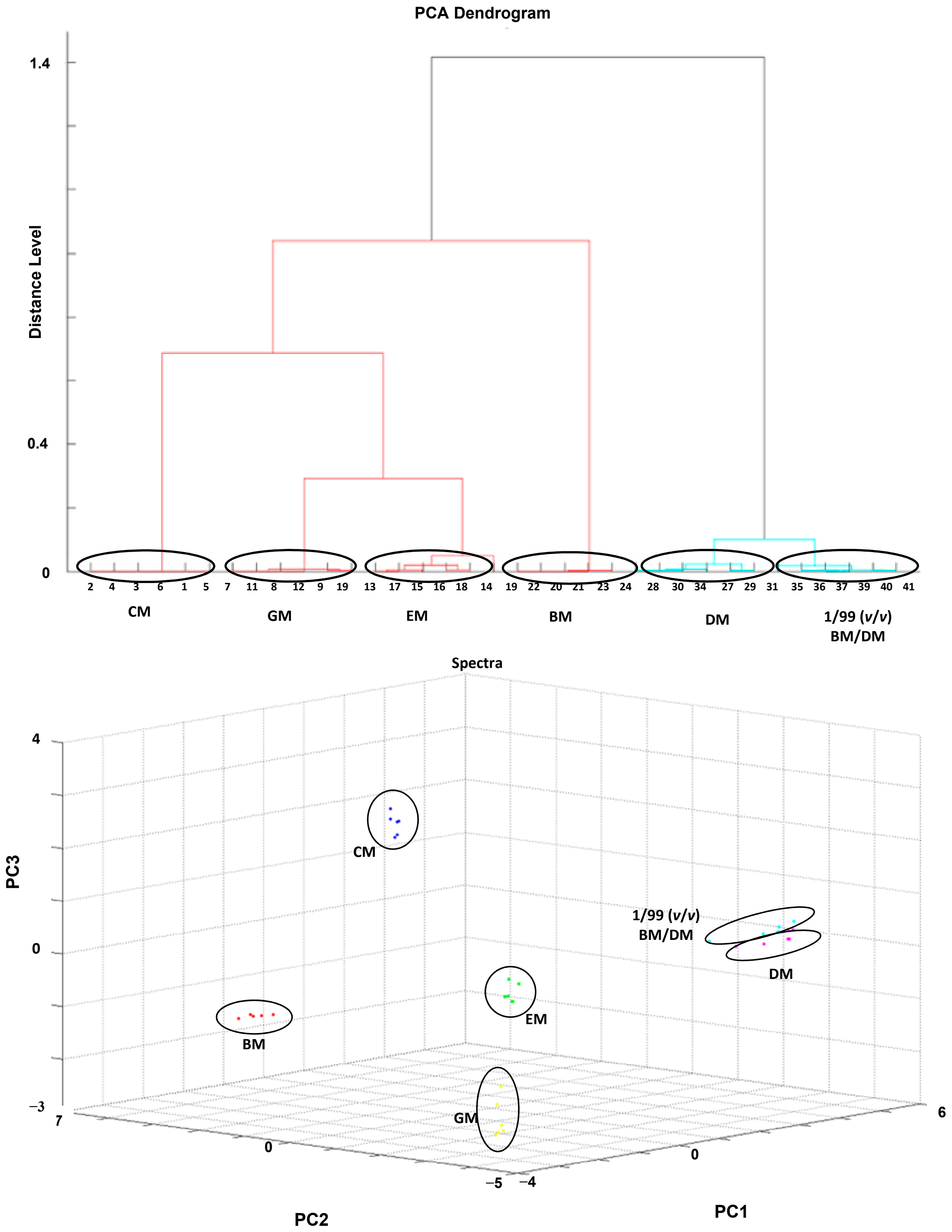

(f)

Figure S7. *Cont.*

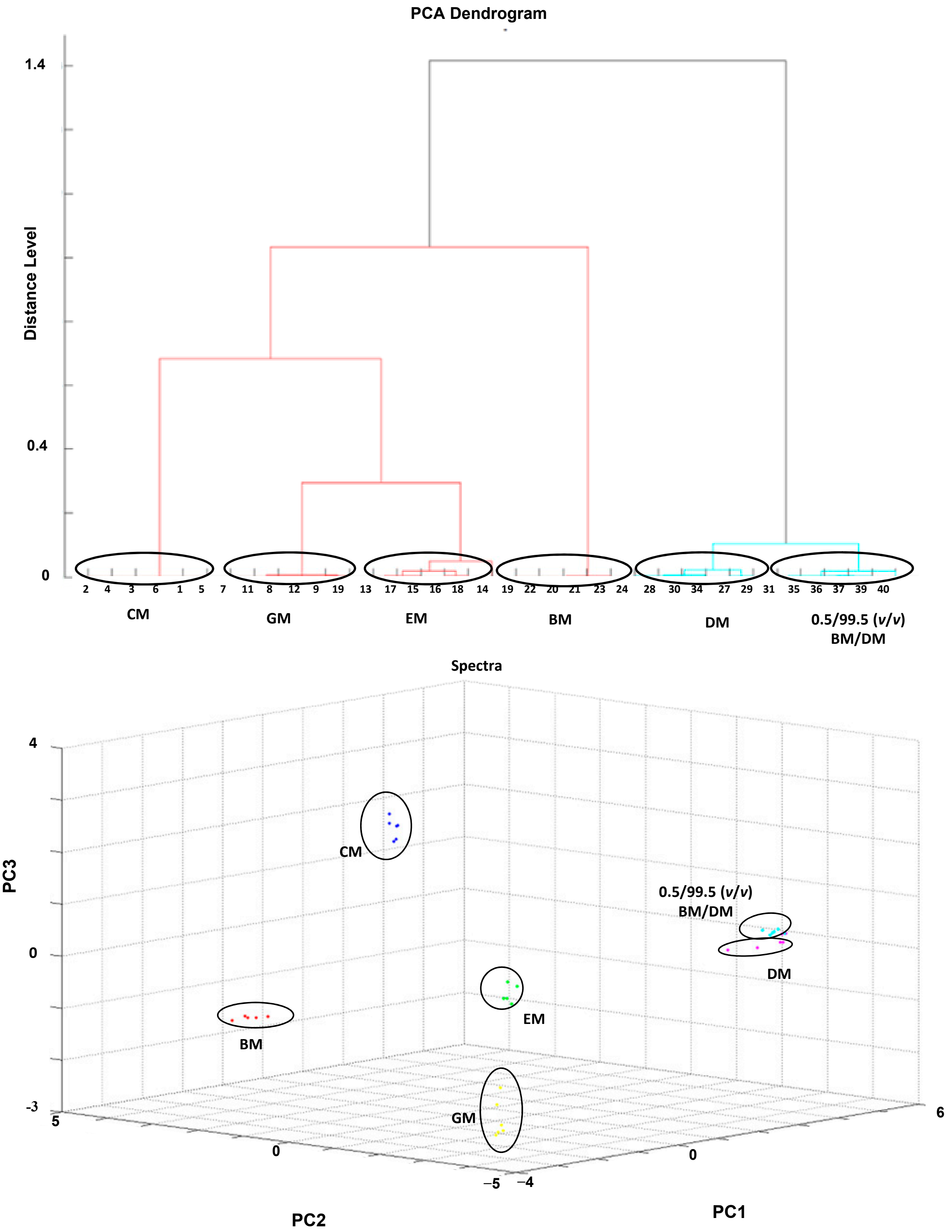

(g)

Figure S7. *Cont.*

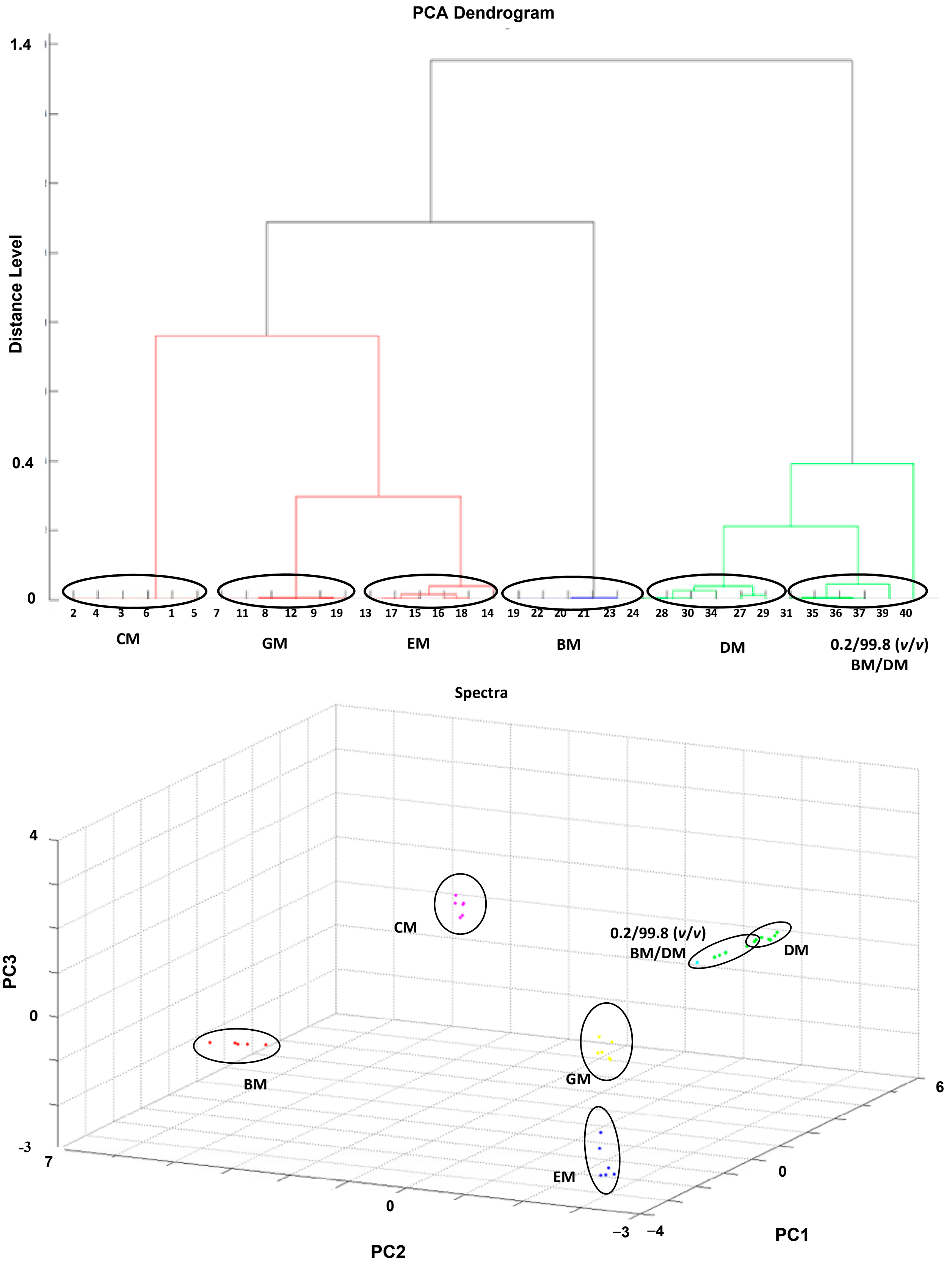

(h)

Figure S7. *Cont.*

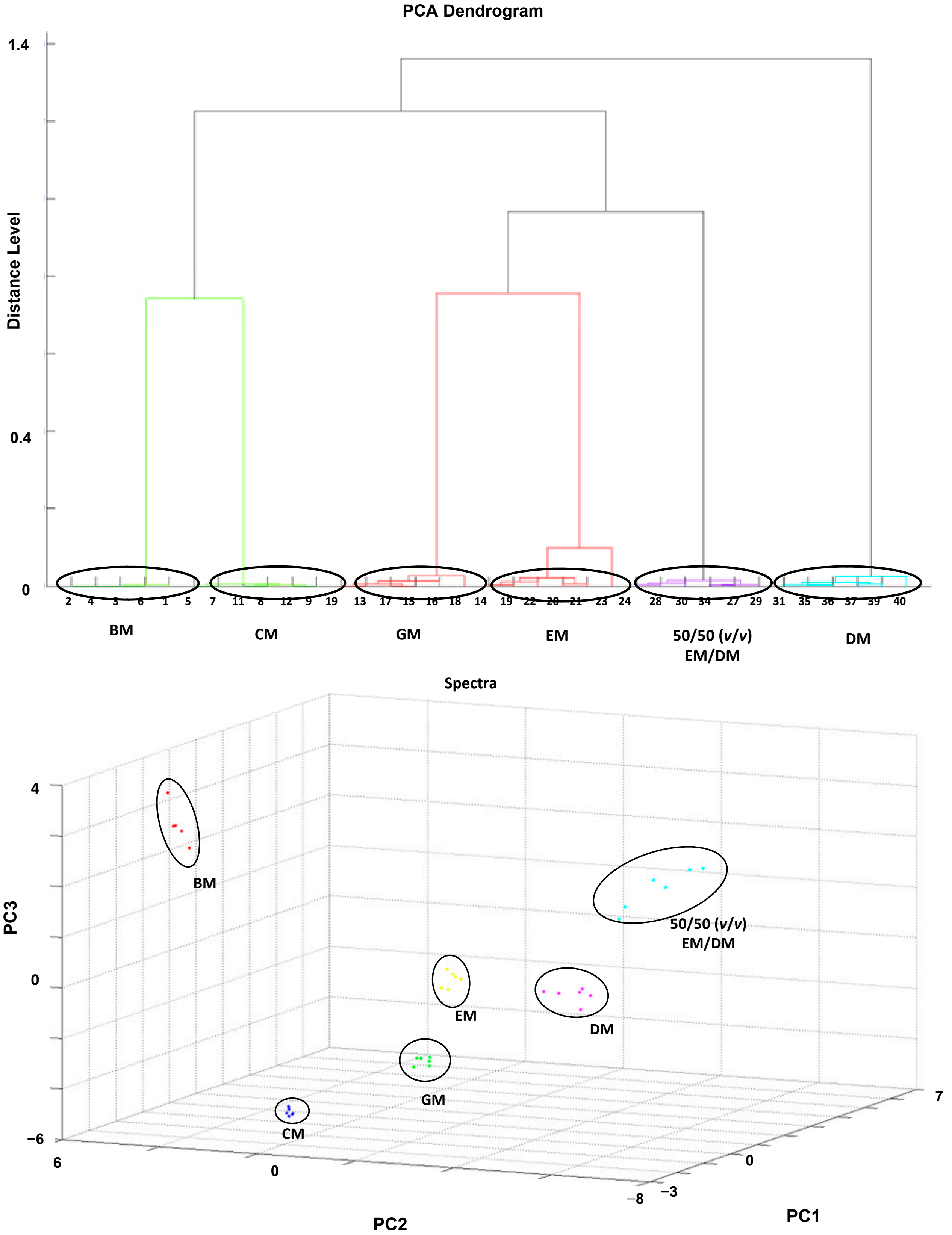

(i)

Figure S7. *Cont.*

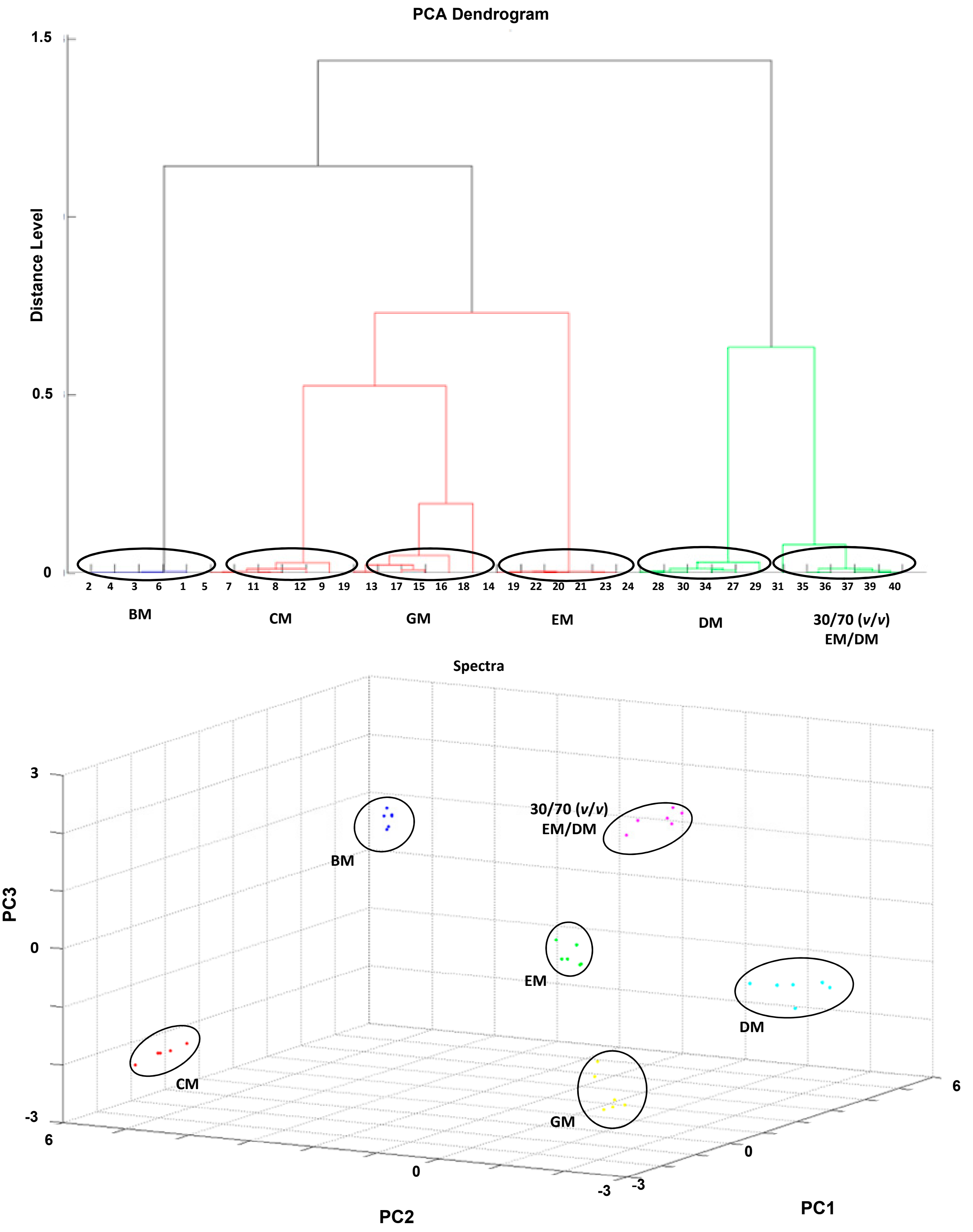

(I)

Figure S7. *Cont.*

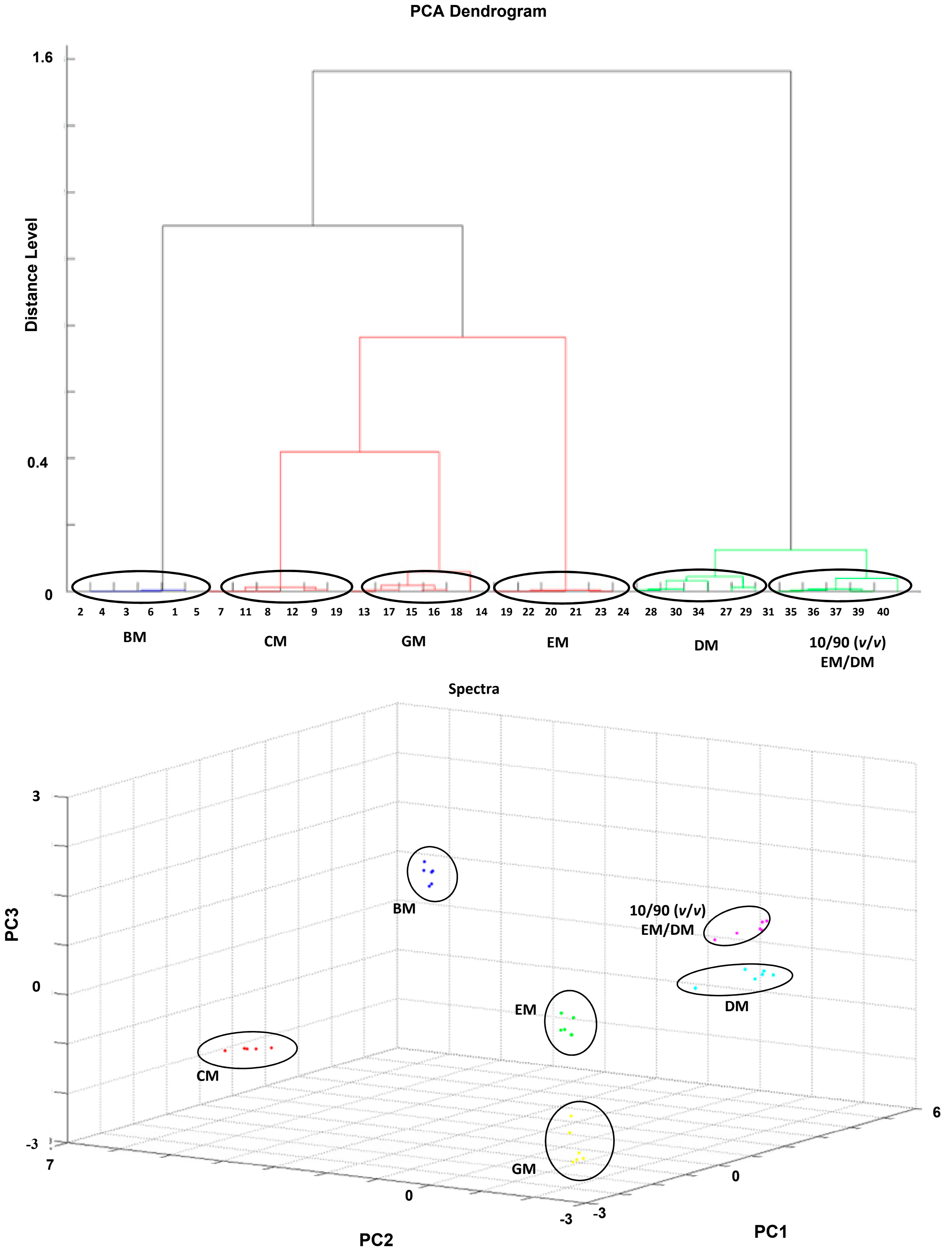

(m)

Figure S7. *Cont.*

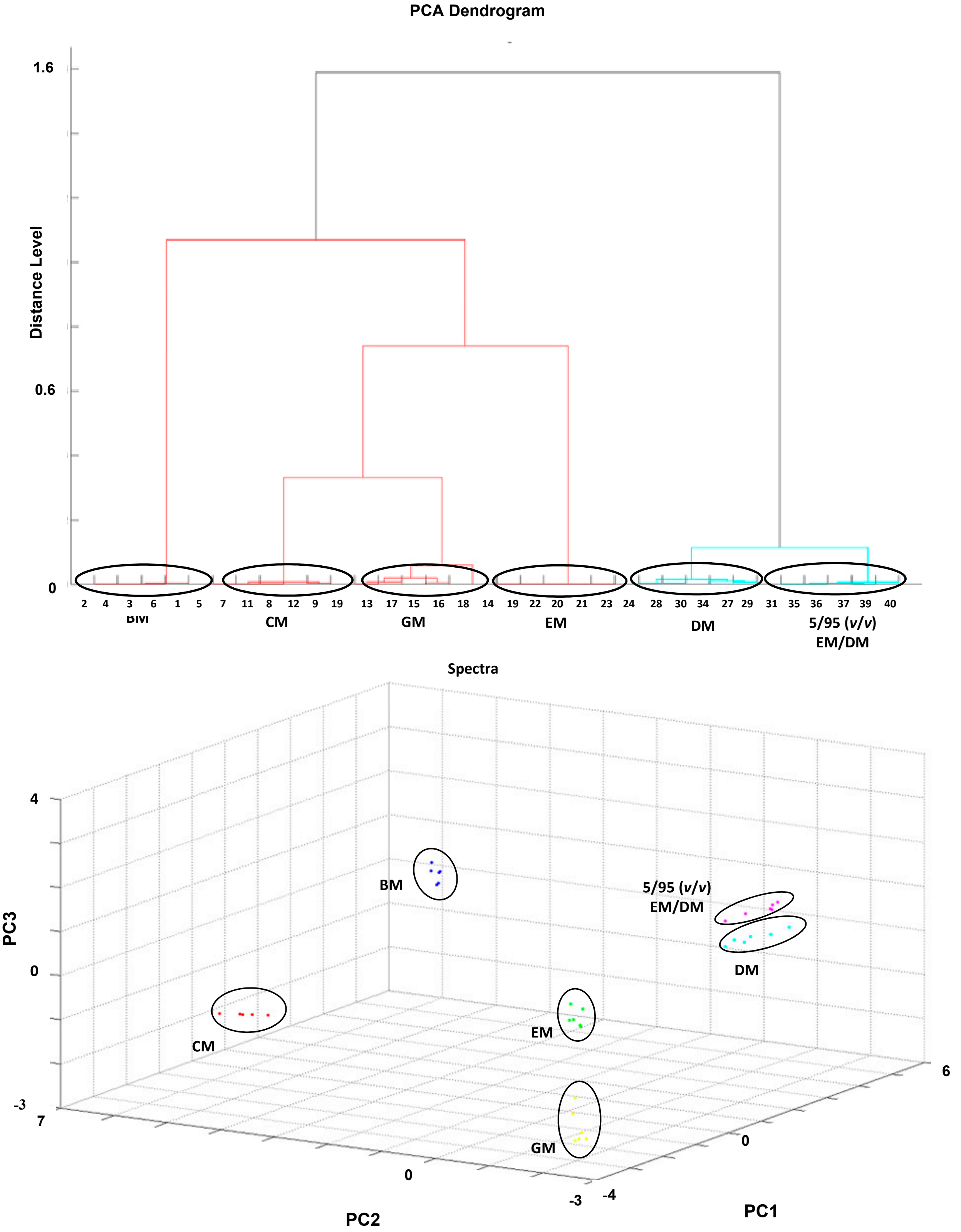

(n)

Figure S7. *Cont.*

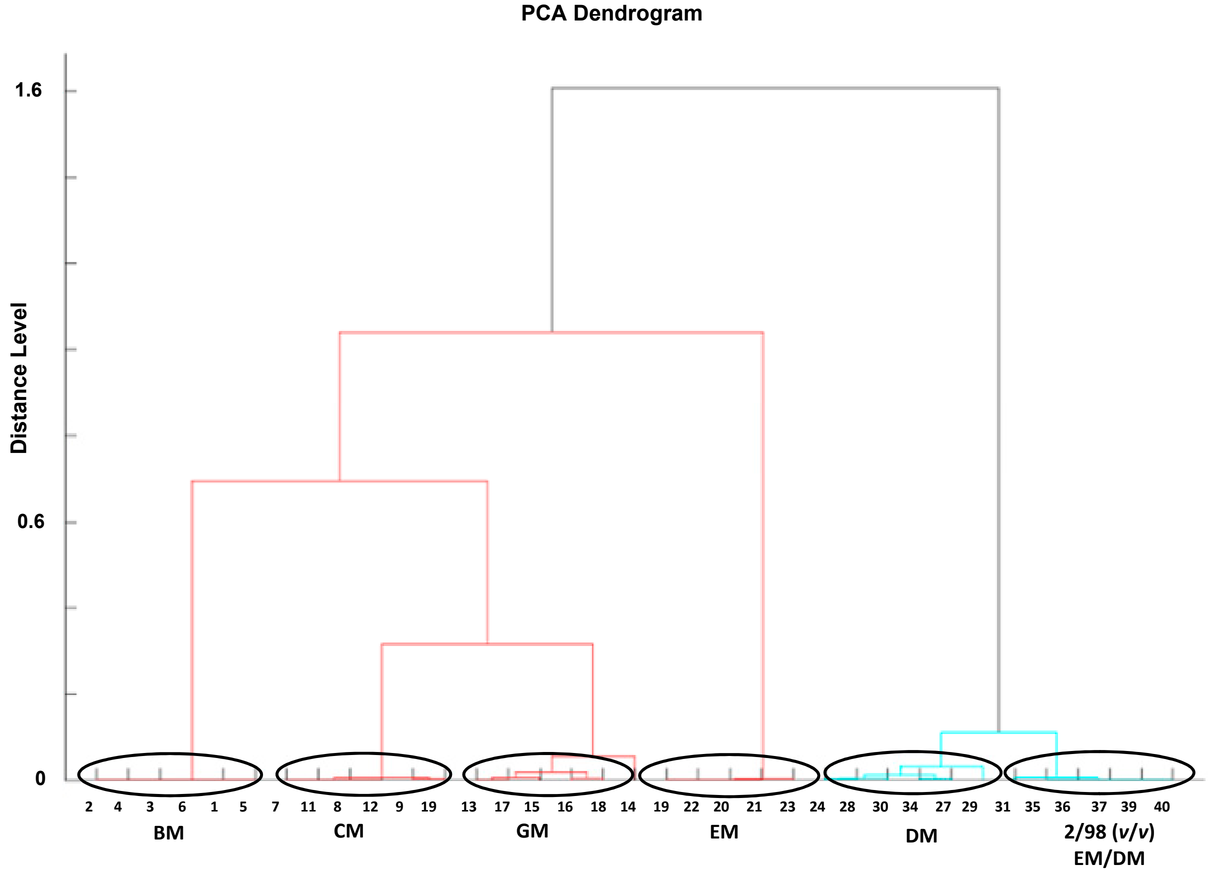

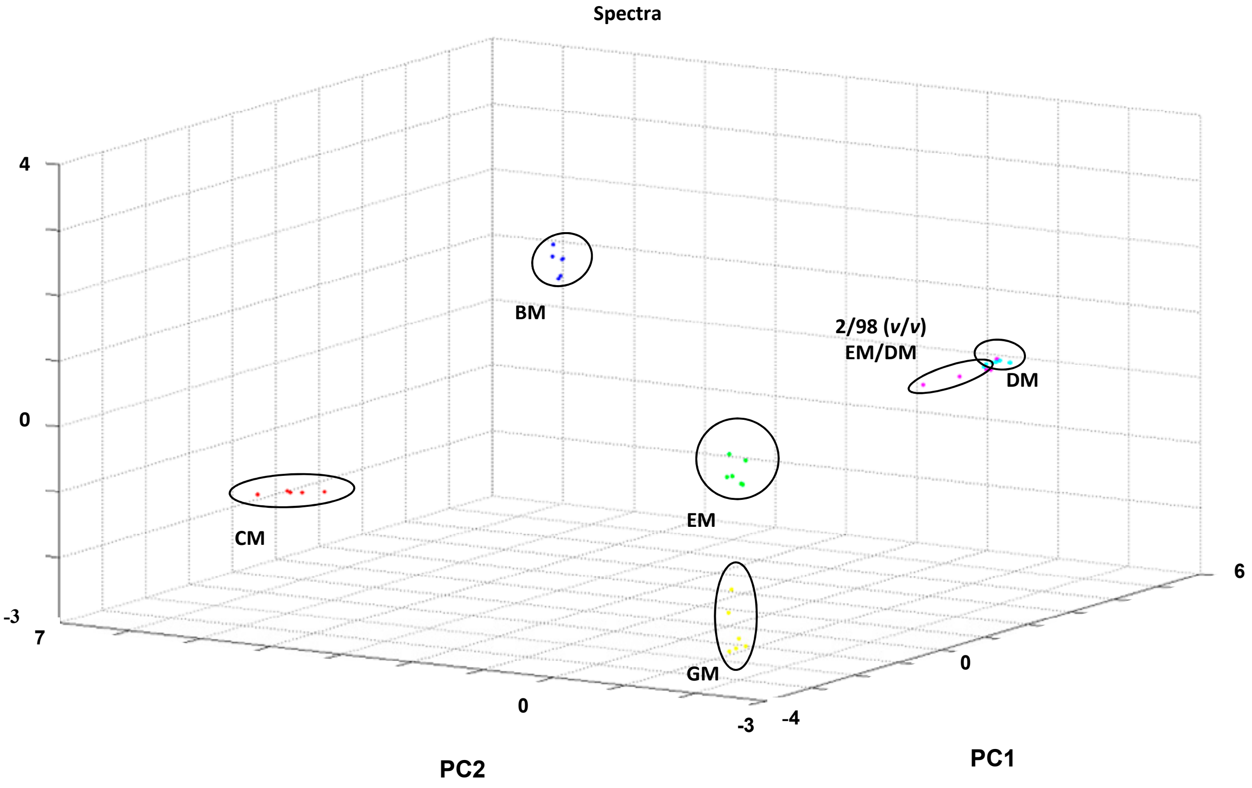

(o)

Figure S7. *Cont.*

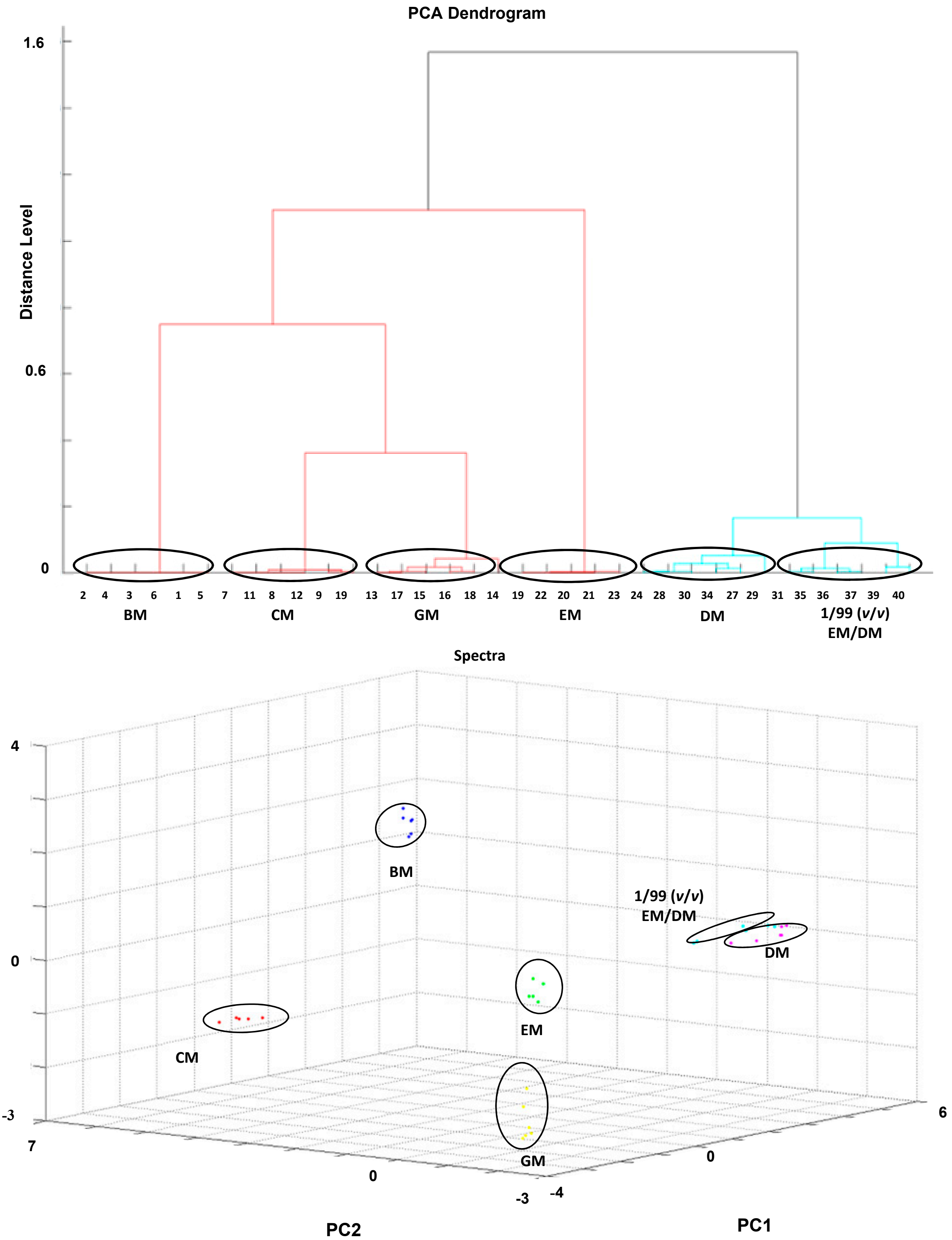

(p)

Figure S7. *Cont.*

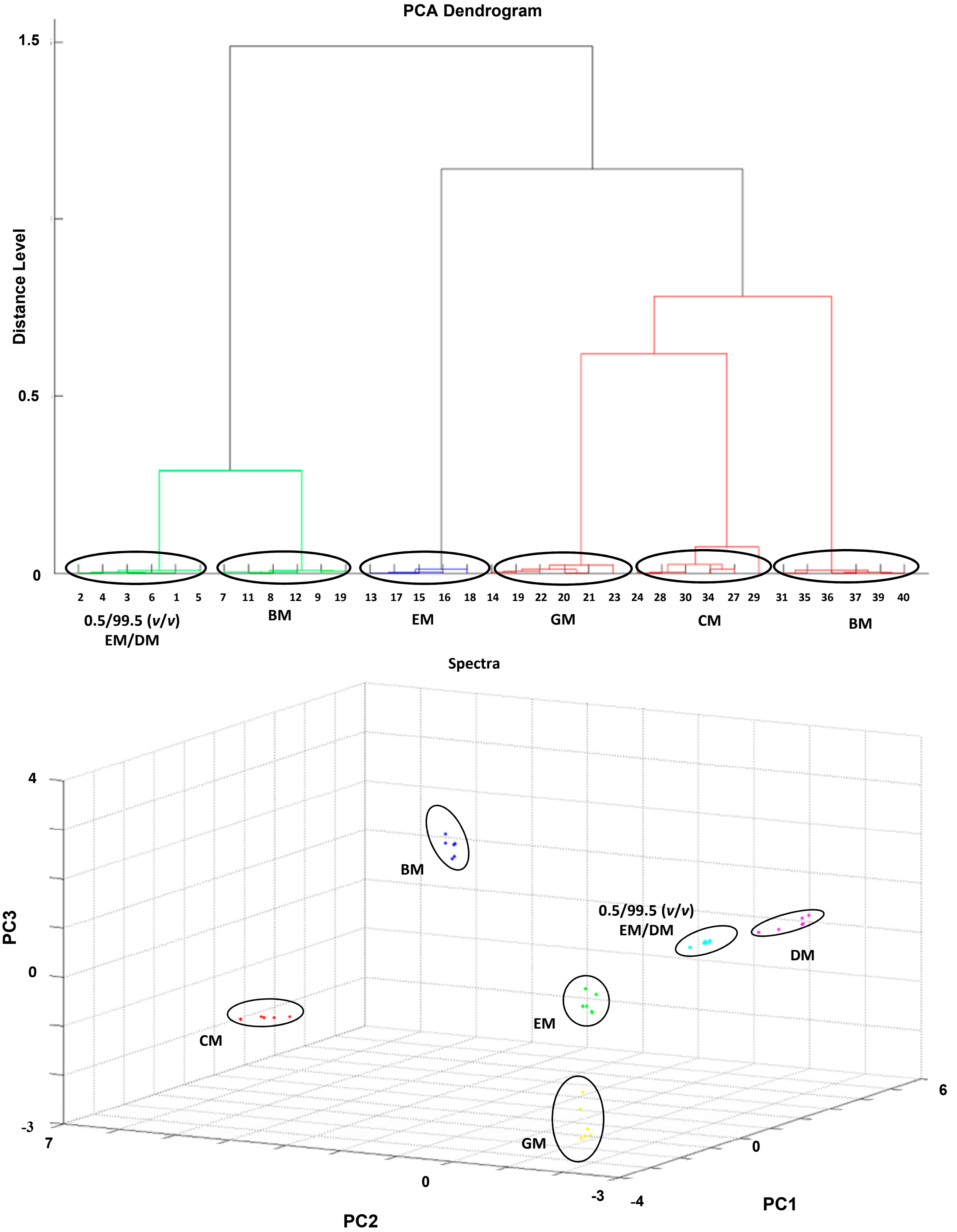

(q)

Figure S7. *Cont.*

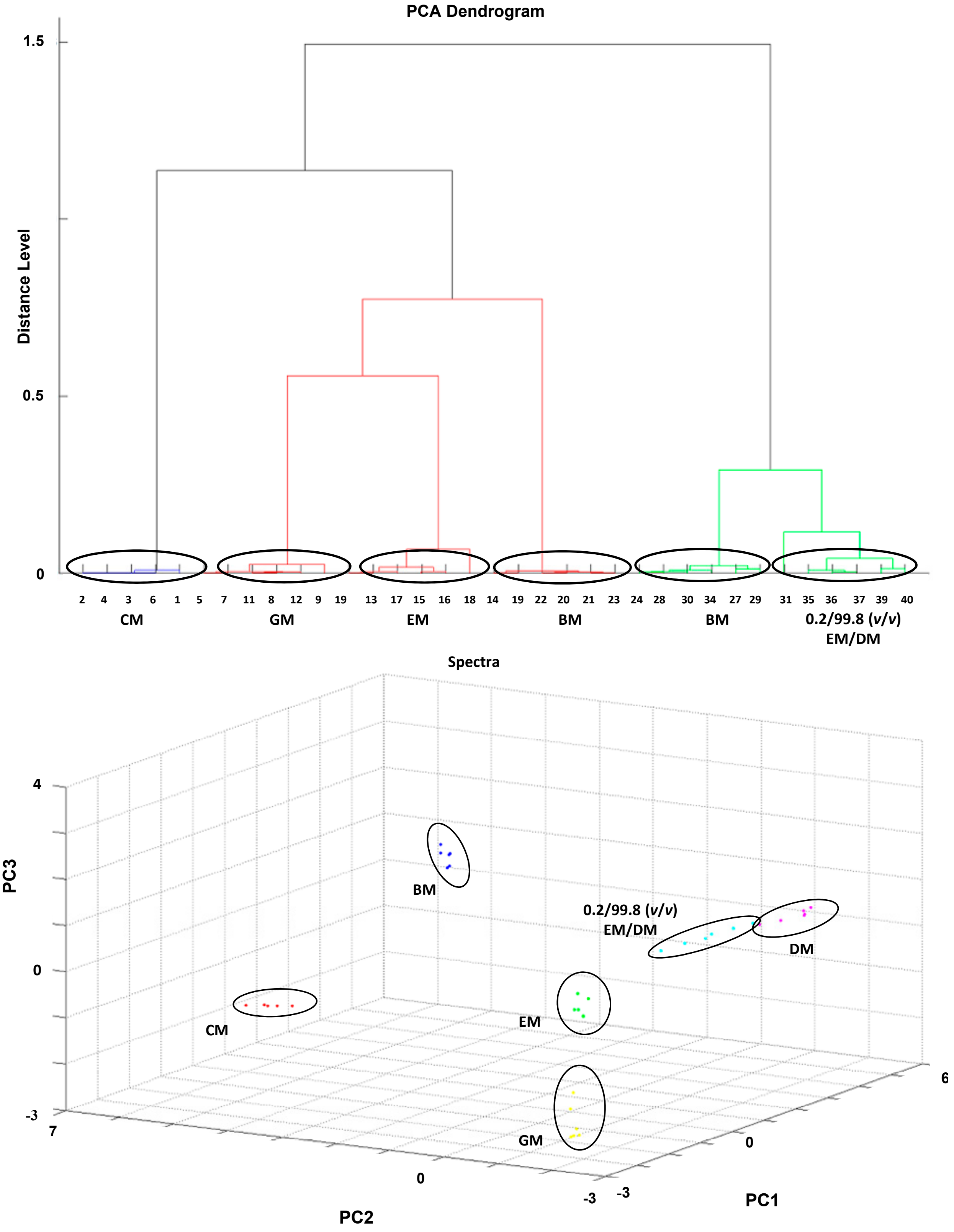

(**r**)

Figure S7. *Cont.*

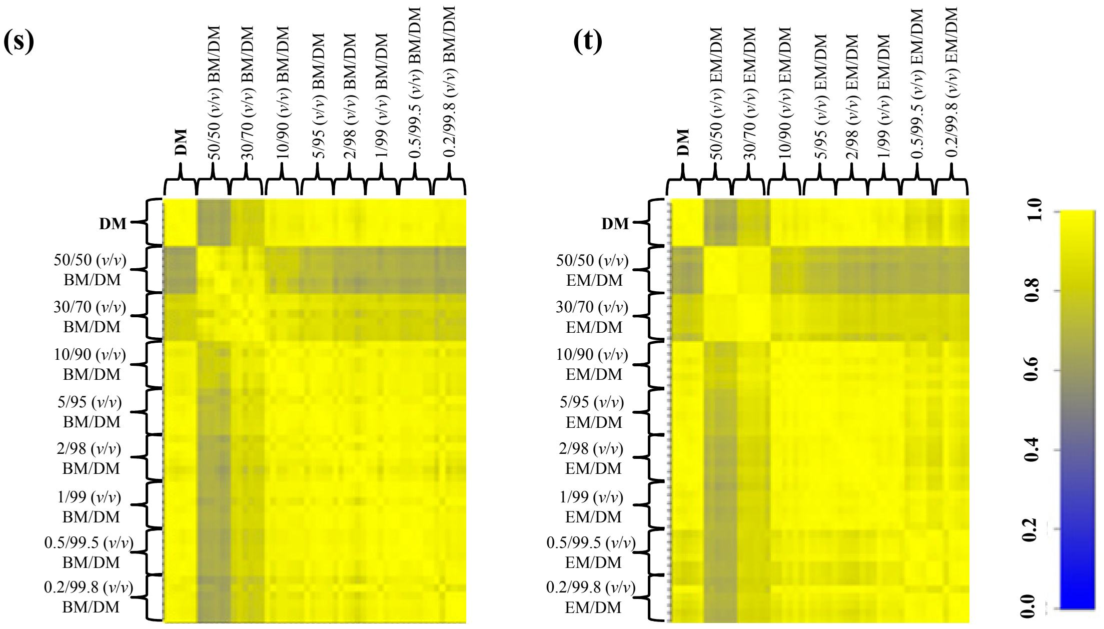

Figure S8. Mathematical analysis of all spectra replica datasets for the eight mixtures (from 50% to 0.2%) of the simulated adulteration of GM by BM and EM. Hierarchical clustering analysis, producing dendrograms and PCA analysis, producing 3D scatter plot image of GM by BM **(a–h)** and GM by EM (**i–q**); Pearson's correlation analysis, producing correlation matrix of GM by BM (**r**) and GM by EM (**s**). The similarity index in the correlation matrix was reported by a scale ranging from 0 to 1 and represented by blue and yellow colours, respectively.

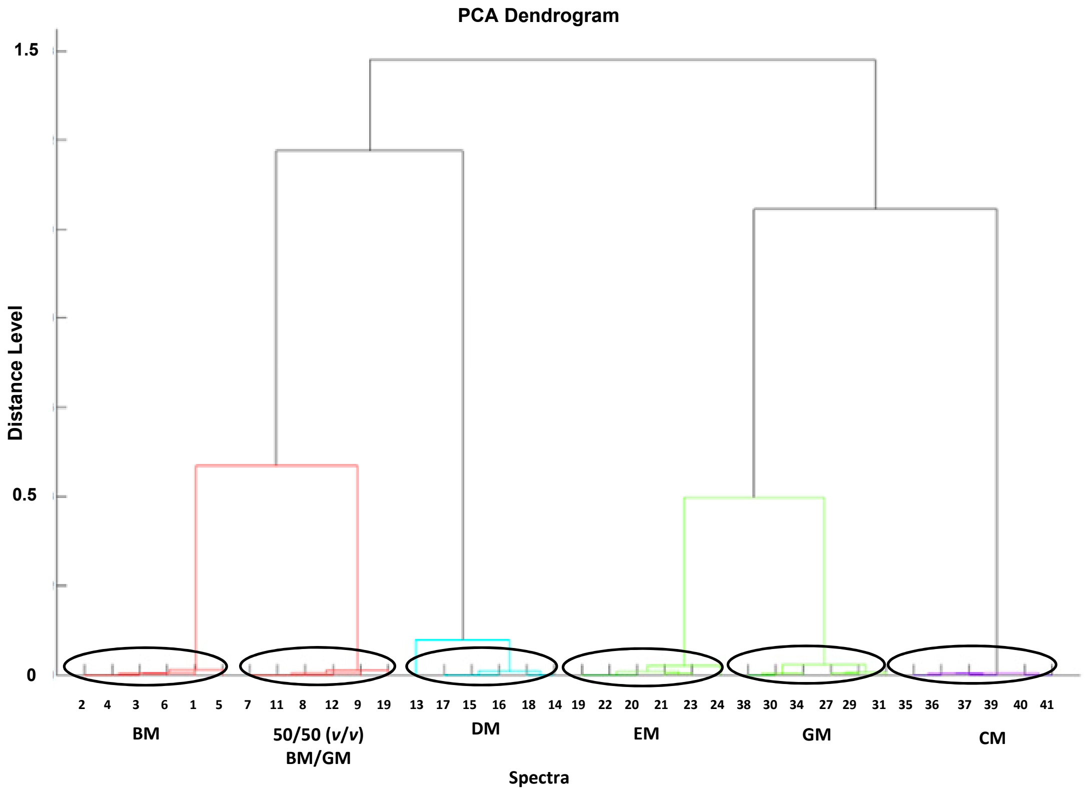

Figure S8. *Cont.*

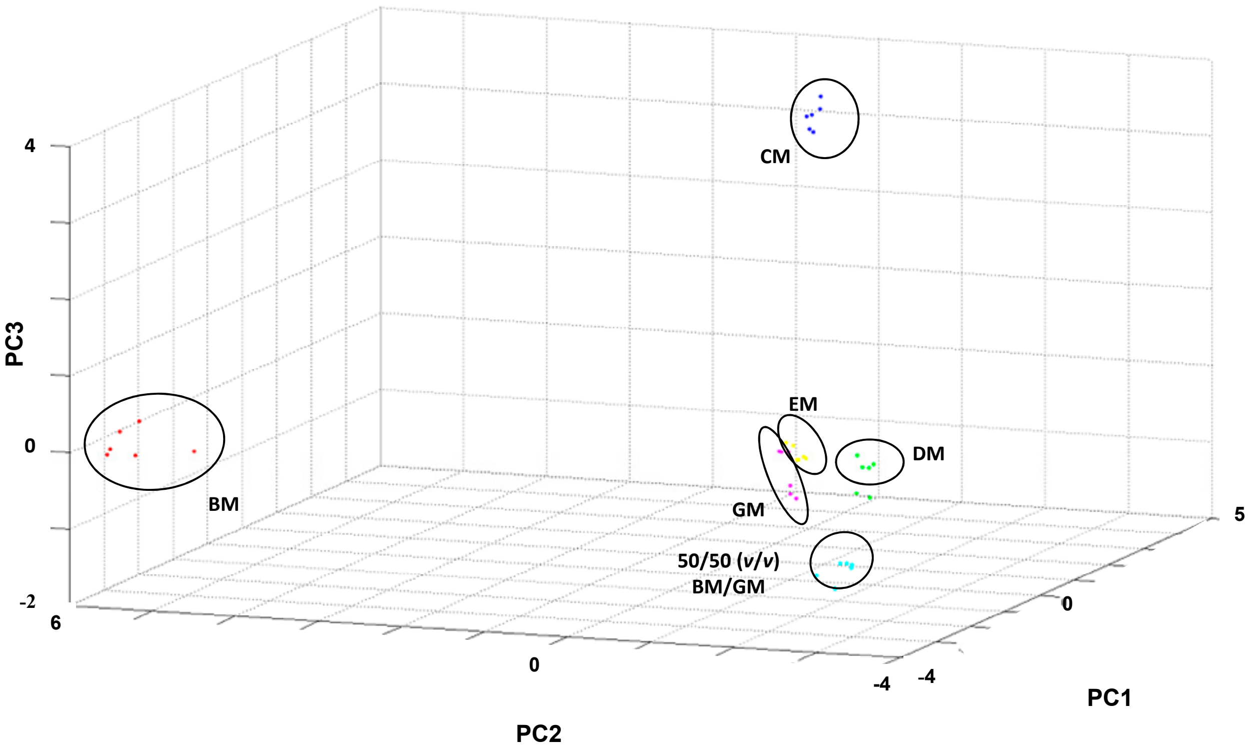

(a)

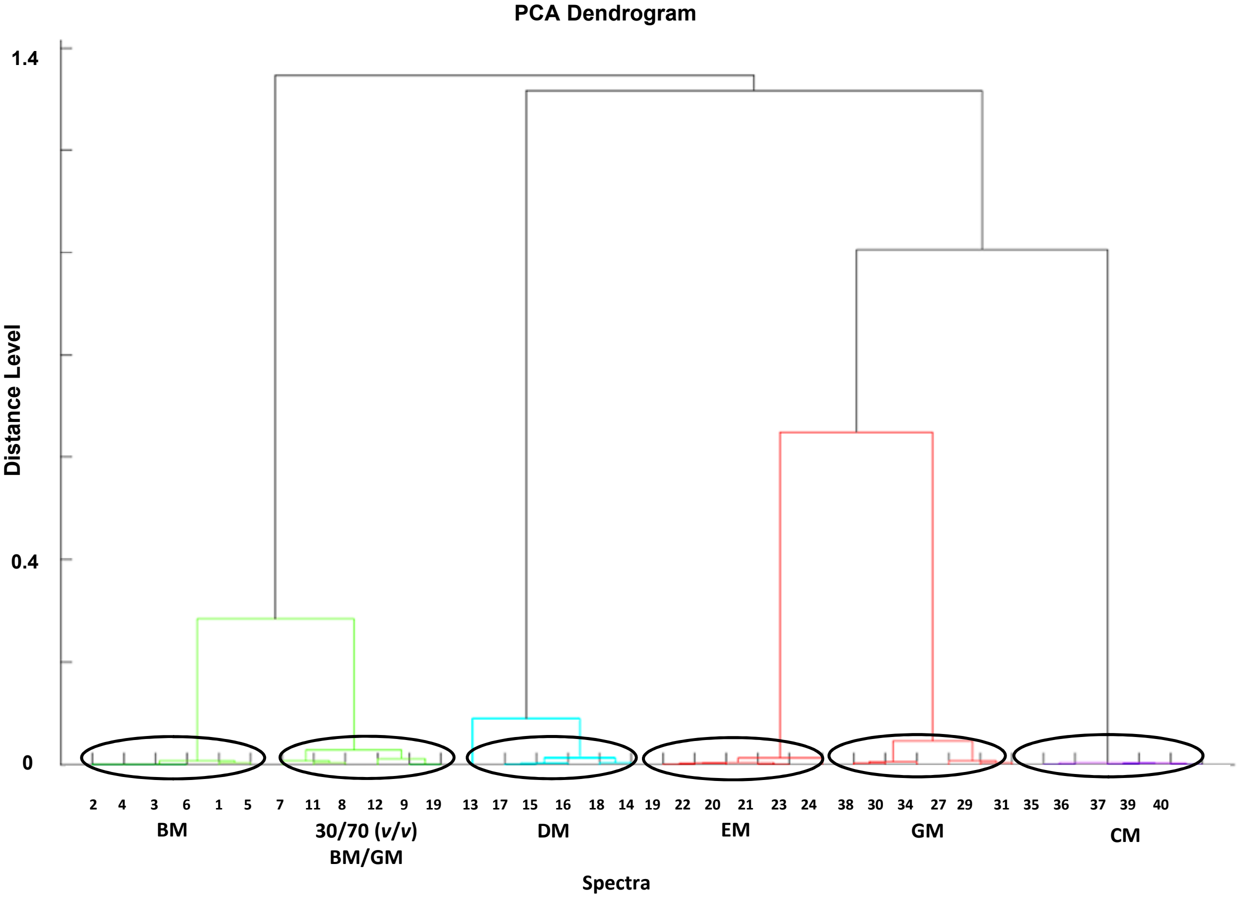

Figure S8. *Cont.*

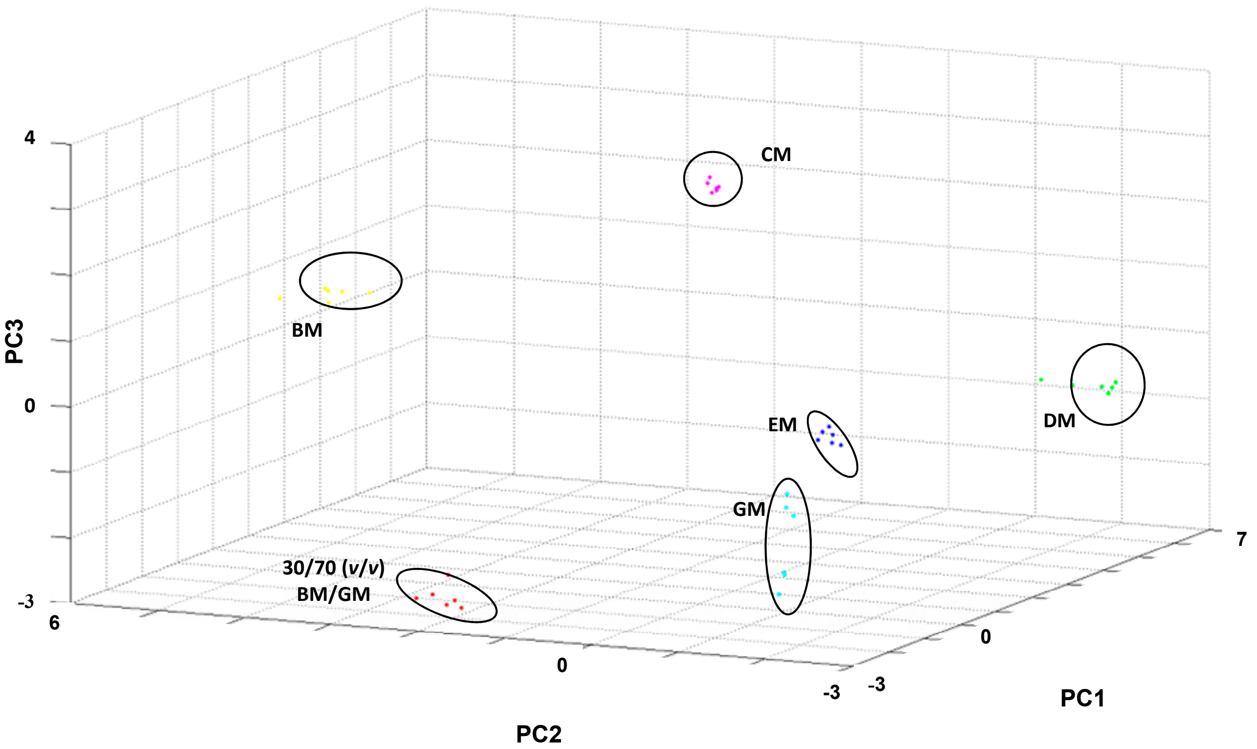

(b)

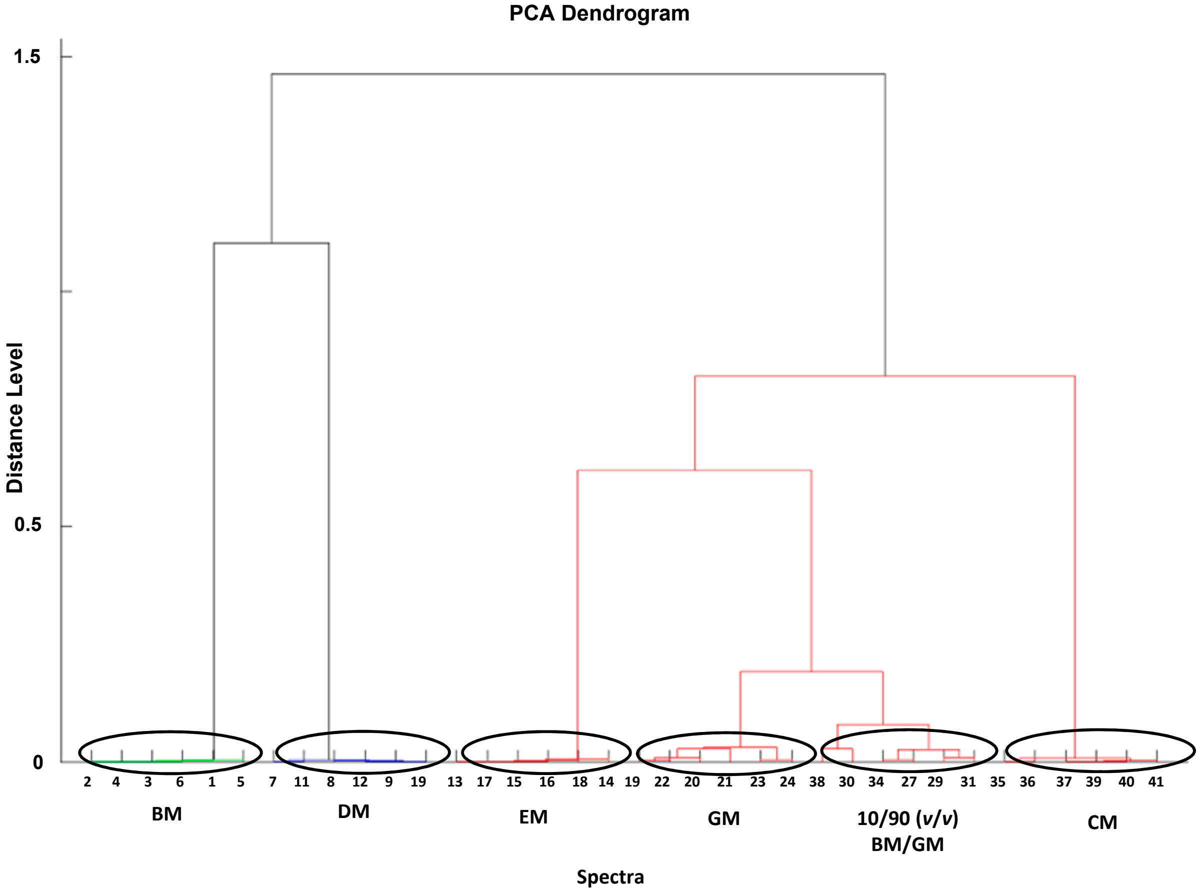

Figure S8. *Cont.*

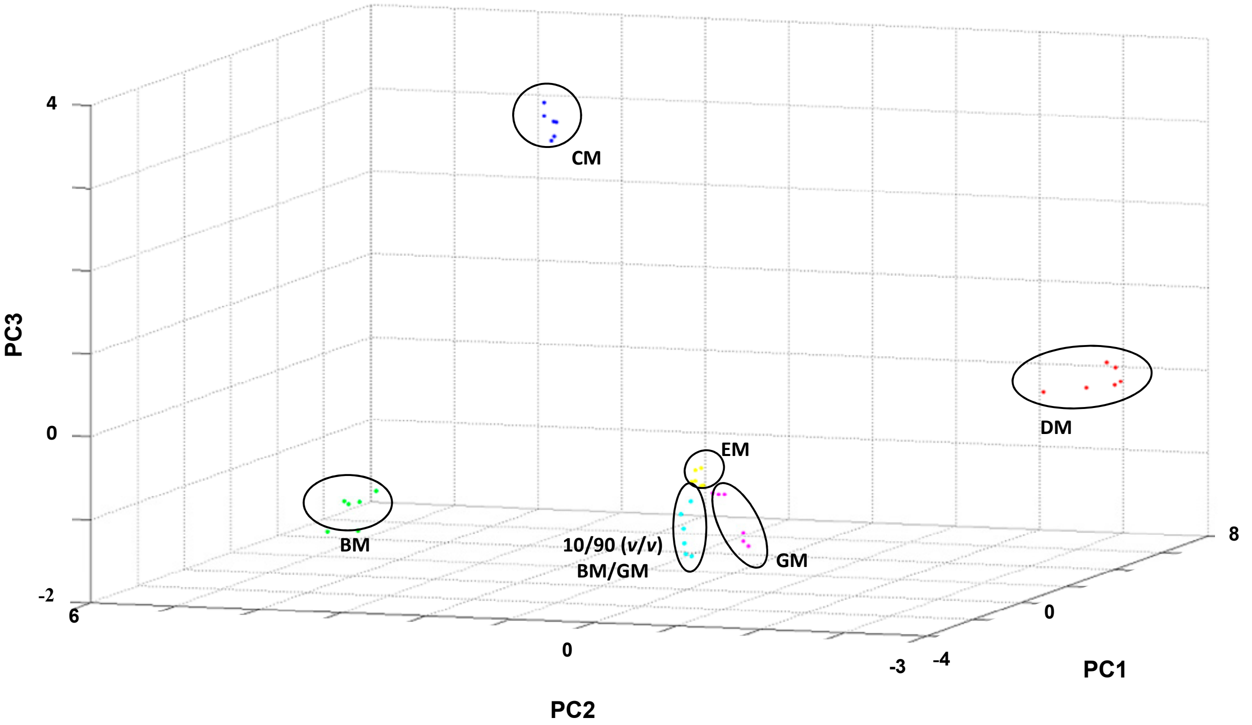

(c)

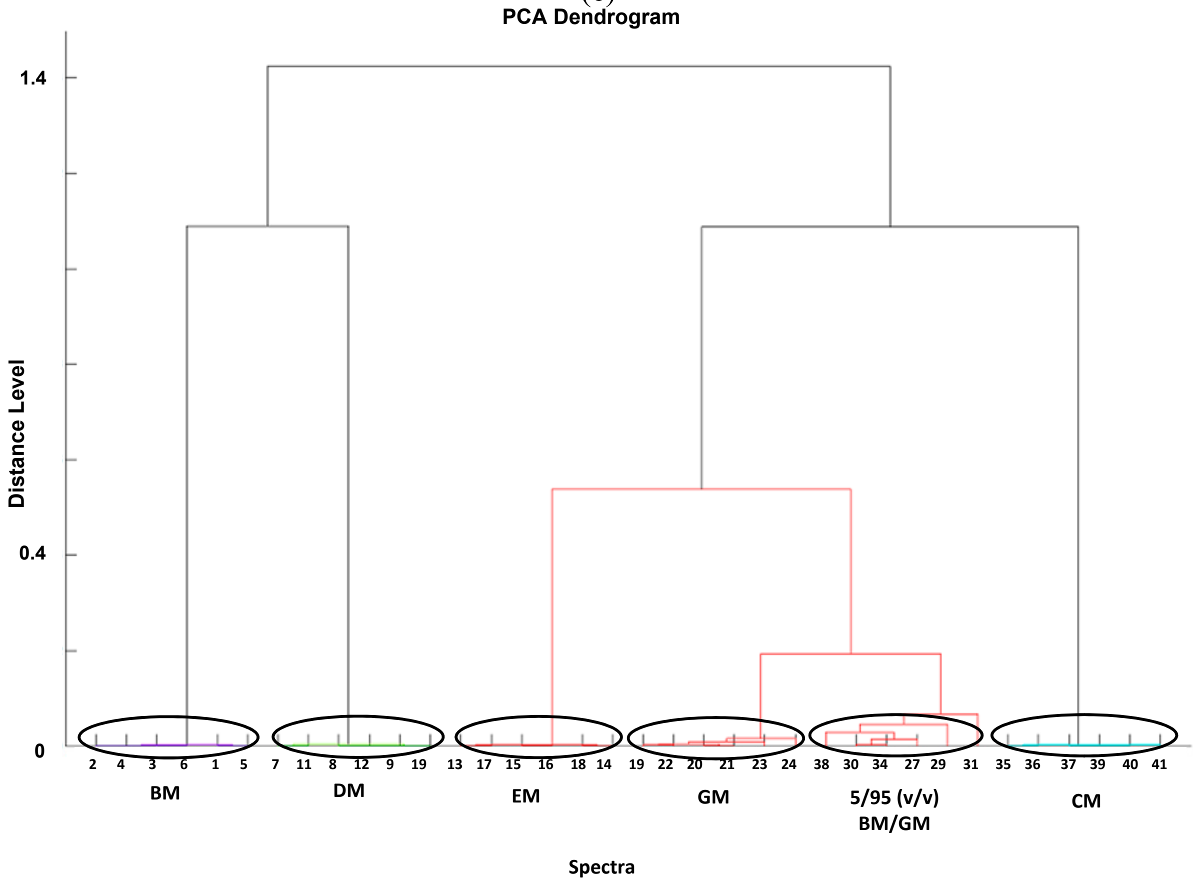

Figure S8. *Cont.*

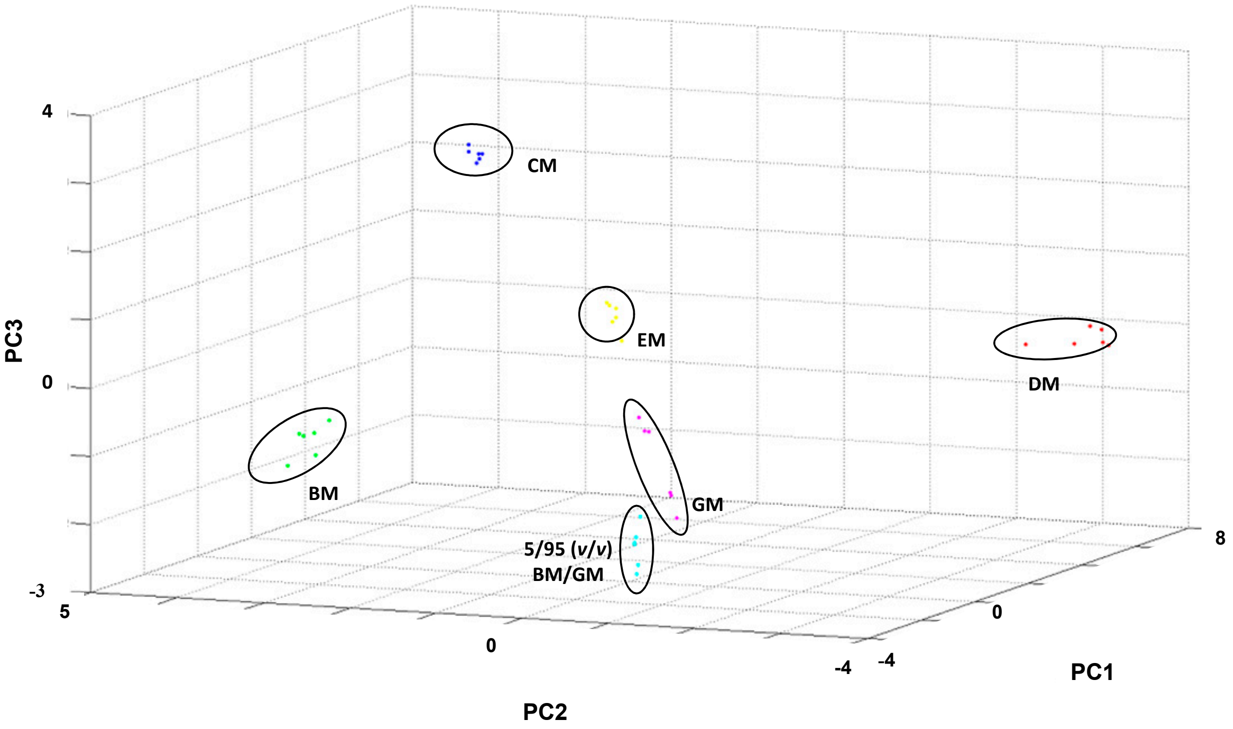

(d)

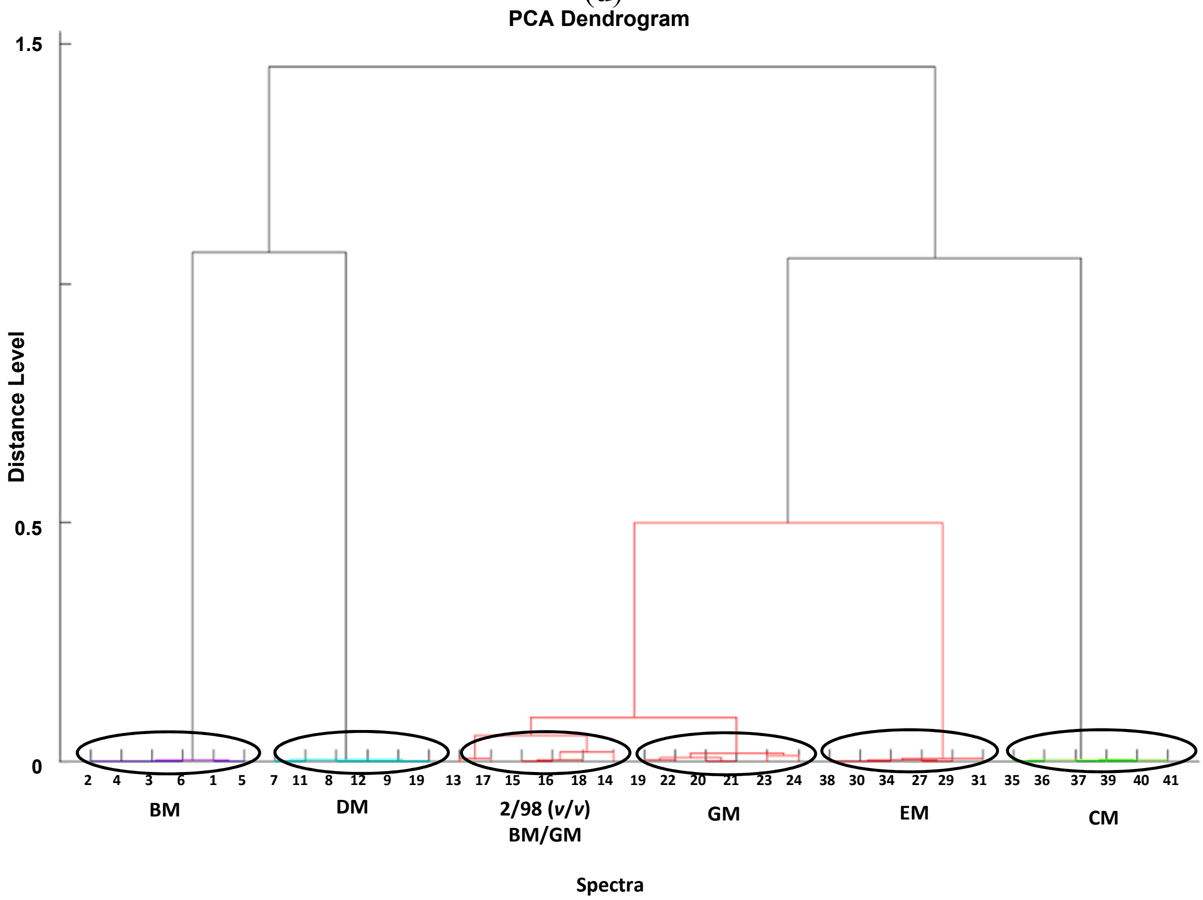

Figure S8. *Cont.*

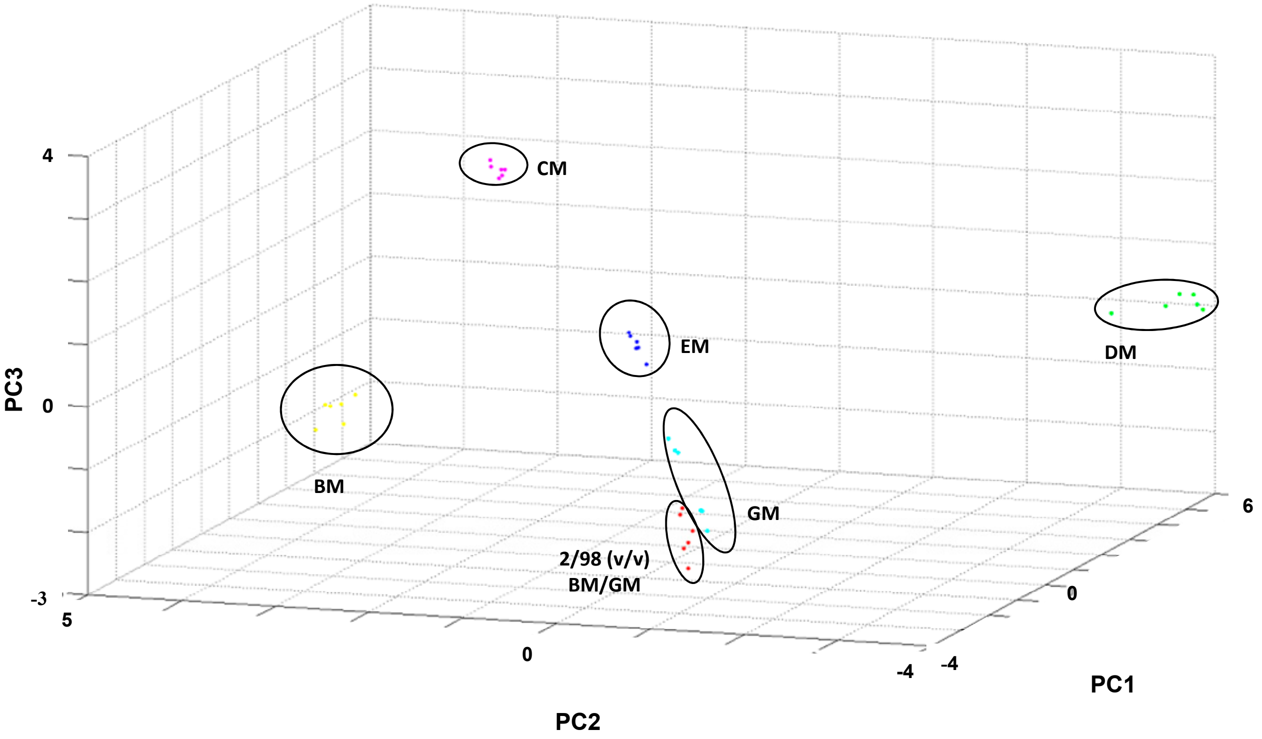

(e)

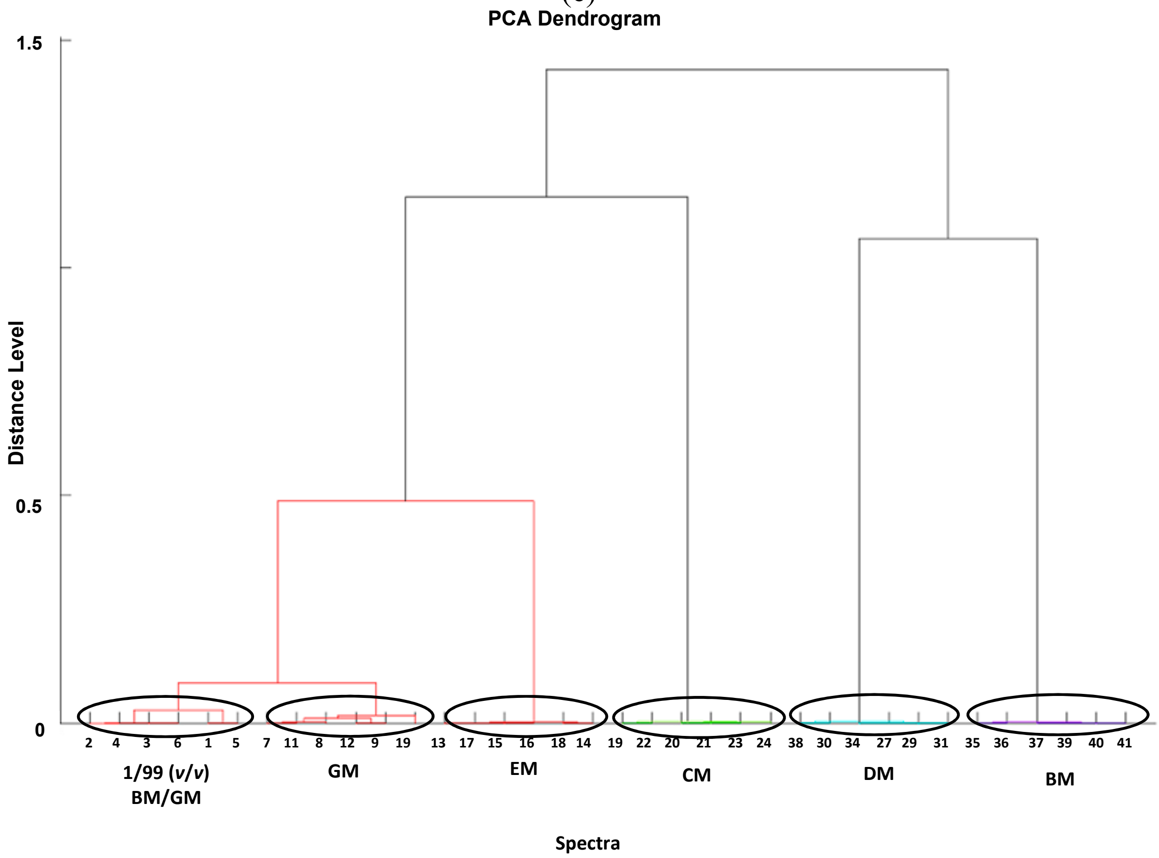

Figure S8. *Cont.*

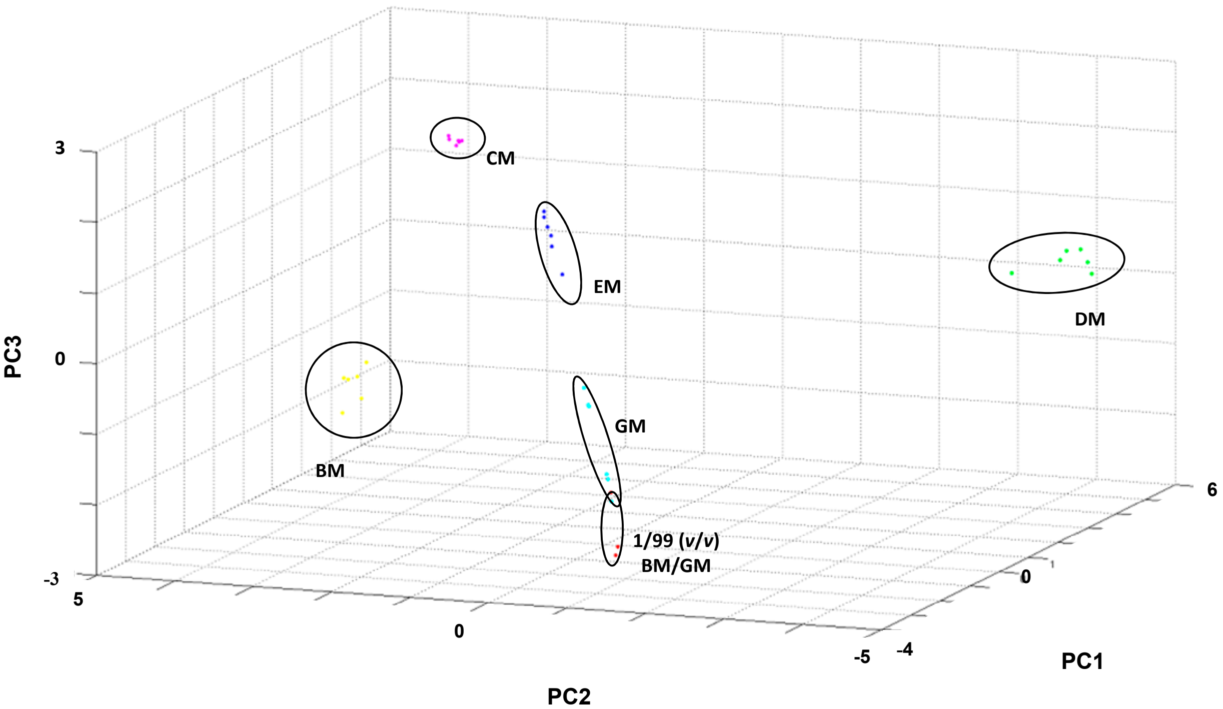

(f)

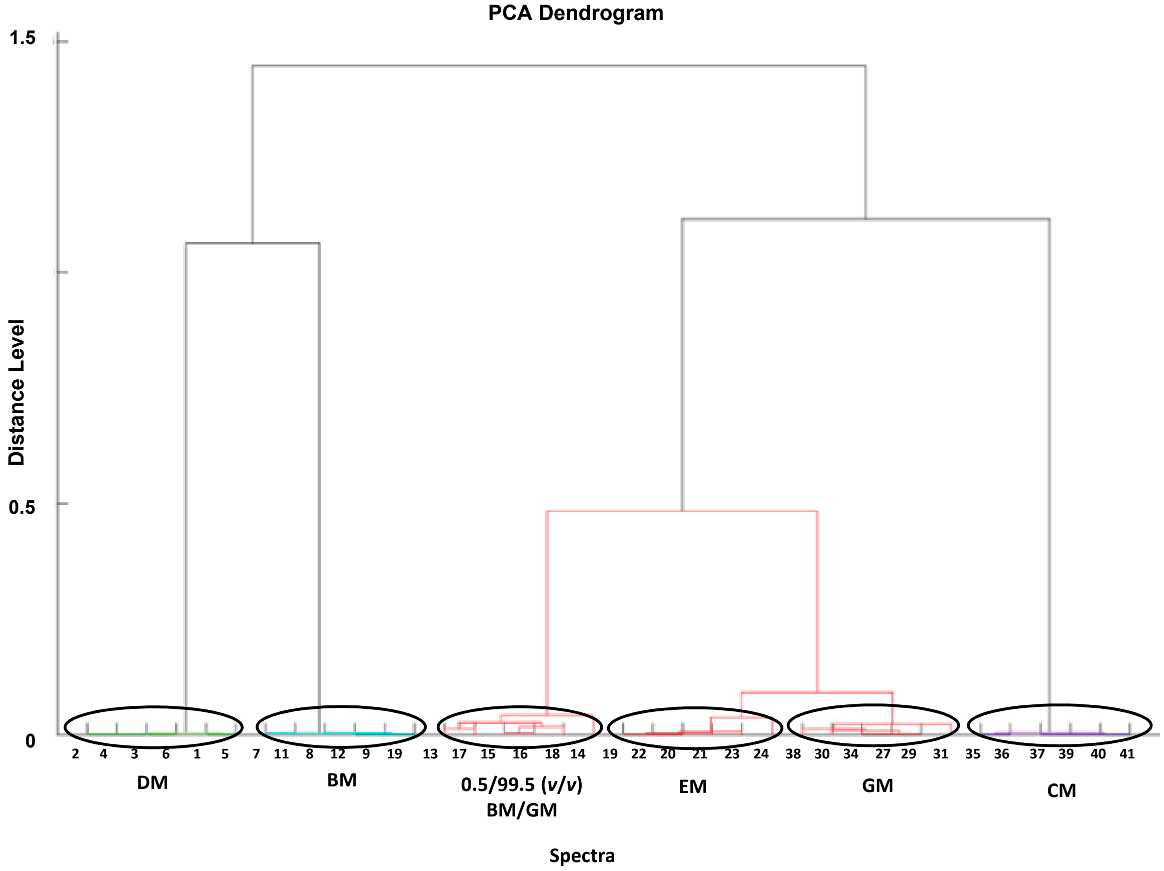

Figure S8. *Cont.*

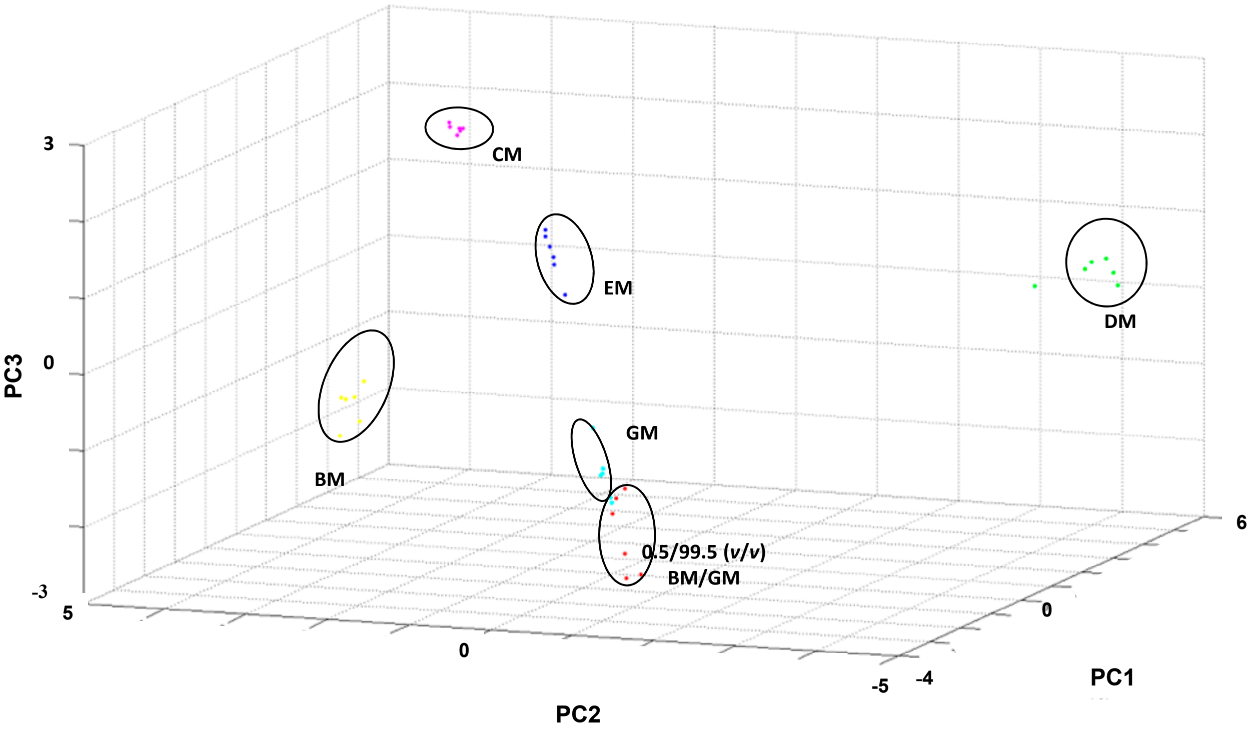

(g)

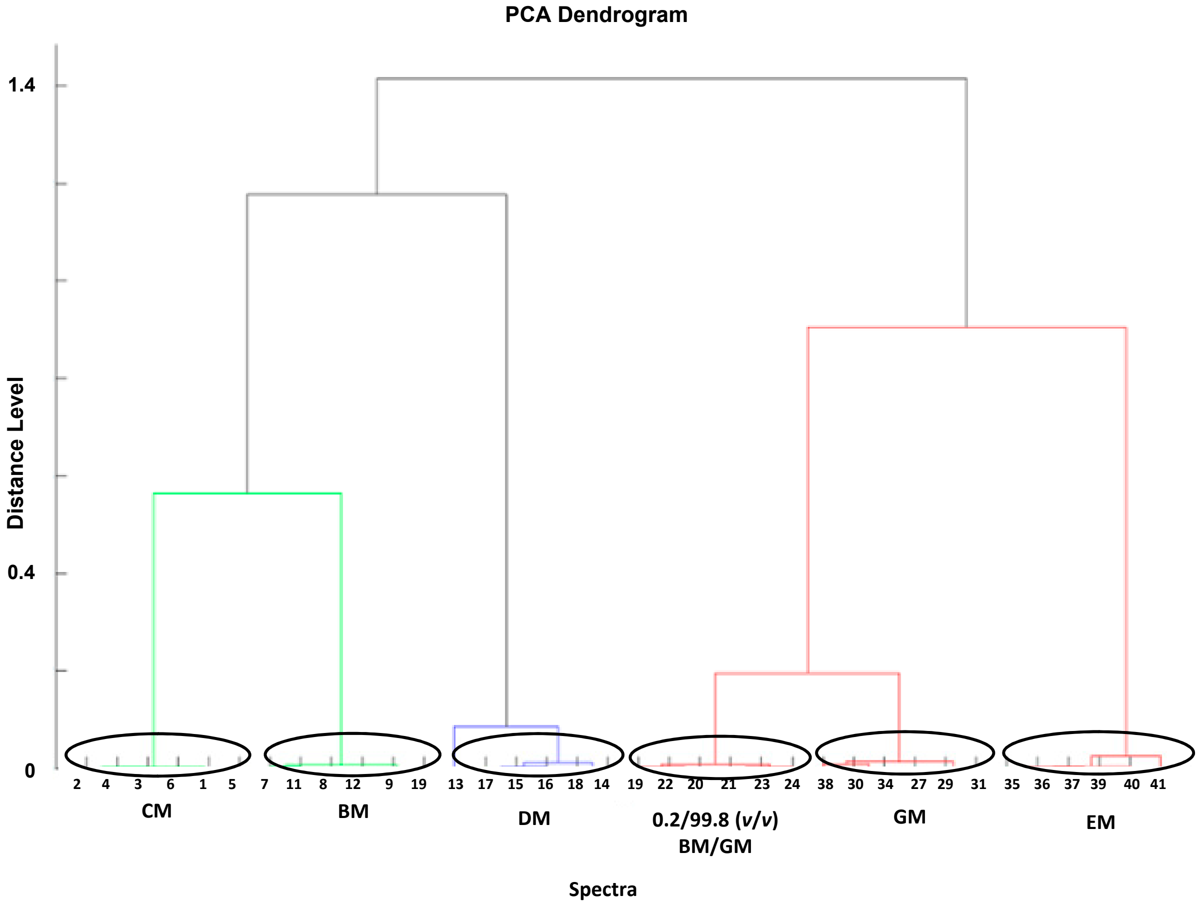

Figure S8. *Cont.*

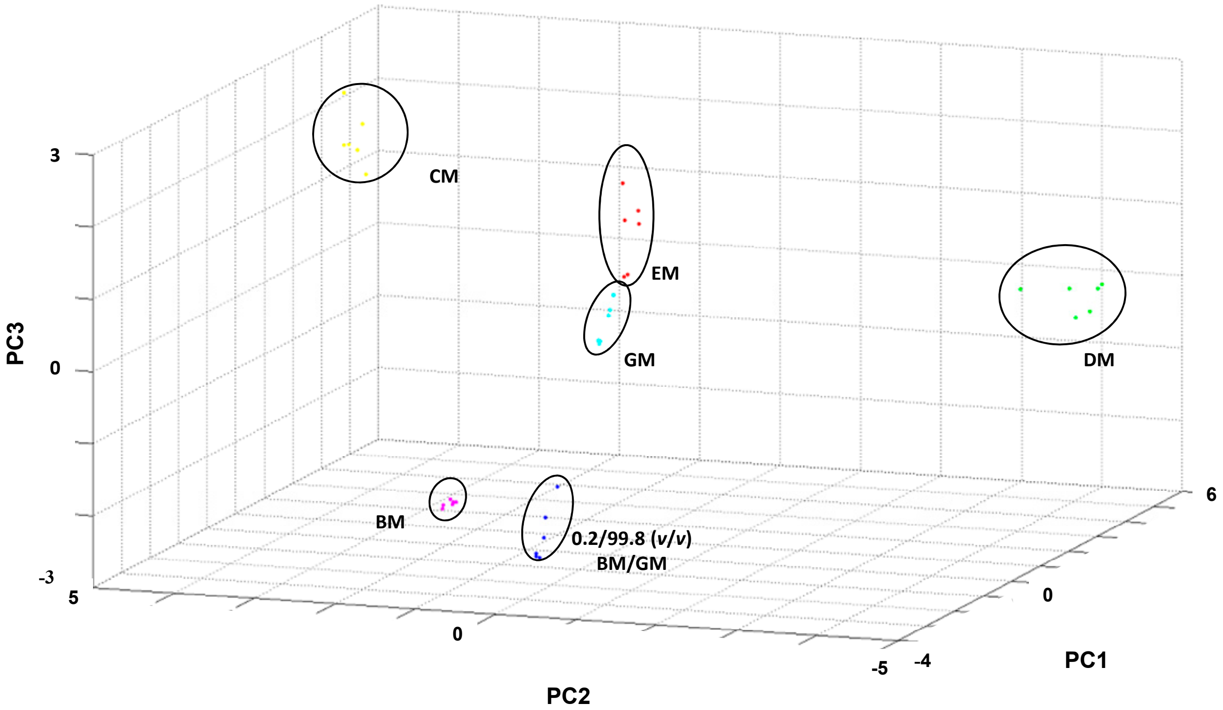

(h)

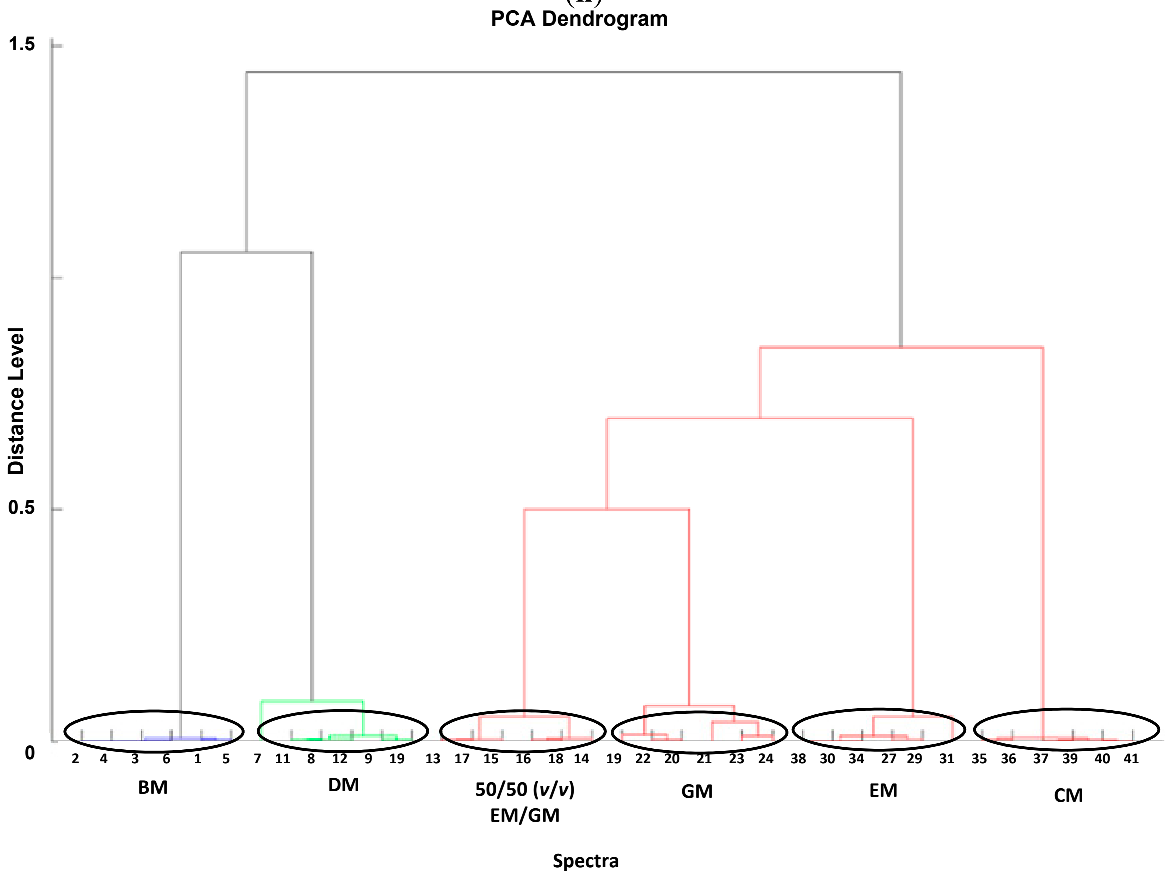

Figure S8. *Cont.*

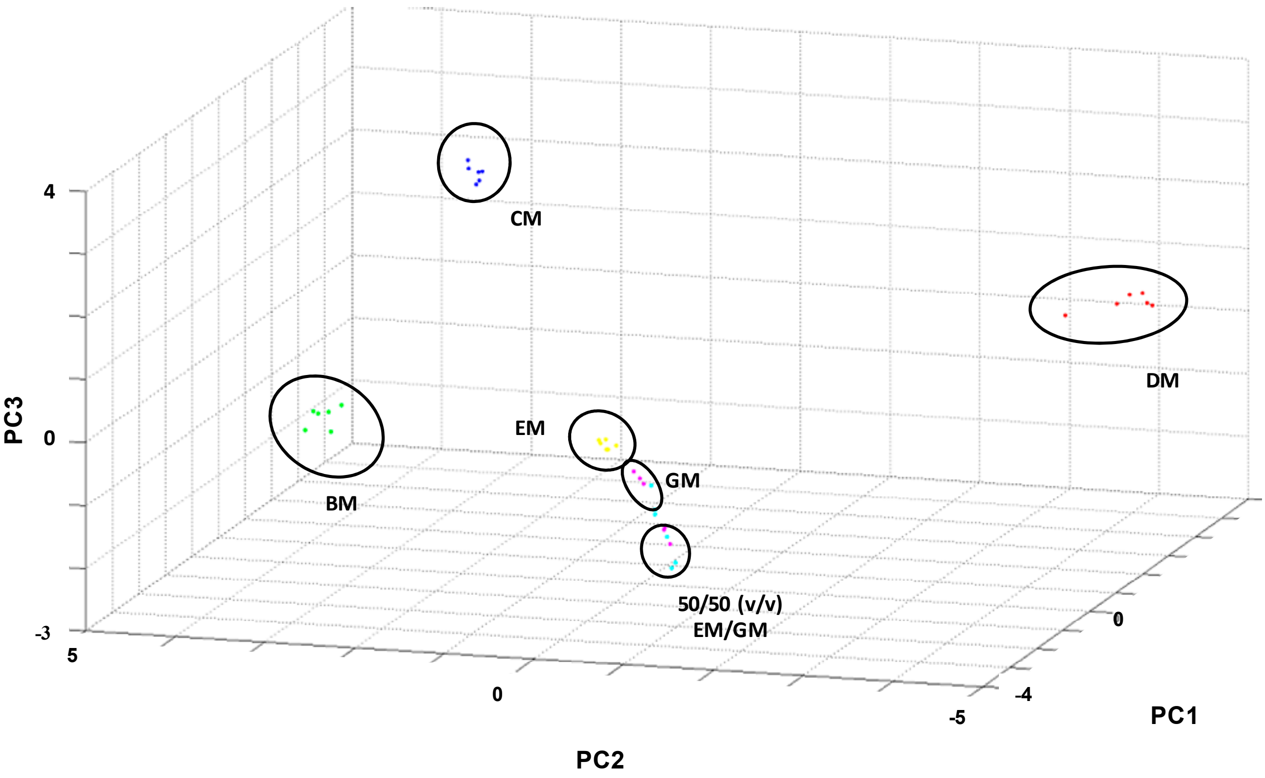

(i)

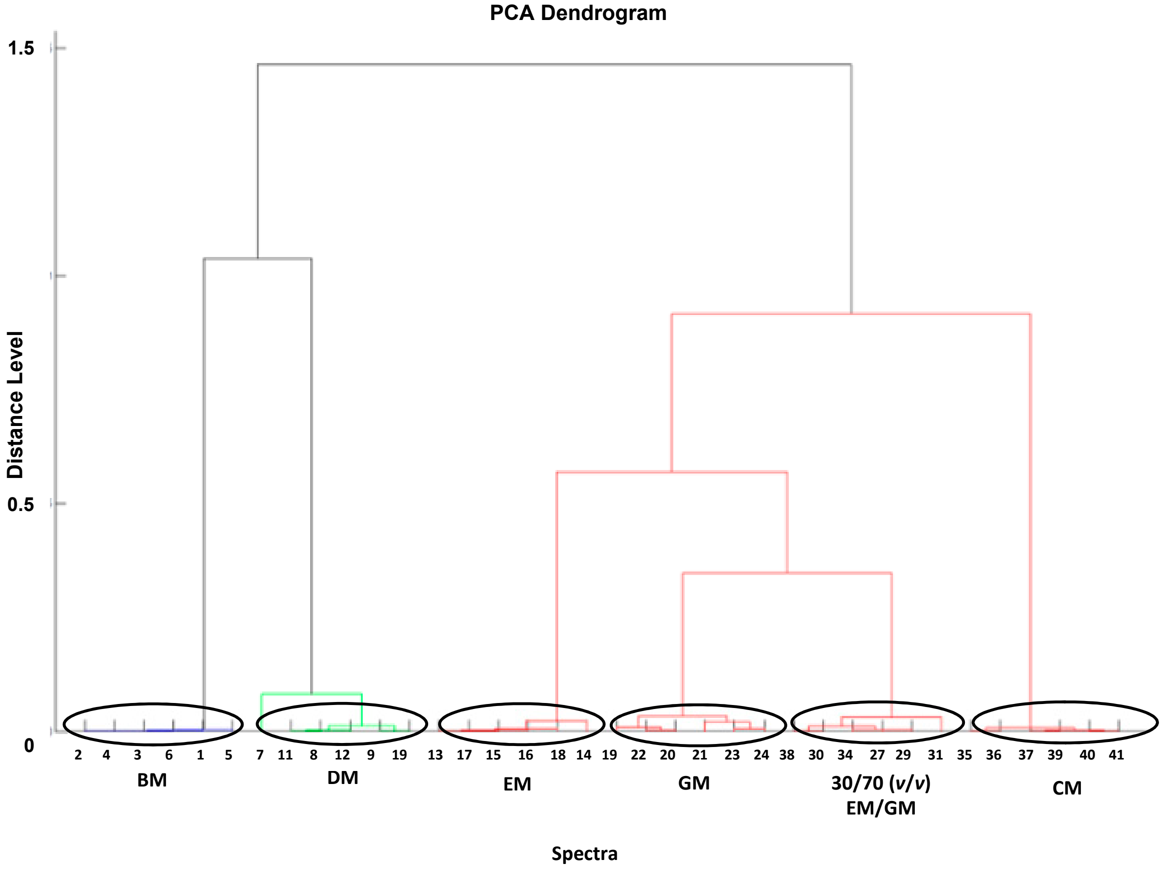

Figure S8. *Cont.*

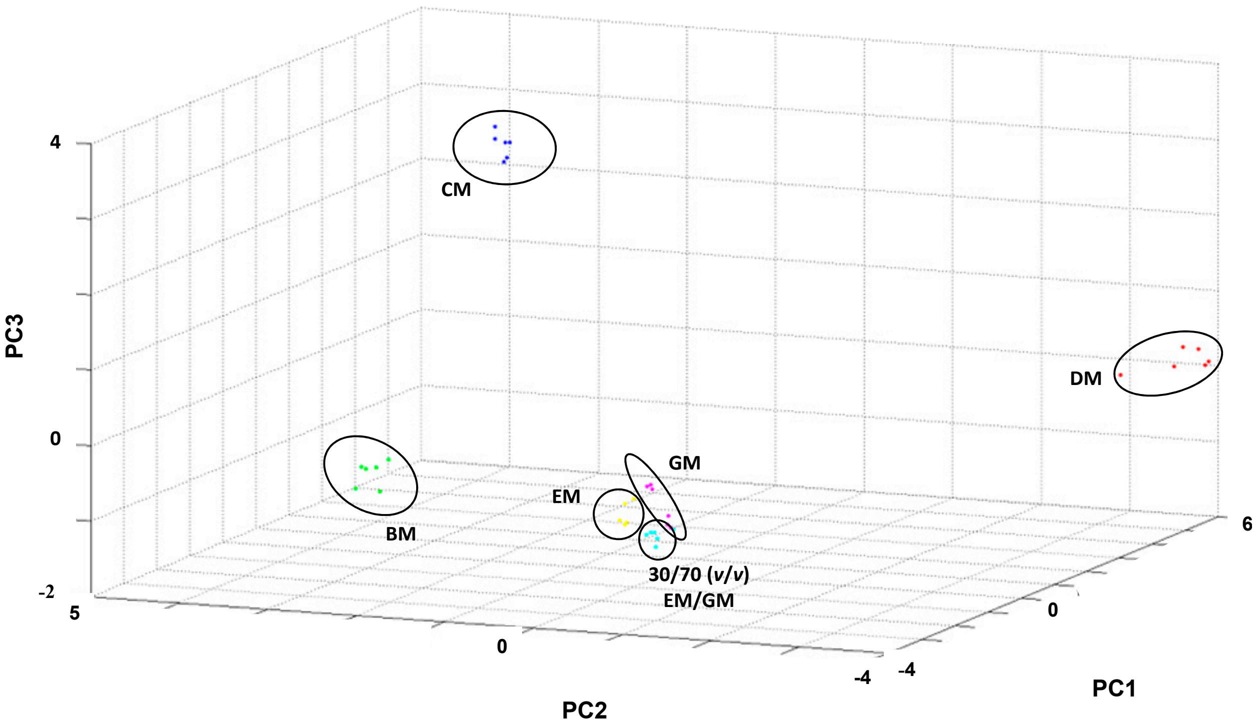

(I)

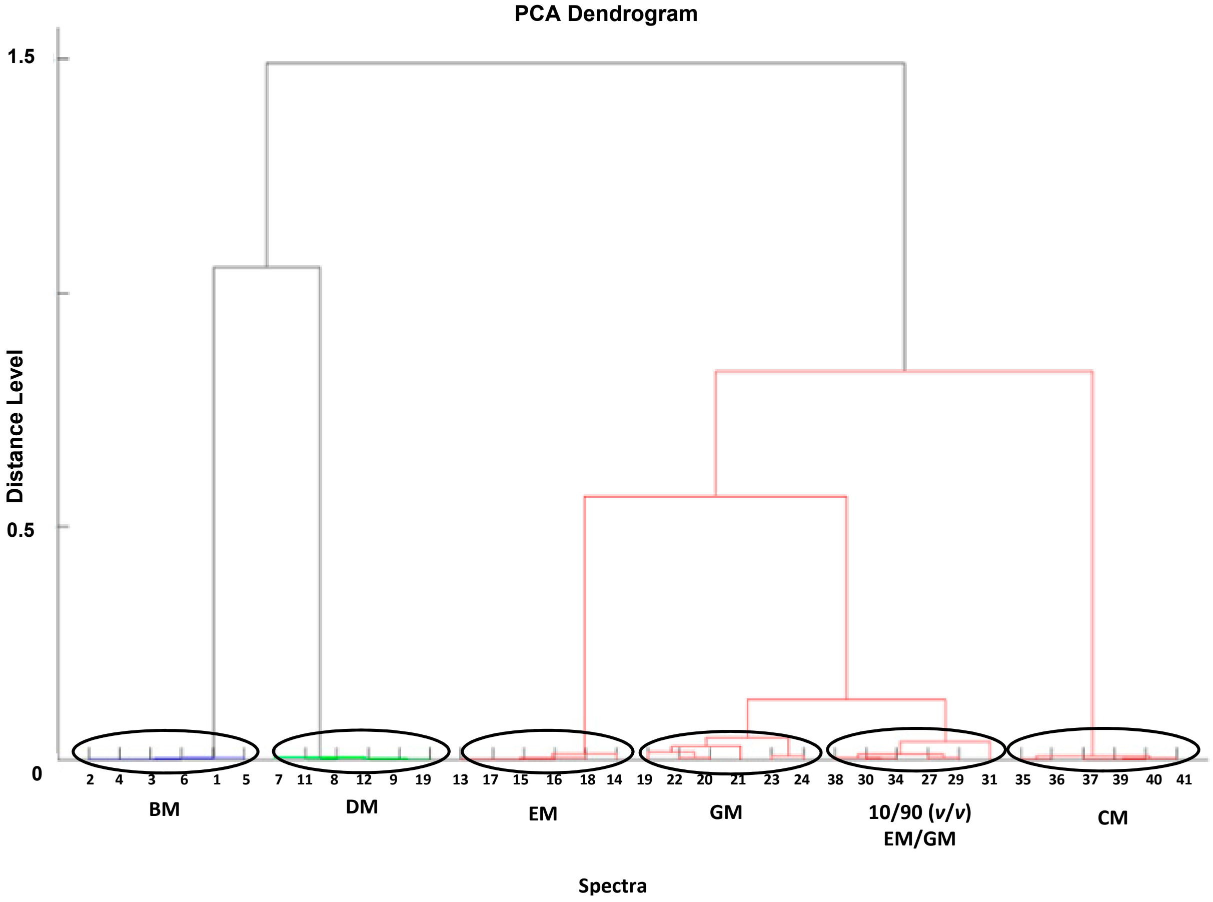

Figure S8. *Cont.*

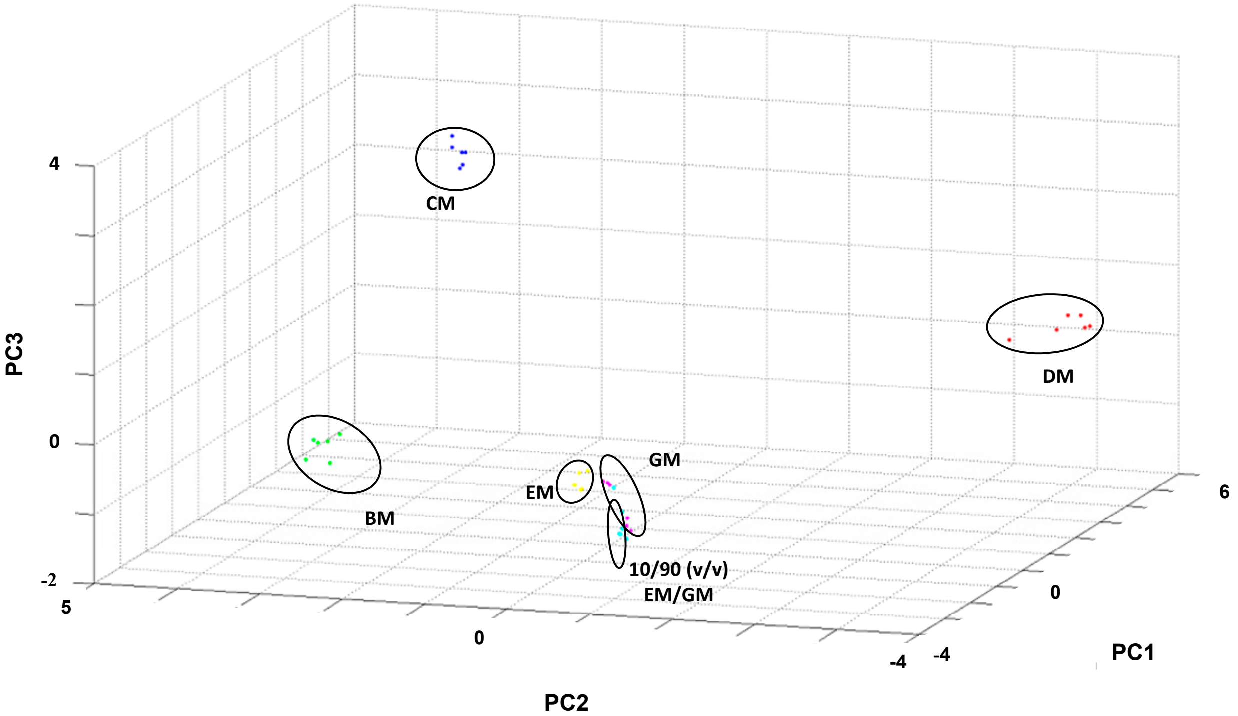

(m)

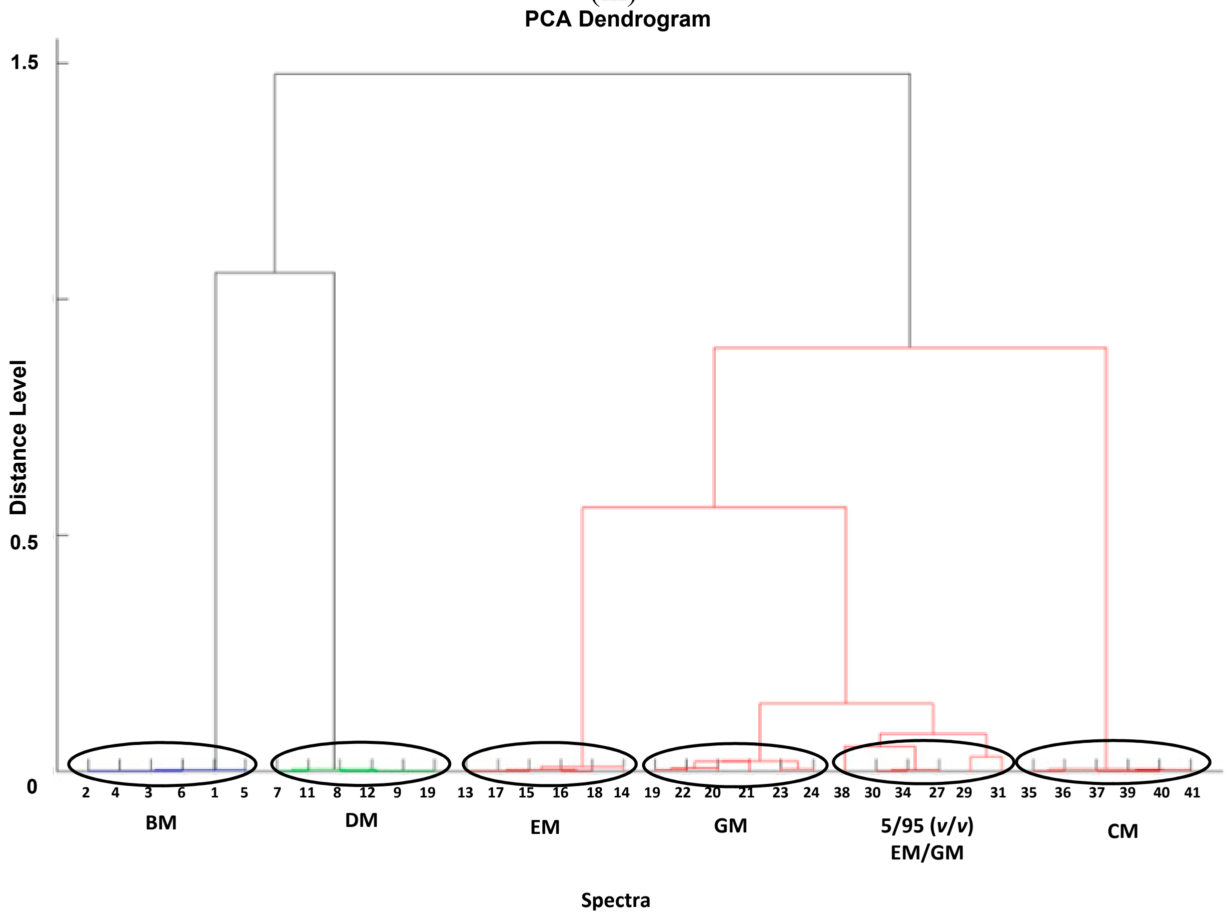

Figure S8. *Cont.*

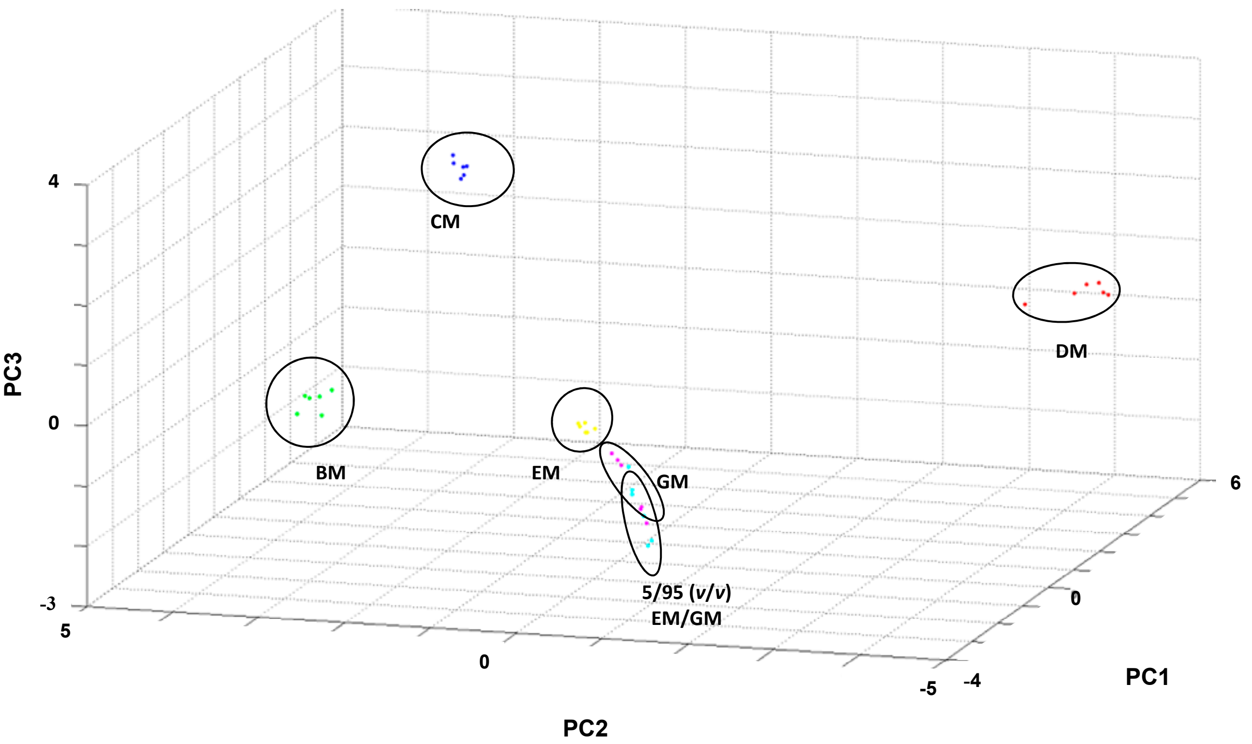

(n)

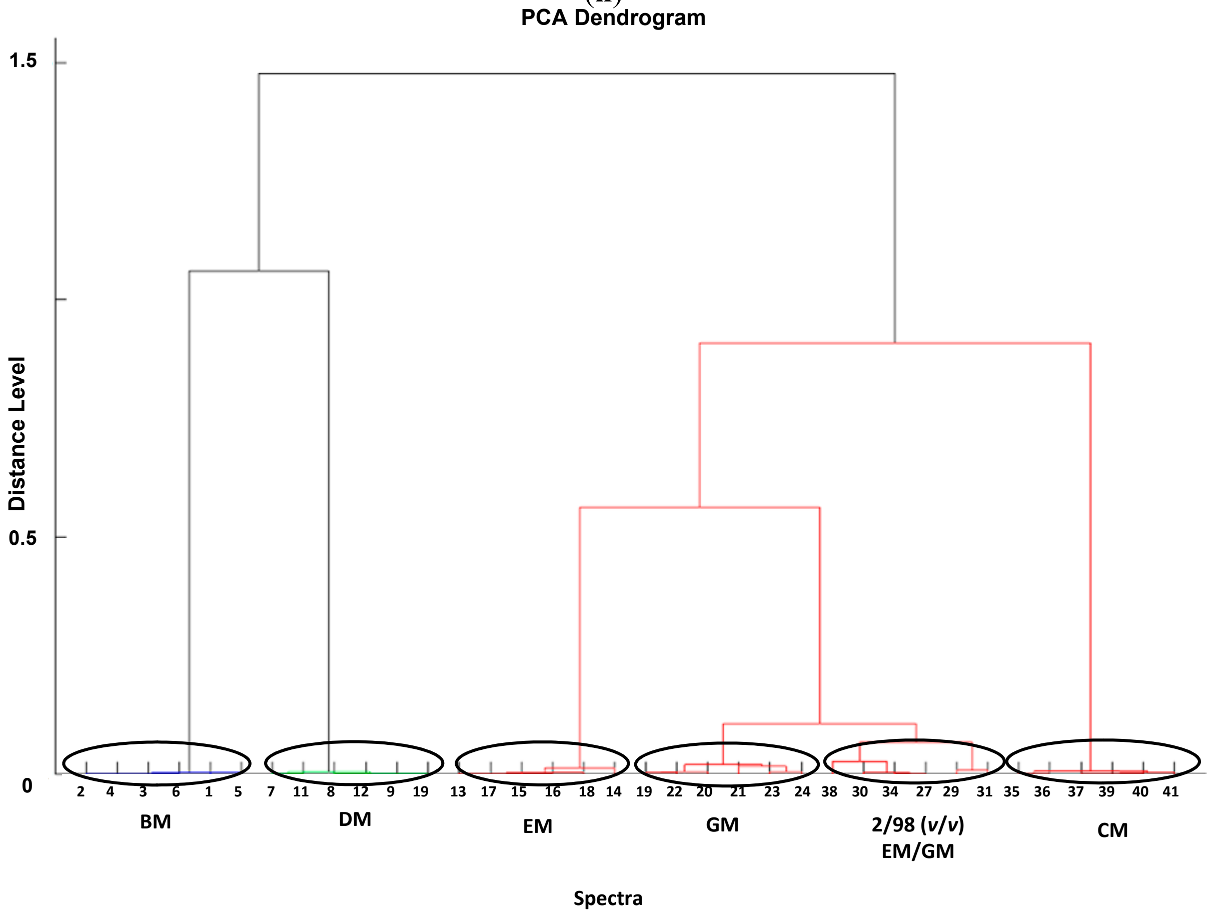

Figure S8. *Cont.*

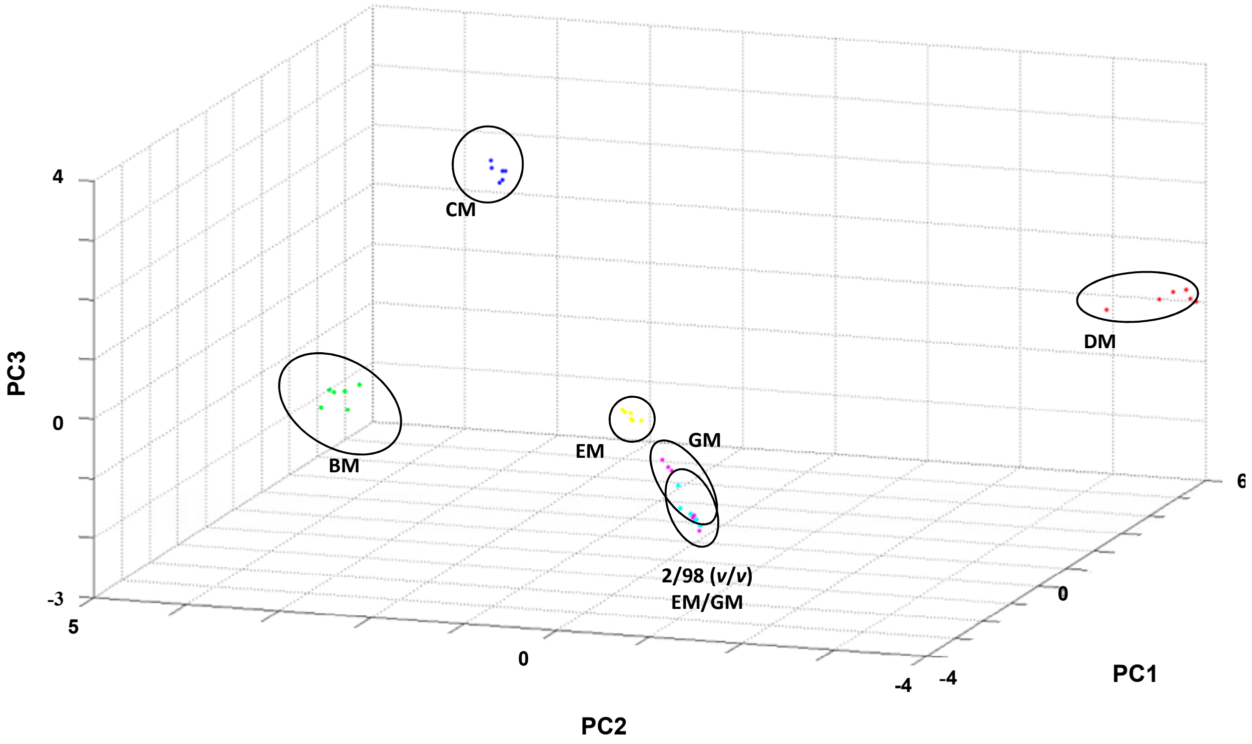

(o)

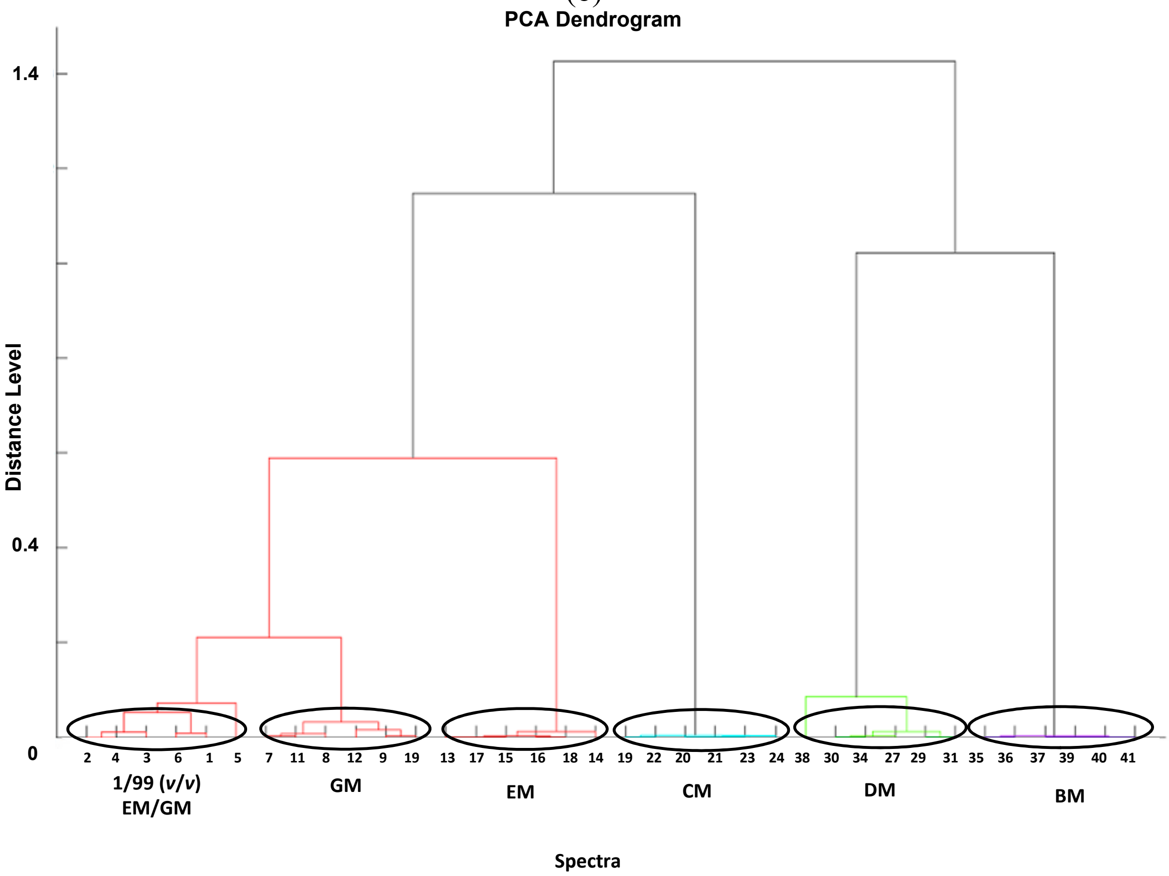

Figure S8. *Cont.*

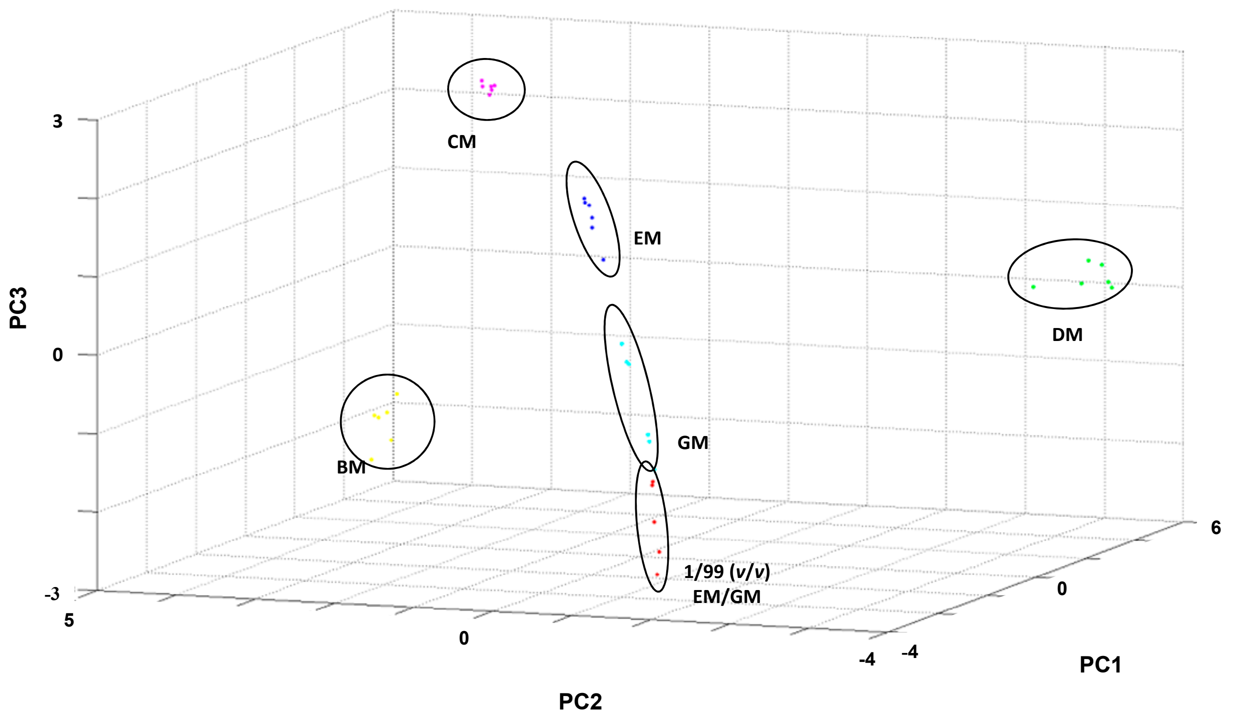

(p)

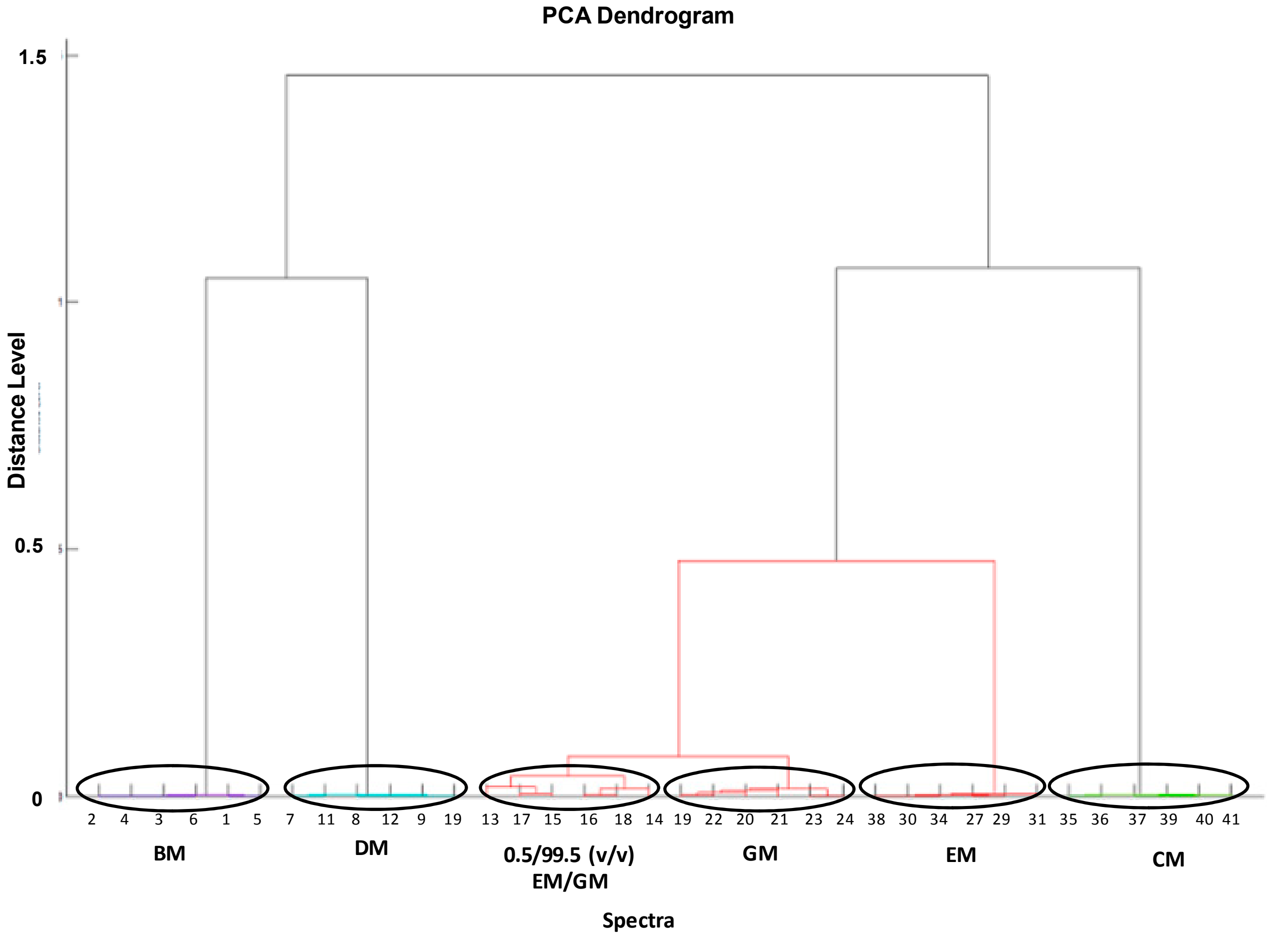

Figure S8. *Cont.*

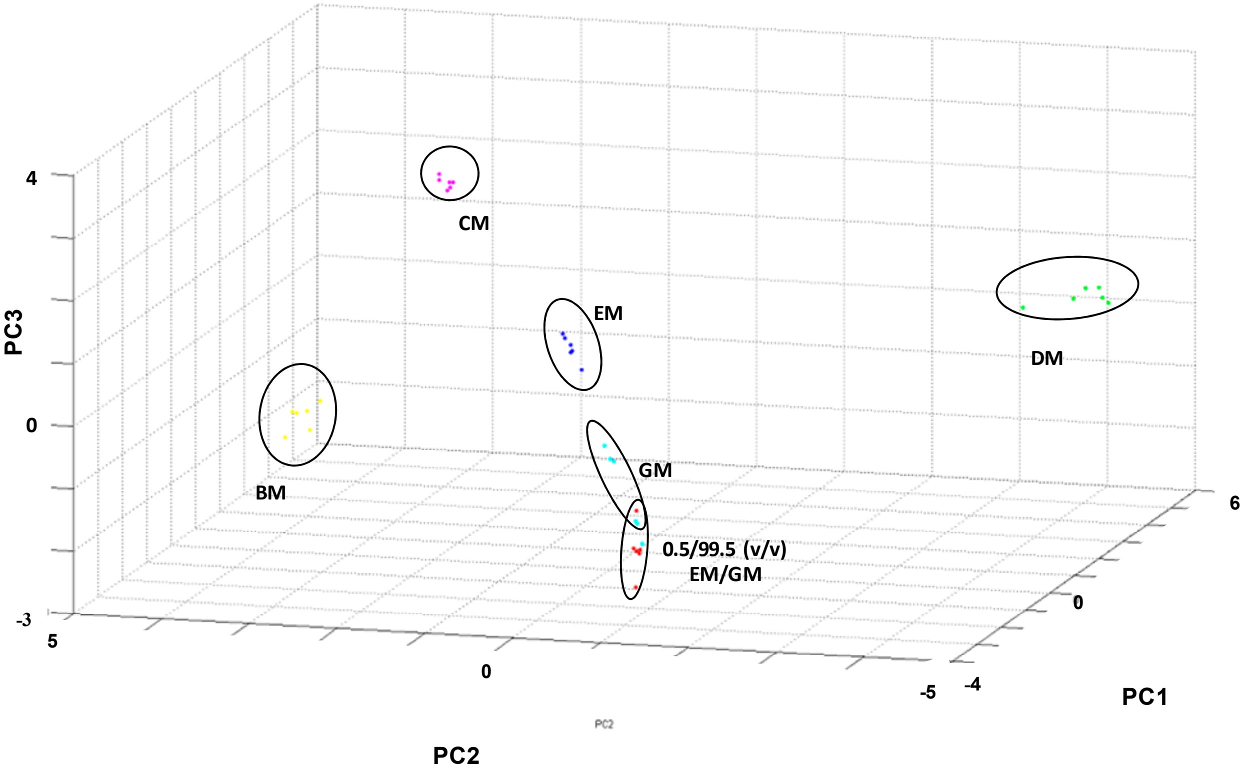

(q)

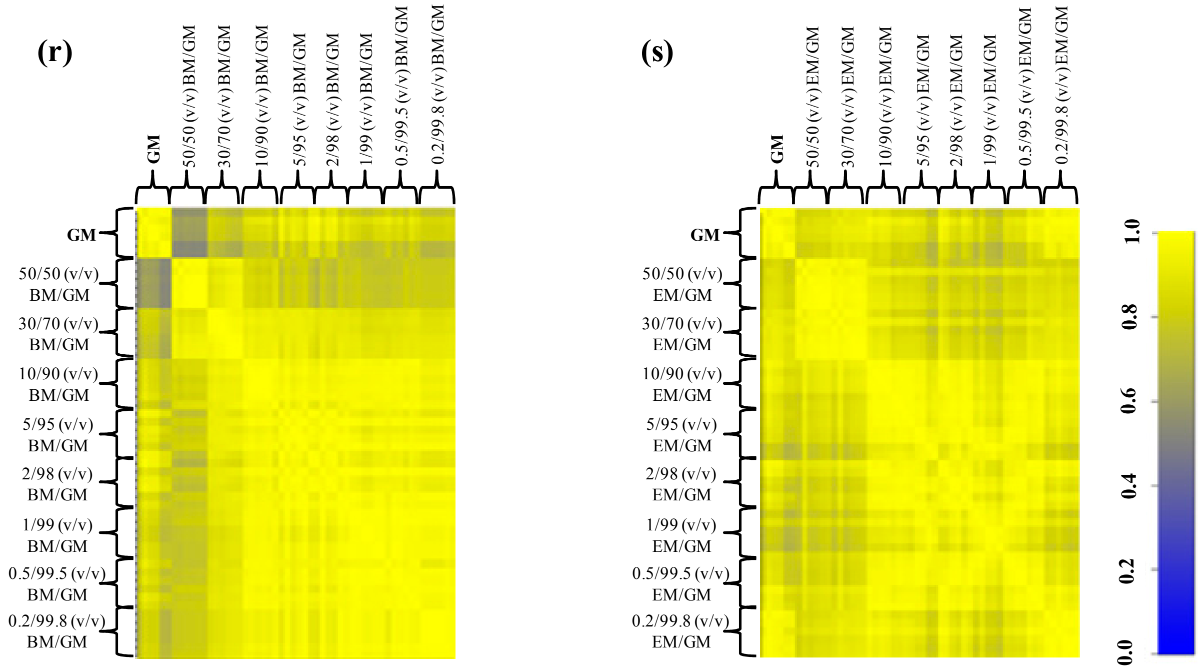

Supplement: Supplementary File 1 [file ijms-15-13697-s001.pdf]
